# Predicting the environmental suitability for onchocerciasis in Africa as an aid to elimination planning

**DOI:** 10.1371/journal.pntd.0008824

**Published:** 2021-07-28

**Authors:** Elizabeth A. Cromwell, Joshua C. P. Osborne, Thomas R. Unnasch, Maria-Gloria Basáñez, Katherine M. Gass, Kira A. Barbre, Elex Hill, Kimberly B. Johnson, Katie M. Donkers, Shreya Shirude, Chris A. Schmidt, Victor Adekanmbi, Olatunji O. Adetokunboh, Mohsen Afarideh, Ehsan Ahmadpour, Muktar Beshir Ahmed, Temesgen Yihunie Akalu, Ziyad Al-Aly, Fahad Mashhour Alanezi, Turki M. Alanzi, Vahid Alipour, Catalina Liliana Andrei, Fereshteh Ansari, Mustafa Geleto Ansha, Davood Anvari, Seth Christopher Yaw Appiah, Jalal Arabloo, Benjamin F. Arnold, Marcel Ausloos, Martin Amogre Ayanore, Atif Amin Baig, Maciej Banach, Aleksandra Barac, Till Winfried Bärnighausen, Mohsen Bayati, Krittika Bhattacharyya, Zulfiqar A. Bhutta, Sadia Bibi, Ali Bijani, Somayeh Bohlouli, Mahdi Bohluli, Oliver J. Brady, Nicola Luigi Bragazzi, Zahid A. Butt, Felix Carvalho, Souranshu Chatterjee, Vijay Kumar Chattu, Soosanna Kumary Chattu, Natalie Maria Cormier, Saad M. A. Dahlawi, Giovanni Damiani, Farah Daoud, Aso Mohammad Darwesh, Ahmad Daryani, Kebede Deribe, Samath Dhamminda Dharmaratne, Daniel Diaz, Hoa Thi Do, Maysaa El Sayed Zaki, Maha El Tantawi, Demelash Abewa Elemineh, Anwar Faraj, Majid Fasihi Harandi, Yousef Fatahi, Valery L. Feigin, Eduarda Fernandes, Nataliya A. Foigt, Masoud Foroutan, Richard Charles Franklin, Mohammed Ibrahim Mohialdeen Gubari, Davide Guido, Yuming Guo, Arvin Haj-Mirzaian, Kanaan Hamagharib Abdullah, Samer Hamidi, Claudiu Herteliu, Hagos Degefa de Hidru, Tarig B. Higazi, Naznin Hossain, Mehdi Hosseinzadeh, Mowafa Househ, Olayinka Stephen Ilesanmi, Milena D. Ilic, Irena M. Ilic, Usman Iqbal, Seyed Sina Naghibi Irvani, Ravi Prakash Jha, Farahnaz Joukar, Jacek Jerzy Jozwiak, Zubair Kabir, Leila R. Kalankesh, Rohollah Kalhor, Behzad Karami Matin, Salah Eddin Karimi, Amir Kasaeian, Taras Kavetskyy, Gbenga A. Kayode, Ali Kazemi Karyani, Abraham Getachew Kelbore, Maryam Keramati, Rovshan Khalilov, Ejaz Ahmad Khan, Md Nuruzzaman Nuruzzaman Khan, Khaled Khatab, Mona M. Khater, Neda Kianipour, Kelemu Tilahun Kibret, Yun Jin Kim, Soewarta Kosen, Kris J. Krohn, Dian Kusuma, Carlo La Vecchia, Van Charles Lansingh, Paul H. Lee, Kate E. LeGrand, Shanshan Li, Joshua Longbottom, Hassan Magdy Abd El Razek, Muhammed Magdy Abd El Razek, Afshin Maleki, Abdullah A. Mamun, Ali Manafi, Navid Manafi, Mohammad Ali Mansournia, Francisco Rogerlândio Martins-Melo, Mohsen Mazidi, Colm McAlinden, Birhanu Geta Meharie, Walter Mendoza, Endalkachew Worku Mengesha, Desalegn Tadese Mengistu, Seid Tiku Mereta, Tomislav Mestrovic, Ted R. Miller, Mohammad Miri, Masoud Moghadaszadeh, Abdollah Mohammadian-Hafshejani, Reza Mohammadpourhodki, Shafiu Mohammed, Salahuddin Mohammed, Masoud Moradi, Rahmatollah Moradzadeh, Paula Moraga, Jonathan F. Mosser, Mehdi Naderi, Ahamarshan Jayaraman Nagarajan, Gurudatta Naik, Ionut Negoi, Cuong Tat Nguyen, Huong Lan Thi Nguyen, Trang Huyen Nguyen, Rajan Nikbakhsh, Bogdan Oancea, Tinuke O. Olagunju, Andrew T. Olagunju, Ahmed Omar Bali, Obinna E. Onwujekwe, Adrian Pana, Hadi Pourjafar, Fakher Rahim, Mohammad Hifz Ur Rahman, Priya Rathi, Salman Rawaf, David Laith Rawaf, Reza Rawassizadeh, Serge Resnikoff, Melese Abate Reta, Aziz Rezapour, Enrico Rubagotti, Salvatore Rubino, Ehsan Sadeghi, Abedin Saghafipour, S. Mohammad Sajadi, Abdallah M. Samy, Rodrigo Sarmiento-Suárez, Monika Sawhney, Megan F. Schipp, Amira A. Shaheen, Masood Ali Shaikh, Morteza Shamsizadeh, Kiomars Sharafi, Aziz Sheikh, B. Suresh Kumar Shetty, Jae Il Shin, K. M. Shivakumar, Biagio Simonetti, Jasvinder A. Singh, Eirini Skiadaresi, Amin Soheili, Shahin Soltani, Emma Elizabeth Spurlock, Mu’awiyyah Babale Sufiyan, Takahiro Tabuchi, Leili Tapak, Robert L. Thompson, Alan J. Thomson, Eugenio Traini, Bach Xuan Tran, Irfan Ullah, Saif Ullah, Chigozie Jesse Uneke, Bhaskaran Unnikrishnan, Olalekan A. Uthman, Natalie V. S. Vinkeles Melchers, Francesco S. Violante, Haileab Fekadu Wolde, Tewodros Eshete Wonde, Tomohide Yamada, Sanni Yaya, Vahid Yazdi-Feyzabadi, Paul Yip, Naohiro Yonemoto, Hebat-Allah Salah A. Yousof, Chuanhua Yu, Yong Yu, Hasan Yusefzadeh, Leila Zaki, Sojib Bin Zaman, Maryam Zamanian, Zhi-Jiang Zhang, Yunquan Zhang, Arash Ziapour, Simon I. Hay, David M. Pigott

**Affiliations:** 1 Institute for Health Metrics and Evaluation, University of Washington, Seattle, Washington, United States of America; 2 Department of Health Metrics Sciences, School of Medicine, University of Washington, Seattle, Washington, United States of America; 3 GlobalHealth Infectious Disease, University of South Florida, Tampa, Florida, United States of America; 4 London Centre for Neglected Tropical Disease Research (LCNTDR), Imperial College London, London, United Kingdom; 5 MRC Centre for Global Infectious Disease Analysis (MRC-GIDA), Imperial College London, London, United Kingdom; 6 Neglected Tropical Diseases Support Center, Task Force for Global Health, Decatur, Georgia, United States of America; 7 Population Health Sciences, King’s College London, London, England; 8 Centre of Excellence for Epidemiological Modelling and Analysis, Stellenbosch University, Stellenbosch, South Africa; 9 Department of Global Health, Stellenbosch University, Cape Town, South Africa; 10 Department of Dermatology, Mayo Clinic, Rochester, Minnesota, United States of America; 11 Endocrinology and Metabolism Research Center, Tehran University of Medical Sciences, Tehran, Iran; 12 Infectious and Tropical Diseases Research Center, Tabriz University of Medical Sciences, Tabriz, Iran; 13 Department of Epidemiology, Jimma University, Jimma, Ethiopia; 14 Australian Center for Precision Health, University of South Australia, Adelaide, South Australia, Australia; 15 Department of Epidemiology and Biostatistics, University of Gondar, Gondar, Ethiopia; 16 John T. Milliken Department of Internal Medicine, Washington University in St. Louis, St. Louis, Montana, United States of America; 17 Clinical Epidemiology Center, Department of Veterans Affairs, St Louis, Montana, United States of America; 18 Imam Abdulrahman Bin Faisal University, Dammam, Saudi Arabia; 19 Health Information Management and Technology Department, Imam Abdulrahman Bin Faisal University, Dammam, Saudi Arabia; 20 Health Management and Economics Research Center, Iran University of Medical Sciences, Tehran, Iran; 21 Health Economics Department, Iran University of Medical Sciences, Tehran, Iran; 22 Cardiology Department, Carol Davila University of Medicine and Pharmacy, Bucharest, Romania; 23 Research Center for Evidence Based Medicine, Tabriz University of Medical Sciences, Tabriz, Iran; 24 Razi Vaccine and Serum Research Institute, Agricultural Research, Education, and Extension Organization (AREEO), Tehran, Iran; 25 Department of Public Health, Debre Berhan University, Debre Berhan, Ethiopia; 26 Department of Parasitology, Mazandaran University of Medical Sciences, Sari, Iran; 27 Department of Parasitology, Iranshahr University of Medical Sciences, Iranshahr, Iran; 28 Department of Sociology and Social Work, Kwame Nkrumah University of Science and Technology, Kumasi, Ghana; 29 Center for International Health, Ludwig Maximilians University, Munich, Germany; 30 Department of Ophthalmology, University of California San Francisco, San Francisco, California, United States of America; 31 School of Business, University of Leicester, Leicester, United Kingdom; 32 Department of Statistics and Econometrics, Bucharest University of Economic Studies, Bucharest, Romania; 33 Department of Health Policy Planning and Management, University of Health and Allied Sciences, Ho, Ghana; 34 Unit of Biochemistry, Sultan Zainal Abidin University (Universiti Sultan Zainal Abidin), Kuala Terengganu, Malaysia; 35 Department of Hypertension, Medical University of Lodz, Lodz, Poland; 36 Polish Mothers’ Memorial Hospital Research Institute, Lodz, Poland; 37 Clinic for Infectious and Tropical Diseases, Clinical Center of Serbia, Belgrade, Serbia; 38 Faculty of Medicine, University of Belgrade, Belgrade, Serbia; 39 Heidelberg Institute of Global Health (HIGH), Heidelberg University, Heidelberg, Germany; 40 T.H. Chan School of Public Health, Harvard University, Boston, Massachusetts, United States of America; 41 Health Human Resources Research Center, Shiraz University of Medical Sciences, Shiraz, Iran; 42 Department of Statistical and Computational Genomics, National Institute of Biomedical Genomics, Kalyani, India; 43 Department of Statistics, University of Calcutta, Kolkata, India; 44 Centre for Global Child Health, University of Toronto, Toronto, Ontario, Canada; 45 Centre of Excellence in Women & Child Health, Aga Khan University, Karachi, Pakistan; 46 Institute of Soil and Environmental Sciences, University of Agriculture—Faisalabad, Faisalabad, Pakistan; 47 Social Determinants of Health Research Center, Babol University of Medical Sciences, Babol, Iran; 48 Department of Veterinary Medicine, Islamic Azad University, Kermanshah, Iran; 49 Department of Computer Science and Information Technology, Institute for Advanced Studies in Basic Sciences, Zanjan, Iran; 50 Department of Research and Innovation, Petanux Research GmBH, Bonn, Germany; 51 Department of Infectious Disease Epidemiology, London School of Hygiene & Tropical Medicine, London, United Kingdom; 52 University of Genoa, Genoa, Italy; 53 School of Public Health and Health Systems, University of Waterloo, Waterloo, Ontario, Canada; 54 Al Shifa School of Public Health, Al Shifa Trust Eye Hospital, Rawalpindi, Pakistan; 55 Research Unit on Applied Molecular Biosciences (UCIBIO), University of Porto, Porto, Portugal; 56 Department of Microbiology & Infection Control, Medanta Medicity, Gurugram, India; 57 Department of Medicine, University of Toronto, Toronto, Ontario, Canada; 58 Department of Public Health, Texila American University, Georgetown, Guyana; 59 Environmental Health Department, Imam Abdulrahman Bin Faisal University, Dammam, Saudi Arabia; 60 Clinical Dermatology, IRCCS Istituto Ortopedico Galeazzi, University of Milan, Milan, Italy; 61 Department of Dermatology, Case Western Reserve University, Cleveland, Ohio, United States of America; 62 Department of Information Technology, University of Human Development, Sulaymaniyah, Iraq; 63 Toxoplasmosis Research Center, Mazandaran University of Medical Sciences, Sari, Iran; 64 Wellcome Trust Brighton and Sussex Centre for Global Health Research, Brighton and Sussex Medical School, Brighton, United Kingdom; 65 School of Public Health, Addis Ababa University, Addis Ababa, Ethiopia; 66 Department of Community Medicine, University of Peradeniya, Peradeniya, Sri Lanka; 67 Center of Complexity Sciences, National Autonomous University of Mexico, Mexico City, Mexico; 68 Faculty of Veterinary Medicine and Zootechnics, Autonomous University of Sinaloa, Culiacán Rosales, Mexico; 69 Institute of Health Economics and Technology, Hanoi, Vietnam; 70 Reference Laboratory of Egyptian Universities Hospitals, Ministry of Higher Education and Research, Cairo, Egypt; 71 Pediatric Dentistry and Dental Public Health Department, Alexandria University, Alexandria, Egypt; 72 Department of Statistics, Debre Markos University, Debre Markos, Ethiopia; 73 Department of Political Science, University of Human Development, Sulaimaniyah, Iraq; 74 Department of Medical Parasitology, Kerman University of Medical Sciences, Kerman, Iran; 75 Nanotechnology Research Center, Tehran University of Medical Sciences, Tehran, Iran; 76 Department of Pharmaceutical Nanotechnology, Tehran University of Medical Sciences, Tehran, Iran; 77 National Institute for Stroke and Applied Neurosciences, Auckland University of Technology, Auckland, New Zealand; 78 Research Center of Neurology, Moscow, Russia; 79 Associated Laboratory for Green Chemistry (LAQV), University of Porto, Porto, Portugal; 80 Institute of Gerontology, National Academy of Medical Sciences of Ukraine, Kyiv, Ukraine; 81 Department of Medical Parasitology, Abadan Faculty of Medical Sciences, Abadan, Iran; 82 School of Public Health, Medical, and Veterinary Sciences, James Cook University, Douglas, Queensland, Australia; 83 Department of Family and Community Medicine, University Of Sulaimani, Sulaimani, Iraq; 84 Neurology, Public Health and Disability Unit, Carlo Besta Neurological Institute IRCCS (Fondazione IRCCS Istituto Neurologico Carlo Besta), Milan, Italy; 85 Department of Epidemiology and Preventive Medicine, Monash University, Melbourne, Victoria, Australia; 86 Department of Epidemiology, Binzhou Medical University, Yantai City, China; 87 Department of Pharmacology, Tehran University of Medical Sciences, Tehran, Iran; 88 Obesity Research Center, Shahid Beheshti University of Medical Sciences, Tehran, Iran; 89 College of Law and Political Science, University of Human Development, Sulaimaniyah, Iraq; 90 School of Health and Environmental Studies, Hamdan Bin Mohammed Smart University, Dubai, United Arab Emirates; 91 School of Business, London South Bank University, London, United Kingdom; 92 Department of Public Health, Adigrat University, Adigrat, Ethiopia; 93 Department of Biological Sciences, Ohio University, Zanesville, Ontario, United States of America; 94 Department of Pharmacology, Bangladesh Industrial Gases Limited, Tangail, Bangladesh; 95 Institute of Research and Development, Duy Tan University, Da Nang, Vietnam; 96 Department of Computer Science, University of Human Development, Sulaymaniyah, Iraq; 97 College of Science and Engineering, Hamad Bin Khalifa University, Doha, Qatar; 98 Department of Community Medicine, University of Ibadan, Ibadan, Nigeria; 99 Department of Community Medicine, University College Hospital, Ibadan, Ibadan, Nigeria; 100 Department of Epidemiology, University of Kragujevac, Kragujevac, Serbia; 101 College of Public Health, Taipei Medical University, Taipei, Taiwan; 102 Research Institute for Endocrine Sciences, Shahid Beheshti University of Medical Sciences, Tehran, Ira; 103 Department of Community Medicine, Dr. Baba Saheb Ambedkar Medical College & Hospital, Delhi, India; 104 Department of Community Medicine, Banaras Hindu University, Varanasi, India; 105 Gastrointestinal and Liver Diseases Research Center, Guilan University of Medical Sciences, Rasht, Iran; 106 Caspian Digestive Disease Research Center, Guilan University of Medical Sciences, Rasht, Iran; 107 Department of Family Medicine and Public Health, University of Opole, Opole, Poland; 108 School of Public Health, University College Cork, Cork, Ireland; 109 School of Management and Medical Informatics, Tabriz University of Medical Sciences, Tabriz, Iran; 110 Institute for Prevention of Non-communicable Diseases, Qazvin University of Medical Sciences, Qazvin, Iran; 111 Health Services Management Department, Qazvin University of Medical Sciences, Qazvin, Iran; 112 Research Center for Environmental Determinants of Health, Kermanshah University of Medical Sciences, Kermanshah, Iran; 113 Social Determinants of Health Research Center, Tabriz University of Medical Sciences, Tabriz, Iran; 114 Pars Advanced and Minimally Invasive Medical Manners Research Center, Iran University of Medical Sciences, Tehran, Iran; 115 Hematology, Oncology and Stem Cell Transplantation Research Center, Tehran University of Medical Sciences, Tehran, Iran; 116 Department of Applied Physics, The John Paul II Catholic University of Lublin, Lublin Voivodeship, Poland; 117 Department of Biology & Chemistry, Drohobych Ivan Franko State Pedagogical University, Drohobych, Ukraine; 118 International Research Center of Excellence, Institute of Human Virology Nigeria, Abuja, Nigeria; 119 Julius Centre for Health Sciences and Primary Care, Utrecht University, Utrecht, Netherlands; 120 Department of Dermatology, Wolaita Sodo University, Wolaita Sodo, Ethiopia; 121 Mashhad University of Medical Sciences, Mashhad, Iran; 122 Department of Biophysics and Molecular Biology, Baku State University, Baku, Azerbaijan; 123 Institute of Radiation Problems, Azerbaijan National Academy of Sciences, Baku, Azerbaijan; 124 Department of Epidemiology and Biostatistics, Health Services Academy, Islamabad, Pakistan; 125 Department of Population Sciences, Jatiya Kabi Kazi Nazrul Islam University, Mymensingh, Bangladesh; 126 Faculty of Health and Medicine, University of Newcastle, Newcastle, New South Wales, Australia; 127 Faculty of Health and Wellbeing, Sheffield Hallam University, Sheffield, United Kingdom; 128 College of Arts and Sciences, Ohio University, Zanesville, Ohio, United States of America; 129 Department of Medical Parasitology, Cairo University, Cairo, Egypt; 130 Department of Public Health, Kermanshah University of Medical Sciences, Kermanshah, Iran; 131 Wollega University, Nekemte, Ethiopia; 132 School of Traditional Chinese Medicine, Xiamen University Malaysia, Sepang, Malaysia; 133 Independent Consultant, Jakarta, Indonesia; 134 Imperial College Business School, Imperial College London, London, United Kingdom; 135 Faculty of Public Health, University of Indonesia, Depok, Indonesia; 136 Department of Clinical Sciences and Community Health, University of Milan, Milan, Italy; 137 Medical Director, HelpMeSee, New York, New York, United States of America; 138 General Director, Mexican Institute of Ophthalmology, Queretaro, Mexico; 139 School of Nursing, Hong Kong Polytechnic University, Hong Kong, China; 140 School of Public Health and Preventive Medicine, Monash University, Melbourne, Victoria, Australia; 141 Department of Vector Biology, Liverpool School of Tropical Medicine, Liverpool, United Kingdom; 142 Radiology Department, Egypt Ministry of Health and Population, Mansoura, Egypt; 143 Ophthalmology Department, Ministry of Health & Population, Aswan, Egypt; 144 Environmental Health, Tehran University of Medical Sciences, Tehran, Iran; 145 Environmental Health Research Center, Kurdistan University of Medical Sciences, Sanandaj, Iran; 146 Institute for Social Science Research, The University of Queensland, Indooroopilly, Queensland, Australia; 147 Plastic Surgery Department, Iran University of Medical Sciences, Tehran, Iran; 148 School of Medicine, Iran University of Medical Sciences, Tehran, Iran; 149 School of Medicine, University of Manitoba, Winnipeg, Manitoba, Canada; 150 Department of Epidemiology and Biostatistics, Tehran University of Medical Sciences, Tehran, Iran; 151 Campus Caucaia, Federal Institute of Education, Science and Technology of Ceará, Caucaia, Brazil; 152 Department of Twin Research and Genetic Epidemiology, King’s College London, London, United Kingdom; 153 Department of Ophthalmology, Singleton Hospital, Swansea, United Kingdom; 154 Department of Pharmacy, Wollo University, Dessie, Ethiopia; 155 Peru Country Office, United Nations Population Fund (UNFPA), Lima, Peru; 156 Department of Reproductive Health and Population Studies, Bahir Dar University, Bahir Dar, Ethiopia; 157 School of Medicine, Mekelle University, Mekelle, Ethiopia; 158 Department of Environmental Health Sciences and Technology, Jimma University, Jimma, Ethiopia; 159 Clinical Microbiology and Parasitology Unit, Dr. Zora Profozic Polyclinic, Zagreb, Croatia; 160 University Centre Varazdin, University North, Varazdin, Croatia; 161 Pacific Institute for Research & Evaluation, Calverton, Maryland, United States of America; 162 School of Public Health, Curtin University, Perth, Australia; 163 Department of Environmental Health, Sabzevar University of Medical Sciences, Sabzevar, Iran; 164 Non-communicable Diseases Research Center, Sabzevar University of Medical Sciences, Sabzevar, Iran; 165 Biotechnology Research Center, Tabriz University of Medical Sciences, Tabriz, Iran; 166 Molecular Medicine Research Center, Tabriz University of Medical Sciences, Tabriz, Iran; 167 Department of Epidemiology and Biostatistics, Shahrekord University of Medical Sciences, Shahrekord, Iran; 168 Kashmar Center of Higher Health Education, Mashhad University of Medical Sciences, Mashhad, Iran; 169 Health Systems and Policy Research Unit, Ahmadu Bello University, Zaria, Nigeria; 170 Department of Biomolecular Sciences, University of Mississippi, Oxford, Mississippi, United States of America; 171 Department of Pharmacy, Mizan-Tepi University, Mizan, Ethiopia; 172 Department of Epidemiology, Arak University of Medical Sciences, Arak, Iran; 173 Computer, Electrical, and Mathematical Sciences and Engineering Division, King Abdullah University of Science and Technology, Thuwal, Saudi Arabia; 174 Clinical Research Development Center, Kermanshah University of Medical Sciences, Kermanshah, Iran; 175 Research and Analytics Department, Initiative for Financing Health and Human Development, Chennai, India; 176 Department of Research and Analytics, Bioinsilico Technologies, Chennai, India; 177 Comprehensive Cancer Center, University of Alabama at Birmingham, Birmingham, Alabama, United States of America; 178 Department of General Surgery, Carol Davila University of Medicine and Pharmacy, Bucharest, Romania; 179 Department of General Surgery, Emergency Hospital of Bucharest, Bucharest, Romania; 180 Institute for Global Health Innovations, Duy Tan University, Hanoi, Vietnam; 181 Center of Excellence in Behavioral Medicine, Nguyen Tat Thanh University, Ho Chi Minh City, Vietnam; 182 Administrative and Economic Sciences Department, University of Bucharest, Bucharest, Romania; 183 Department of Pathology and Molecular Medicine, McMaster University, Hamilton, Ontario, Canada; 184 Department of Psychiatry and Behavioural Neurosciences, McMaster University, Hamilton, Ontario, Canada; 185 Department of Psychiatry, University of Lagos, Lagos, Nigeria; 186 Diplomacy and Public Relations Department, University of Human Development, Sulaimaniyah, Iraq; 187 Department of Pharmacology and Therapeutics, University of Nigeria Nsukka, Enugu, Nigeria; 188 Department of Health Metrics, Center for Health Outcomes & Evaluation, Bucharest, Romania; 189 Department of Nutrition and Food Sciences, Maragheh University of Medical Sciences, Maragheh, Iran; 190 Dietary Supplements and Probiotic Research Center, Alborz University of Medical Sciences, Karaj, Iran; 191 Thalassemia and Hemoglobinopathy Research Center, Ahvaz Jundishapur University of Medical Sciences, Ahvaz, Iran; 192 Metabolomics and Genomics Research Center, Tehran University of Medical Sciences, Tehran, Iran; 193 Department of Community Medicine, Maharishi Markandeshwar Medical College & Hospital, Solan, India; 194 Kasturba Medical College, Mangalore, Manipal Academy of Higher Education, Manipal, India; 195 Department of Primary Care and Public Health, Imperial College London, London, United Kingdom; 196 Academic Public Health England, Public Health England, London, United Kingdom; 197 WHO Collaborating Centre for Public Health Education and Training, Imperial College London, London, United Kingdom; 198 University College London Hospitals, London, United Kingdom; 199 Department of Computer Science, Boston University, Boston, Massachusetts, United States of America; 200 School of Optometry and Vision Science, University of New South Wales, Sydney, New South Wales, Australia; 201 Brien Holden Vision Institute, Sydney, Australia; 202 Department of Medical Laboratory Science, Woldia University, Woldia, Ethiopia; 203 Department of Medical Microbiology, University of Pretoria, Pretoria, South Africa; 204 Center for Research in Congenital Anomalies and Rare Diseases, ICESI University (Centro de Investigaciones en Anomalías Congénitas y Enfermedades Raras, Universidad Icesi), Cali, Colombia; 205 Department of Biomedical Sciences, University of Sassari, Sassari, Italy; 206 Public Health, Ministry of Health and Medical Education, Qom, Iran; 207 Qom University of Medical Sciences, Qom, Iran; 208 Department of Phytochemistry, Soran University, Soran, Iraq; 209 Department of Nutrition, Cihan University, Erbil, Iraq; 210 Department of Entomology, Ain Shams University, Cairo, Egypt; 211 Department of Health and Society, Faculty of Medicine, University of Applied and Environmental Sciences, Bogota, Colombia; 212 National School of Public Health, Carlos III Health Institute, Madrid, Spain; 213 Department of Public Health Sciences, University of North Carolina at Charlotte, Charlotte, North Carolina, United States of America; 214 Public Health Division, An-Najah National University, Nablus, Palestine; 215 Independent Consultant, Karachi, Pakistan; 216 Faculty of Caring Science, Work Life, and Social Welfare, Faculty of Caring Science, Work Life and Social Welfare, University of Borås, Borås, Sweden, Borås, Sweden; 217 Centre for Medical Informatics, University of Edinburgh, Edinburgh, United Kingdom; 218 Division of General Internal Medicine, Harvard University, Boston, Massachusetts, United States of America; 219 Department of Forensic Medicine and Toxicology, Manipal Academy of Higher Education, Mangalore, India; 220 College of Medicine, Yonsei University, Seoul, South Korea; 221 Public Health Dentistry Department, Krishna Institute of Medical Sciences Deemed to be University, Karad, India; 222 Department of Law, Economics, Management and Quantitative Methods, University of Sannio, Benevento, Italy; 223 WSB University in Gdańsk, Gdansk, Poland; 224 School of Medicine, University of Alabama at Birmingham, Birmingham, Alabama, United States of America; 225 Medicine Service, US Department of Veterans Affairs (VA), Birmingham, Alabama, United States of America; 226 Department of Ophthalmology, Hywel Dda University Health Board, Llanelli, United Kingdom; 227 Nursing Care Research Center, Semnan University of Medical Sciences, Semnan, Iran; 228 Department of Community Medicine, Ahmadu Bello University, Zaria, Nigeria; 229 Cancer Control Center, Osaka International Cancer Institute, Osaka, Japan; 230 Department of Biostatistics, Hamadan University of Medical Sciences, Hamadan, Iran; 231 Non-communicable Diseases Research Center, Hamadan University of Medical Sciences, Hamadan, Iran; 232 Department of Global Health Research, Adaptive Knowledge Management, Victoria, British Columbia, Canada; 233 Institute for Risk Assessment Sciences (IRAS), Utrecht University, Utrecht, Netherlands; 234 Department of Health Economics, Hanoi Medical University, Hanoi, Vietnam; 235 Department of Allied Health Sciences, Iqra National University, Peshawar, Pakistan; 236 Department of Medical Microbiology/Parasitology, Ebonyi State University, Abakaliki, Nigeria; 237 Kasturba Medical College, Manipal Academy of Higher Education, Mangalore, India; 238 Division of Health Sciences, University of Warwick, Coventry, United Kingdom; 239 Control of Infectious Diseases, Erasmus University Medical Center, Rotterdam, Netherlands; 240 Department of Medical and Surgical Sciences, University of Bologna, Bologna, Italy; 241 Occupational Health Unit, Sant’Orsola Malpighi Hospital, Bologna, Italy; 242 Department of Public Health, Debre Markos University, Debre Markos, Ethiopia; 243 Department of Diabetes and Metabolic Diseases, University of Tokyo, Tokyo, Japan; 244 School of International Development and Global Studies, University of Ottawa, Ottawa, Ontario, Canada; 245 The George Institute for Global Health, University of Oxford, Oxford, United Kingdom; 246 Health Services Management Research Center, Kerman University of Medical Sciences, Kerman, Iran; 247 Department of Health Management, Policy, and Economics, Kerman University of Medical Sciences, Kerman, Iran; 248 Centre for Suicide Research and Prevention, University of Hong Kong, Hong Kong, China; 249 Department of Social Work and Social Administration, University of Hong Kong, Hong Kong, China; 250 Department of Neuropsychopharmacology, National Center of Neurology and Psychiatry, Kodaira, Japan; 251 Department of Public Health, Juntendo University, Tokyo, Japan; 252 Department of Epidemiology and Biostatistics, Wuhan University, Wuhan, China; 253 School of Public Health and Management, Hubei University of Medicine, Shiyan, China; 254 Department of Health care Management and Economics, Urmia University of Medical Science, Urmia, Iran; 255 Department of Parasitology and Entomology, Tarbiat Modares University, Tehran, Iran; 256 The School of Clinical Sciences at Monash Health, Monash University, Melbourne, Victoria, Australia; 257 Maternal and Child Health Division, International Centre for Diarrhoeal Disease Research, Bangladesh, Dhaka, Bangladesh; 258 School of Medicine, Wuhan University, Wuhan, China; 259 School of Public Health, Wuhan University of Science and Technology, Wuhan, China; 260 Hubei Province Key Laboratory of Occupational Hazard Identification and Control, Wuhan University of Science and Technology, Wuhan, China; 261 Department of Health Education and Health Promotion, Kermanshah University of Medical Sciences, Kermanshah, Iran; University of Buea, CAMEROON

## Abstract

Recent evidence suggests that, in some foci, elimination of onchocerciasis from Africa may be feasible with mass drug administration (MDA) of ivermectin. To achieve continental elimination of transmission, mapping surveys will need to be conducted across all implementation units (IUs) for which endemicity status is currently unknown. Using boosted regression tree models with optimised hyperparameter selection, we estimated environmental suitability for onchocerciasis at the 5 × 5-km resolution across Africa. In order to classify IUs that include locations that are environmentally suitable, we used receiver operating characteristic (ROC) analysis to identify an optimal threshold for suitability concordant with locations where onchocerciasis has been previously detected. This threshold value was then used to classify IUs (more suitable or less suitable) based on the location within the IU with the largest mean prediction. Mean estimates of environmental suitability suggest large areas across West and Central Africa, as well as focal areas of East Africa, are suitable for onchocerciasis transmission, consistent with the presence of current control and elimination of transmission efforts. The ROC analysis identified a mean environmental suitability index of 0·71 as a threshold to classify based on the location with the largest mean prediction within the IU. Of the IUs considered for mapping surveys, 50·2% exceed this threshold for suitability in at least one 5 × 5-km location. The formidable scale of data collection required to map onchocerciasis endemicity across the African continent presents an opportunity to use spatial data to identify areas likely to be suitable for onchocerciasis transmission. National onchocerciasis elimination programmes may wish to consider prioritising these IUs for mapping surveys as human resources, laboratory capacity, and programmatic schedules may constrain survey implementation, and possibly delaying MDA initiation in areas that would ultimately qualify.

## Author summary

As of 2018, it was unknown if onchocerciasis transmission occurred among approximately 2 400 implementation units (IUs; typically, second administrative-level units, such as districts) considered potentially endemic. These IUs have either never been surveyed for onchocerciasis or historical data are not sufficient to define contemporary endemicity status. Given the large number of IUs for which baseline data collection is likely required to achieve continental elimination, there is a need to prioritise areas for surveys to ensure that those suitable for endemic transmission, and therefore potentially eligible for mass drug administration, are able to initiate interventions as soon as possible. We used boosted regression trees to predict environmental suitability for onchocerciasis, with corresponding measures of uncertainty. We summarized the fine scale spatial predictions at the IU level by using receiver operating characteristic (ROC) curve analysis to identify a threshold that maximized agreement with the occurrence locations to identify IUs that may warrant prioritisation for mapping surveys. This analysis suggests that approximately half of the IUs considered for surveys could be classified as environmentally suitable for onchocerciasis. In order to develop an elimination strategy, many national onchocerciasis elimination programmes (NOEPs) need a mechanism to synthesise historical data to define priority areas for surveys.

## Introduction

Onchocerciasis (a disease caused by infection with *Onchocerca volvulus*) can lead to permanent blindness and skin disease, and over 99% of people infected reside in Africa [1]. Since the mid-1970s, vector control of the *Simulium* black fly vectors and, since the late-1980s, mass drug administration (MDA) with ivermectin, have been implemented (in combination or using MDA alone) with the goal of reducing onchocerciasis-related morbidity in areas of meso- to hyper-endemicity [2]. To date, over one billion ivermectin treatments have been administered by national onchocerciasis control programmes, in addition to millions of ivermectin treatments provided for the elimination of lymphatic filariasis (LF) as a public health problem [2]. Preventive chemotherapy with MDA (in which individuals residing in endemic areas are offered ivermectin) has been identified as the primary intervention for the control of onchocerciasis-related morbidity and elimination of onchocerciasis transmission [2]. Under the former Onchocerciasis Control Programme (OCP) in West Africa and the African Programme for Onchocerciasis Control (APOC), as well as onchocerciasis-control programmes supported by other partners, areas eligible for MDA were often identified by purposively sampling communities near known or suspected *Simulium* breeding sites. Prevalence of onchocerciasis was estimated via skin snip biopsy to detect the presence of microfilariae under standardised protocols (for OCP) or nodule palpation (onchocercoma), the latter leading to the rapid epidemiological mapping for onchocerciasis (REMO) tool [3]. This approach was generally successful at identifying foci with moderate to high levels of transmission (nodule prevalence ≥20%) [4], but is less sensitive in low-prevalence settings [5].

In 2012, the paradigm for onchocerciasis programmes began to shift from control to elimination [6]. Recent evidence from the Americas [7,8] and Africa [9,10] has shown that annual or semi-annual MDA reaching at least 80% of the eligible population may halt transmission after a period of variable duration (in part determined by baseline endemicity) [11], achieving local elimination in some foci [12]. To ultimately achieve elimination of transmission, therefore, endemic areas must first be correctly delineated to ensure timely initiation of interventions [13]. Various stakeholders are now exploring the feasibility of eliminating onchocerciasis across Africa [14], and several methods are under consideration to identify areas eligible for MDA. As of 2018, the Expanded Special Project for Elimination of Neglected Tropical Diseases (ESPEN) at the World Health Organization Africa Region (WHO-AFRO) identified approximately 2 400 implementation units (IUs), typically second administrative-level units (such as districts), for which endemicity status is uncertain due to a lack of current prevalence data. Of these, 1 651 IUs have never received ivermectin MDA, and 783 IUs currently receive ivermectin (plus albendazole) as part of LF programmes, or may be under post-MDA surveillance for LF [15]. The objective of this analysis was to predict to what extent these IUs of uncertain endemicity status were likely to be environmentally suitable for onchocerciasis. The results of this analysis could be used by national programmes and implementing partners to identify priority areas for mapping surveys.

## Methods

### Data inputs

We first constructed an analytical dataset of locations at which onchocerciasis has been detected (‘occurrences’). The case definition of an occurrence included any geo-referenced data point or polygon (i.e., areal data) for which at least one person tested positive using any of the following diagnostics to measure prevalence of onchocerciasis infection or onchocerciasis-related disease: nodule palpation; skin snip microscopy; onchocerciasis-related eye or skin disease; or Ov16 seropositivity, as well as other diagnostic tests (see S1 Text). Two alternative case definitions were tested in a sensitivity analysis, described in S1 Text. Inputs were obtained from a systematic literature review of the prevalence of human onchocerciasis and onchocerciasis-related morbidity published from 1975 to 2017, detailed elsewhere [16]. Since the majority of onchocerciasis prevalence data were reported by national onchocerciasis control and elimination programs for the purposes of programme monitoring, we also extracted prevalence data from the ESPEN [15] online portal. We further requested prevalence data collected under the OCP–operational in West Africa from 1974 to 2002 –from its former Director, BA Boatin, PhD (personal communication, January 2019). Locations missing geographical information (i.e., latitude-longitude for community-level prevalence or a shapefile for areal data) were geo-referenced following the procedures described in Hill et al.[16] Further details on the dataset are presented in S1 Text.

For the boosted regression tree (BRT) [17] model to compare against a set of control conditions, we must also provide it with examples of environmental conditions where onchocerciasis has not been detected. Since methods used to detect onchocerciasis included skin biopsy and nodule palpation, it is possible that reports of zero prevalence may not be true absences, particularly among areas of low prevalence, due to low sensitivity of these methods [18]. Rather than use reported absence data (which may include false negatives), we therefore randomly sampled background data to provide a contrast signal, re-implementing protocols from prior ecological and epidemiological species distribution analyses akin to pseudo-absence data [19]. Background points were sampled independently across 100 bootstraps and uniformly from within 100 km of the input data locations (polygon boundaries and point locations) such that the number of samples from each region (defined as a 100-km buffer from an occurrence location) matched the number of occurrence records associated with it. Since the 100-km regions would overlap with the IUs for which endemicity status was known, we did not sample from IUs considered endemic for onchocerciasis, and avoided sampling within polygonal locations in the occurrence dataset. We used a shapefile provided by ESPEN to identify IUs to conduct background sampling (S1 Text).

### Covariates

Ten covariates were included in the analysis based on known evidence of an association with presence of the vector. These included variables that represent climatic factors (aridity, precipitation, and daytime temperature), vegetation (enhanced vegetation index, tasseled cap brightness, and tasseled cap wetness), breeding sites near fast-flowing rivers (distance to rivers, slope, and elevation) and transmission occurring in rural areas (urbanicity). Details regarding covariate selection, source information, and visualisations are included in S2 Text and S4 Table.

### Statistical analysis

Since the purpose of the analysis was to predict environmental suitability of onchocerciasis among countries for which onchocerciasis endemicity was uncertain, we excluded all IUs from countries considered entirely non-endemic (as reported by ESPEN): Algeria, Botswana, Cabo Verde, Eritrea, Eswatini, the Gambia, Lesotho, Madagascar, Mauritius, Mauritania, Seychelles, South Africa, and Zimbabwe (see S1 Text). The rationale for this exclusion was to avoid selecting locations in the background sample that would introduce extreme covariate values into the analysis (such as the Sahara desert). To predict the environmental suitability of onchocerciasis, we employed 100 optimised BRT models [17] to produce a final, bootstrap aggregated BRT model. The BRT method models environmental suitability of onchocerciasis transmission as a suitability index (from 0 to 1) based on the values of environmental covariates at the locations corresponding to occurrence inputs. We first employed Bayesian parameter optimisation [17,20–22] to select values for three hyperparameters needed to implement the BRT method: the number of leaves of each learned tree (tree complexity), the weighting assigned to previously learned models (learning rate), and number of trees. Additional details on hyperparameter selection and the BRT methodology are presented S3 Text. Once final hyperparameter values were selected, we then implemented 100 BRT models to generate the mean, lower 2·5^th^ percentile, and 97·5^th^ percentile predictions for every 5 × 5-km location. We used covariate values from the year 2016 to generate predictions across the entire geographical extent of the analysis.

The final models for the environmental suitability of onchocerciasis were evaluated based on the average root mean square error (RMSE) and area under the receiver operating curve (AUC) of each bootstrap and the relative influence and marginal effect curve of each covariate. This allowed us to estimate the significance within the model of each environmental factor and its behaviour relative to all other covariates and to the covariate values associated with the input data.

We comply with the Guidelines for Accurate and Transparent Health Estimates Reporting (GATHER) [23] as outlined in the S1 Table. Complete information on data sources is available from the Global Health Data Exchange. Related statistical code for R 3·1·2 is available at GitHub. Maps were produced using ArcGIS Desktop 10.6.

### Thresholding and summarisation by implementation unit

We used the existing data set to identify an optimised threshold value to classify the modelled environmental suitability index into a binary presence/absence classification [24]. The threshold selected was the value that minimised the classification error associated with the model, and therefore most appropriately indicated reported occurrences to be in locations predicted to be environmentally suitable, and sampled background inputs to be less suitable. Using the receiver operating characteristic (ROC) curve, we evaluated the sensitivity and specificity trade-off possible values from 0 to 1, finding the value which minimised the distance to (0, 1) on the ROC plot. Since an IU (or area within an IU) would generally qualify for MDA if any location were identified to have evidence of onchocerciasis transmission through primary data collection, we summarised each IU as a function of this binary classification based on the value of the 5 × 5-km location with the largest mean prediction within each IU. We then estimated the posterior probability that a single IU would include any location that exceeded the threshold identified. We compared these with the reported endemicity status available in the shapefile used to conduct the background sample. Addition details are presented in the S9 Fig.

## Results

**Fig 1:**
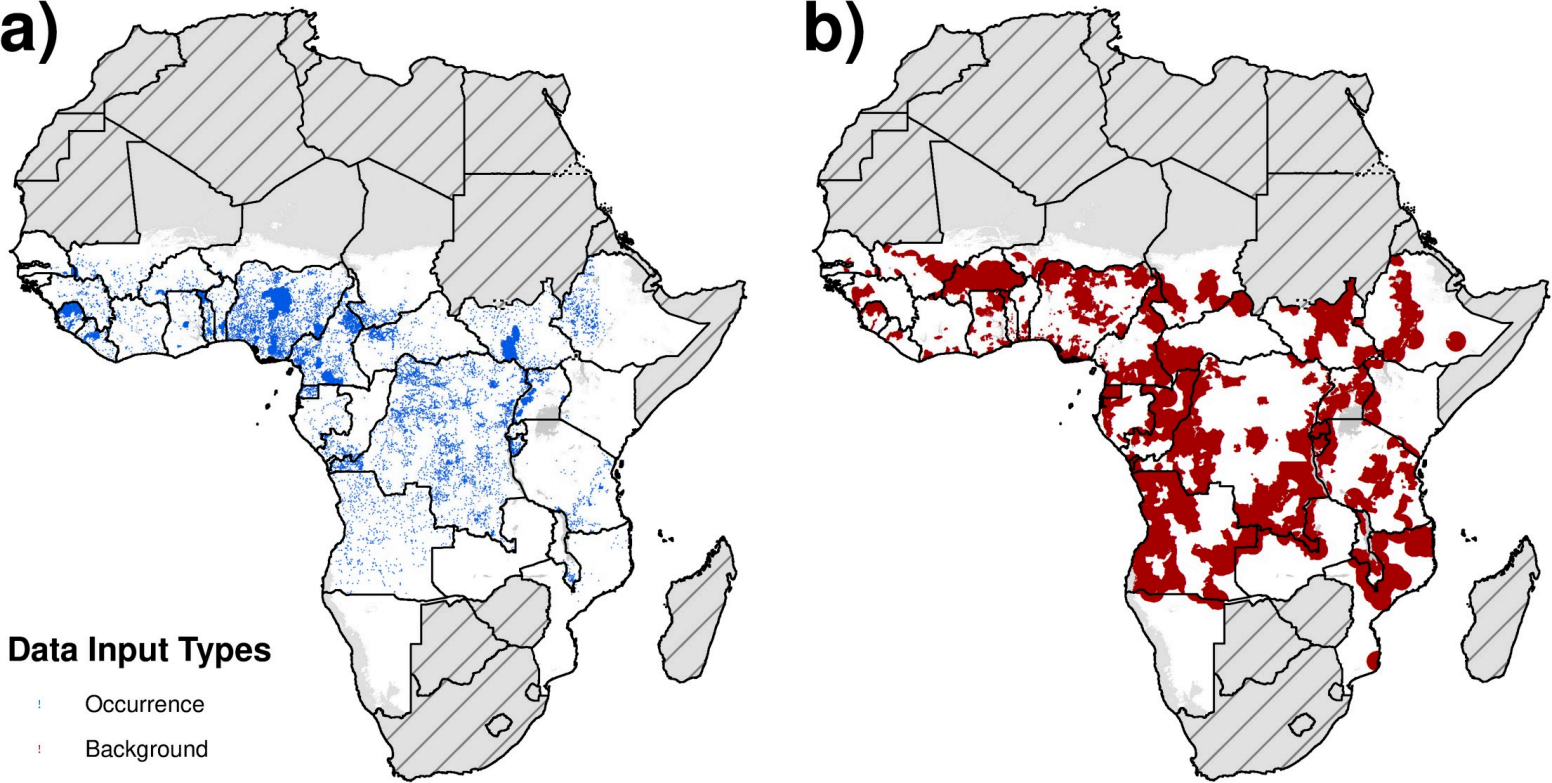
**Location of data sources:** (a) Location of occurrence data points are visualised in blue. (b) Locations chosen for the background sample are mapped in red. The background sample represents the locations chosen to compare against the occurrence data points. IUs for which endemicity status was uncertain and mapping surveys are considered were excluded from selection. Due to the density of background points chosen, they appear as polygon data in the map. Countries in grey with hatch marks were excluded from the analysis based on a review of national endemicity status. Areas in grey only represent locations masked due to sparse population. Maps were produced using ArcGIS Desktop 10.6 and shapefiles to visualize administrative units are available at https://espen.afro.who.int/tools-resources/cartography-database.

The final dataset contained 13 382 occurrence records: 11 094 from ESPEN; 689 non-ESPEN data points (from BK Mayala, PhD at the Demographic and Health Surveys (DHS) Program, personal communication); 863 from the systematic review; and 736 from OCP historical records. Of all these records, 128 were georeferenced as polygons. A summary of the original data extracted by diagnostic used, year, and country is presented in S1 Fig and S2 and S3 Tables. After de-duplication across all sources, a total of 987 records reported skin snip examination, 12 161 nodule palpation, 155 onchocerciasis-related skin or eye disease, and 98 reported serological antibody testing (Ov16 ELISA or rapid diagnostic test, RDT). Fig 1 presents a map of locations of the occurrence and background sample. Overall, the Democratic Republic of the Congo (DRC) and Nigeria reported what amounted to be 50% of occurrence locations (3 933 locations (29%) in the DRC; 2 755 locations (21%) in Nigeria).

### Results at the 5 × 5-km resolution

**Fig 2:**
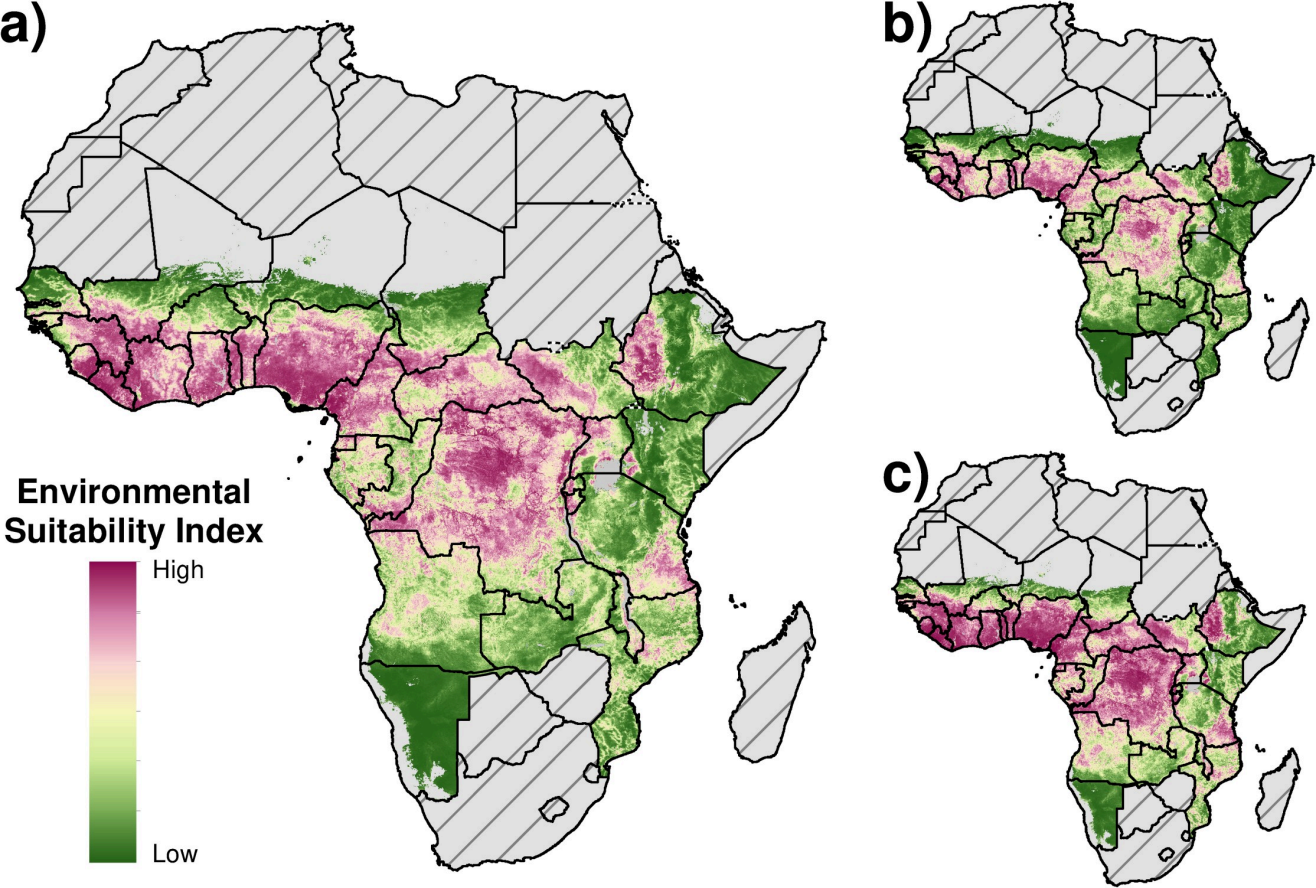
**Environmental suitability predictions:** Visualisation of (a) mean, (b) lower 95% uncertainty interval, and (c) upper 95% uncertainty interval. Environmental suitability index predicted by the model is bounded from 0% (low) to 100% (high). Countries in grey were excluded from the analysis. Countries in grey with hatch marks were excluded from the analysis based on a review of national endemicity status. Areas in grey only represent locations masked due to sparse population. Maps were produced using ArcGIS Desktop 10.6 and shapefiles to visualize administrative units are available at https://espen.afro.who.int/tools-resources/cartography-database.

The results of the environmental suitability model for Africa are presented in Fig 2. The model results show higher environmental suitability across most of the southern half of West Africa, the DRC, South Sudan, and western Ethiopia, as well as in areas of Tanzania and Mozambique. The highest 5 × 5-km grid-cell-level predictions were observed in Equatorial Guinea, Nigeria, the DRC, and Cameroon (>0·98). The mean predictions show areas suitable for transmission that generally agree with previous model-based geostatistical analyses [25,26]. In West Africa, our model predictions suggest high suitability in areas compared to a previous model [25] predicting lower endemicity in Liberia, northwest Ghana, and northern Guinea. In other regions, we predict high suitability in the area bordering the Republic of the Congo, the DRC, and Angola, as well as in eastern and southern Malawi, northern Nigeria, western Kenya, and eastern Central African Republic; these areas were predicted to have low prevalence in prior estimates [26]. Model fit statistics for AUC were 0·90 and RMSE was 0·38. The marginal effects of the covariates were highest for aridity (0·22), precipitation (0·15), and elevation (0·16). Covariate influence plots, and results of the sensitivity analyses are included in S3−S9 Figs. Model results are available via https://vizhub.healthdata.org/lbd/oncho and country-level map results are included in S1 Appendix.

### Results at the IU level

**Fig 3:**
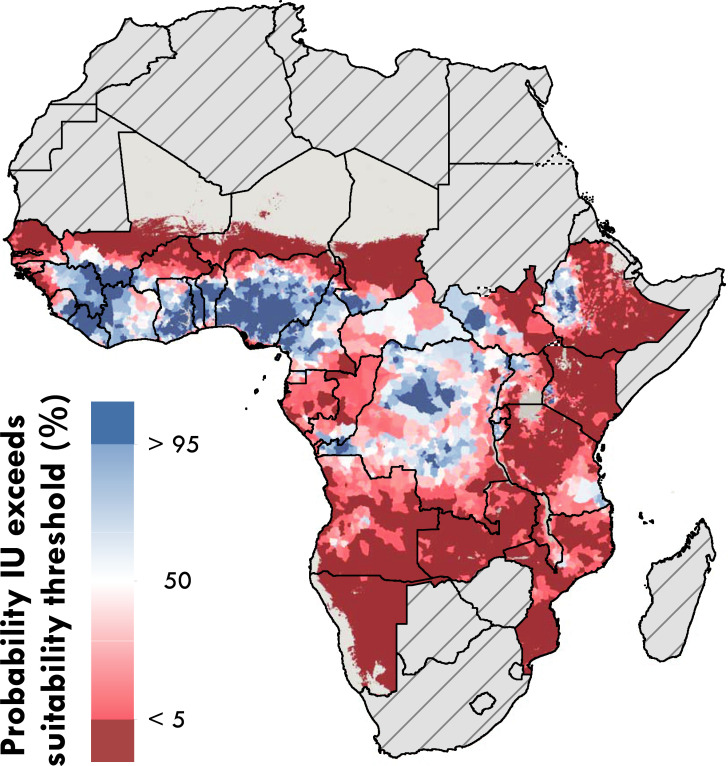
Posterior probability any location with Implementation Units (IU) exceeds the threshold for suitability. The posterior probability (%) of an IU including a location that exceeds the 0·71 threshold used to identify areas of suitability is estimated from the 100 BRT bootstraps. Areas in red are less likely to have at least one location defined as suitable, areas in blue are more likley to include environmentally suitable locations. Countries in grey with hatch marks were excluded from the analysis based on a review of national endemicity status. Areas in grey only represent locations masked due to sparse population. Maps were produced using ArcGIS Desktop 10.6 and shapefiles to visualize administrative units are available at https://espen.afro.who.int/tools-resources/cartography-database.

The ROC analysis identified a mean prediction of ≥0·71, best agreed with the location of occurrences, and was used to classify IUs as environmentally suitable. We identified a total 3 087 IUs that include at least one location for which the mean model results suggest environmental suitability for onchocerciasis transmission. We summarise this across the four types of IU endemicity status (as reported at the time of this analysis) in Table 1. Overall, the environmental suitability predictions are concordant with areas previously identified as endemic or non-endemic when classifying based on the maximum mean grid-cell-level prediction within the boundaries of an IU. Of the IUs considered for elimination mapping, a total of 828 IUs (50·2%) had environmental suitability predictions that exceeded the 0·71 threshold in at least one grid cell. The majority of IUs with high environmental suitability are located in Angola (81 IUs), the DRC (191 IUs), Ethiopia (94 IUs), Kenya (89 IUs), and Nigeria (79 IUs). Among IUs currently under MDA with ivermectin for the purpose of LF elimination, 495 (63%) were predicted to have at least one grid cell exceeding 0·71. In Fig 3, we present the posterior probability that a location within IUs exceeds the threshold, incorporating uncertainty into the classification of ‘suitable’.

**Table 1:** Comparison of implementation unit (IU) classification using reported endemicity versus modelled environmental suitability model.

Endemicity status	Total IUs	Total (%) classified as suitable*
Non-endemic	634	89 (14%)
Endemic (current and historic)	1 710	1 672 (89%)
Considered for elimination mapping	1 651	828 (50%)
Uncertain (under MDA for LF)	783	498 (63%)

*IUs are classified as suitable based on the model results if any location within the IU exceeded the threshold of 0·71.

### Within-IU differences in environmental suitability

While programmatic decisions typically occur at the IU level, we summarised the range of predictions to determine if any IUs identified as suitable were the result of smaller areas of high predicted suitability, as this would suggest IUs for which there may be foci of transmission as opposed to potential transmission across the entire IU. Among IUs that exceeded the 0·71 threshold, the range of predictions within IU borders was as large as 0·95 (suggesting a high degree of variation within the IU). Only 20% of the IUs identified as suitable had mean predictions that exceeded the threshold across all locations within IU boundaries. The range of mean suitability is presented S3 and S5 Figs.

## Discussion

The environmental suitability model identified approximately half of the IUs currently considered for elimination mapping surveys as environmentally similar to areas for which onchocerciasis has been previously detected. These results suggest that a large proportion of IUs considered for mapping might be of lower priority for survey implementation, particularly for programmes in countries such as Ethiopia that may need to survey as many as 461 IUs. Using the results of this analysis, the national program in Ethiopia could prioritise data collection for the 94 IUs that exceeded the threshold. While not the primary target of inference, these results also suggest that over half of the IUs currently under ivermectin MDA for the purposes of elimination of LF as a public health problem may also be suitable for onchocerciasis. Given the goal of elimination of transmission, the duration of MDA required for onchocerciasis is longer than what is implemented for LF. Surveys of onchocerciasis prevalence would be warranted in these IUs to determine if MDA with ivermectin should continue, or else risk the reductions in onchocerciasis prevalence achieved through LF program interventions may be lost owing to interruption of MDA.

While the model results do not completely rule out the need to conduct mapping surveys in areas predicted to have less suitability, they may enable prioritisation of survey implementation planning, assuming some constraints on survey effort in space and time. Standard guidelines for determining IU eligibility for MDA are currently under development, with methods such as purposive sampling of villages based on proximity to breeding sites (‘first-line villages’) and IU-level cluster random surveys under consideration. Regardless of the mapping strategy chosen to identify IUs warranting MDA, NOEPs will likely need information to prioritise IUs for implementation of surveys. Prioritisation of data collection activities, such as considering environmental suitability along with other factors such as proximity to endemic districts and presence of existing programme infrastructure, could result in more efficient resource deployment, particularly given the costs of fieldwork, seasonal constraints such as weather and other health system activities, as well as demands on lab capacity should confirmation of Ov16 RDT results with ELISA be required. Ultimately, prioritisation of IUs likely to be endemic would also enable more rapid scale-up of MDA (or other strategies such as ‘test-and-treat’ in areas co-endemic for *Loa loa* filariasis); once evidence of onchocerciasis transmission is available, donated ivermectin can be deployed. We recommend national programs compare these results with data on *Loa loa* prevalence to inform intervention strategies in co-endemic settings.

Environmental suitability predictions, when overlaid with maps or satellite imagery of settlements, may also be useful to NOEPs for other applications aside from IU-level decision making. They may wish to consider use of these results to identify areas for sampling within IUs, especially those with no first-line village or breeding site information. Among the IUs for which our estimates suggest environmental suitability is high, in settings such as the DRC, Malawi, and Nigeria, predictions were variable within individual IUs, suggesting that differences within IUs may require consideration during baseline survey site selection or could inform sampling strategies. Once mapping for onchocerciasis in these areas is conducted, newly collected data can be used to validate this model’s performance (akin to a natural hold-out) and then subsequently included in future updates to improve the quality of predictions.

### Strengths

With over 13 000 occurrence point inputs, we exceed the number of inputs used for other global analyses of environmental suitability for disease transmission (e.g., dengue [27] and leishmaniasis [28]). The method employed to select values for the BRT hyperparameters avoids the computational demands of implementing a deterministic grid search and is superior to a random search, combining faster computation with iterative selection of the best hyperparameter values [29]. Conceptually, the choice to employ a background sample rather than observed absence data is analogous to certain case-control designs, where the controls are selected to represent the exposure distribution among the source population that gave rise to the cases. In this context, the background sample represents covariate patterns that describe onchocerciasis-endemic countries generally and allows the model to test for associations between covariate patterns identified across all occurrence locations within that setting. Further, since the background sample replaces the observed absence data (where no individuals test positive at a given location), we avoid bias from diagnostic procedures that have poor sensitivity and specificity, such as nodule palpation. It is possible that some observed absences measured by nodule palpation were false negatives [30] or positives. By comparing the IUs known to be currently or historically endemic and those known to be non-endemic with the results of this analysis, we showed strong agreement, suggesting that the model has accurately characterised IUs for which knowledge of endemicity status exists. Finally, this analysis transforms detailed 5 × 5-km-level predictions into IU-level results, which is the unit of programmatic decision making.

### Limitations

The primary limitation of this analysis is that we predict mean values of environmental suitability as an index, a measure not directly comparable to other quantities. This analysis can only indicate how similar a location may be (relative to the covariates included in the analysis) to other locations where onchocerciasis has previously been detected; it does not predict the magnitude of infection. We define suitable regions to be any area with a mean estimate exceeding the optimal threshold. In this way, IUs with ‘high suitability’ are defined relative to each bootstrap and not relative to one another. Other thresholds could be used to aggregate these results to characterise individual IUs. Second, it is possible that covariate patterns identified as suitable for onchocerciasis transmission are biased towards IUs of higher prevalence, as data collection for onchocerciasis control prioritised identification of areas with greater morbidity, generally associated with higher levels of infection prevalence. It is also possible the model will predict high environmental suitability among locations similar to onchocerciasis-endemic settings even if the location lacks the vector or the parasite, or among settings where transmission has been eliminated. We encourage NOEPs to consider the model results alongside programmatic evidence. Third, it is also possible that the covariate patterns at the 5 × 5-km resolution do not adequately capture the specific ecological niche for *Simulium* breeding sites in all settings, and the vector can travel beyond the 5 km range [31]. *Simulium* abundance data is not available for the entire African continent nor is it available per unique species; we therefore rely on other covariates as proxies to represent ecological conditions that might be suitable to the vector. In some settings, smaller rivers may serve as viable breeding sites and future analysis should consider more detailed hydrological data sources. Matching covariate values for temperature, precipitation, enhanced vegetation index, urbanicity, tasseled cap wetness, and tasseled cap brightness to input data by year of data collection was not possible for occurrence data pre-2000 (approximately 20% of the inputs), which may also introduce bias for areas where substantial changes have occurred, although use of annual mean values would be less sensitive to seasonal variation from year to year. We are unable to account for temporal shifts in river locations, but note that calculating distance to rivers at the 5x5km spatial scale likely reduces the potential error. Remote sensing methods have been used to generate a spectral signal to identify potential breeding sites at a much finer spatial scale [32] (0.6m^2^), but it would be computationally infeasible to use those inputs for a continental analysis. Fourth, there may be potential differences in the ecological niche of onchocerciasis in West Africa compared to Central or East Africa driven by forest or savannah habitats [33]. Due to the limited data available for West Africa (beyond Nigeria), conducting separate sub-continental analyses was not feasible, particularly for the former OCP areas. Finally, BRT models are highly sensitive to the selection of inputs; results may vary by the case definition of an occurrence. Our sensitivity analysis (see S3−S6 Figs) did not result in qualitatively different results in mean predictions. We further did not exclude occurrence inputs reporting Ov16 seropositivity, which may not represent contemporary transmission in cases where only one or two individuals test positive. Inclusion of nodule palpation data could also be subject to bias in areas of low endemicity [30]. Less than 1% of the input data reported using serological tests and exclusion of these data from preliminary models resulted in negligible differences in the results (results not shown). It was also not possible to review the original source documentation of data reported via the ESPEN portal or historical data from OCP; bias may have been introduced if those sources contain inaccuracies. There were also not sufficient entomological monitoring data available to compare against all human prevalence data to include evidence of transmission in the vector population as part of our case definition of an occurrence. The background sample cannot account for possible bias in the occurrence data which may have been selected preferentially with respect to locations suspected or known to be endemic. For this reason, we do not recommend the model results be used to exclude any location from mapping to determine program eligibility for MDA or other interventions, but rather to use the model as a tool for prioritization and comparison alongside other data sources.

There are additional complexities that programmes should consider if these model results are used for planning. In settings where population movement due to factors such as conflict, instability, or seasonal migration results in transmission of infection occurring at locations distant from settlements, the results of survey mapping and the model estimates may be discordant. It will be important for NOEPs to characterise such communities, particularly if diagnostics that detect Ov16 seropositivity are used in adult populations, as individuals may test positive if they have been exposed to onchocerciasis earlier in life at other locations. Since the model can only identify locations for which covariate patterns are similar to areas for which onchocerciasis has previously been detected, we would encourage NOEPsto interpret these model results as a mechanism to prioritise surveys, not as a substitute for primary data collection.

### Conclusions

The shift from morbidity control in meso- to hyper-endemic areas to eliminating [13] transmission at the pan-African scale provides a unique opportunity to develop and validate a model to help NOEPs identify endemic IUs with greater efficiency. Our analysis expands upon prior work [25,26] to incorporate more data sources, generate predictions for the entire set of countries suspected or known to be onchocerciasis-endemic (not only areas defined by regional control programmes), and translates detailed spatial predictions into IU-level results for use in program decision making. The large scale of data collection required throughout the African continent to achieve elimination of transmission can benefit from modelled estimates of environmental suitability to facilitate programme planning. If IUs most likely to sustain transmission of onchocerciasis can be surveyed earlier, this may result in faster MDA initiation in those areas. Evidence from settings where local elimination of transmission has already been achieved suggests that at least 10 to 15 years of high coverage MDA may be required under annual or twice-yearly treatment [11,12,34]. To reach elimination of onchocerciasis transmission, it is imperative that districts in need of MDA are identified as quickly as possible.

## Supporting information

S1 Fig Data coverage by year. Here we visualise the volume of data used in the analysis by country and year. Larger circles indicate more data inputs. ‘NA’ indicates records for which no year was reported (eg, ‘pre-2000’).
(JPG)

S2 Fig Illustration of covariate values for year 2000. Maps were produced using ArcGIS Desktop 10.6.
(JPG)

S3 Fig Environmental suitability of onchocerciasis including locations that have received MDA for which no pre-intervention data are available. This plot shows suitability predictions from green (low = 0%) to pink (high = 100%), representing those areas where environmental conditions are most similar to prior pathogen detections. Countries in grey with hatch marks were excluded from the analysis based on a review of national endemicity status. Areas in grey only represent locations masked due to sparse population. Maps were produced using ArcGIS Desktop 10.6 and shapefiles to visualize administrative units are available at https://espen.afro.who.int/tools-resources/cartography-database.
(JPG)

S4 Fig Environmental suitability prediction uncertainty including locations that have received MDA for which no pre-intervention data are available. This plot shows uncertainty associated with environmental suitability predictions colored from blue to red (least to most uncertain). Countries in grey with hatch marks were excluded from the analysis based on a review of national endemicity status. Areas in grey only represent locations masked due to sparse population. Maps were produced using ArcGIS Desktop 10.6 and shapefiles to visualize administrative units are available at https://espen.afro.who.int/tools-resources/cartography-database.
(JPG)

S5 Fig Environmental suitability of onchocerciasis excluding morbidity data. This plot shows suitability predictions from green (low = 0%) to pink (high = 100%), representing those areas where environmental conditions are most similar to prior pathogen detections. Countries in grey with hatch marks were excluded from the analysis based on a review of national endemicity status. Areas in grey only represent locations masked due to sparse population. Maps were produced using ArcGIS Desktop 10.6 and shapefiles to visualize administrative units are available at https://espen.afro.who.int/tools-resources/cartography-database.
(JPG)

S6 Fig Environmental suitability prediction uncertainty excluding morbidity data. This plot shows uncertainty associated with environmental suitability predictions colored from blue to red (least to most uncertain). Countries in grey with hatch marks were excluded from the analysis based on a review of national endemicity status. Areas in grey only represent locations masked due to sparse population.
(JPG)

S7 Fig Covariate Effect Curves for all onchocerciasis occurrences (measures of infection prevalence and disability). On the right set of axes we show the frequency density of the occurrences taking covariate values over 20 bins of the horizontal axis. The left set of axes shows the effect of each on the model, where the mean effect is plotted on the black line and its uncertainty is represented by the upper and lower confidence interval bounds plotted in dark grey. The figures show the fit per covariate relative to the data that correspond to specific values of the covariate.
(JPG)

S8 Fig Covariate Effect Curves for all onchocerciasis occurrences (measures of infection prevalence and disability). On the right set of axes we show the frequency density of the occurrences taking covariate values over 20 bins of the horizontal axis. The left set of axes shows the effect of each on the model, where the mean effect is plotted on the black line and its uncertainty is represented by the upper and lower confidence interval bounds plotted in dark grey.
(JPG)

S9 Fig ROC analysis for threshold. Results of the area under the receiver operating characteristic (ROC) curve analysis are presented below, with false positive rate (FPR) on the x-axis and true positive rate (TPR) on the y-axis. The red dot on the curve represents the location on the curve that corresponds to a threshold that most closely agreed with the input data. For each of the 100 BRT models, we estimated the optimal threshold that maximised agreement between occurrence inputs (considered true positives) and the mean model predictions as 0·71.
(JPG)

S1 Table Guidelines for Accurate and Transparent Health Estimates Reporting (GATHER) checklist. (DOCX)

S2 Table Total number of occurrence data classified as point and polygon inputs by diagnostic. **We present** the total number of occurrence points extracted from the input data sources by diagnostic type. ‘Other diagnostics’ include: DEC Patch test; Knott’s Method (Mazotti Test); 2 types of LAMP; blood smears; and urine tests.
(DOCX)

S3 Table Total number of occurrence data classified as point and polygon inputs by location. (DOCX)

S4 Table Covariate information. (DOCX)

S1 Text Details outlining construction of occurrence dataset. (DOCX)

S2 Text Covariate rationale. (DOCX)

S3 Text Boosted regression tree methodology additional details. (DOCX)

S1 Appendix Country-level maps and data results. Maps were produced using ArcGIS Desktop 10.6 and shapefiles to visualize administrative units are available at https://espen.afro.who.int/tools-resources/cartography-database.
(PDF)
