# Supplementary figures and images for: Predicting the environmental suitability for onchocerciasis in Africa as an aid to elimination planning
Source: PLoS Negl Trop Dis. 2021 Jul 28;15(7):e0008824. doi: 10.1371/journal.pntd.0008824 (PMC8318275; doi:10.1371/journal.pntd.0008824)

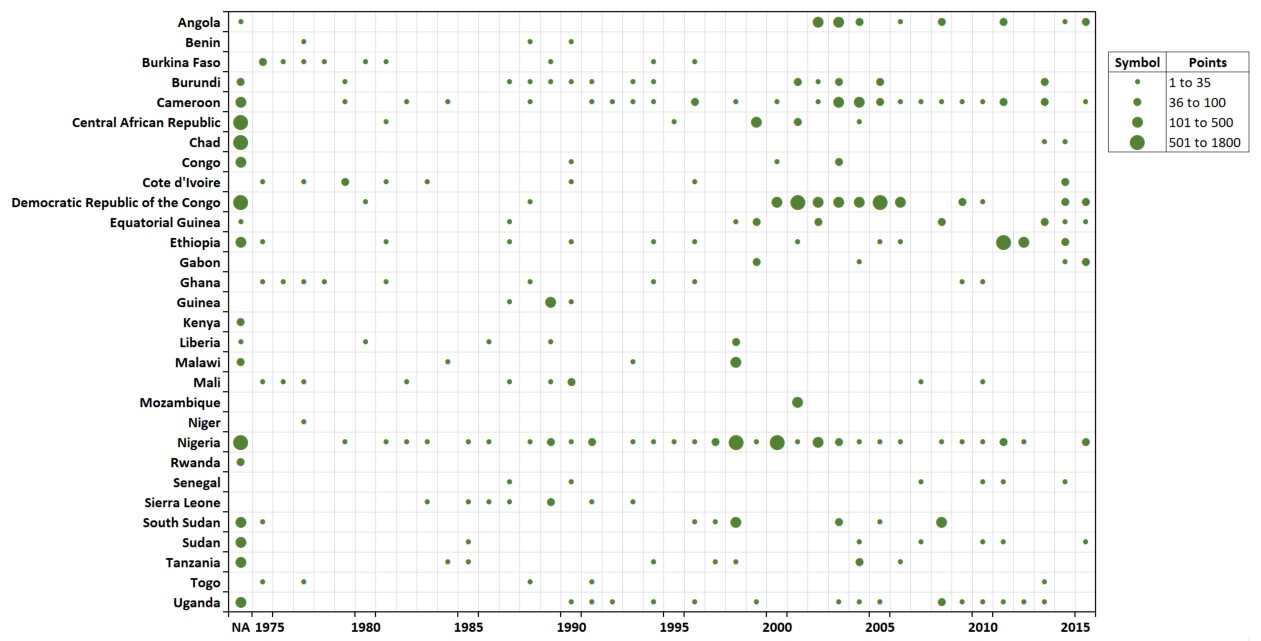

Supplement: S1 Fig — Here we visualise the volume of data used in the analysis by country and year. Larger circles indicate more data inputs. ‘NA’ indicates records for which no year was reported (eg, ‘pre-2000’). (JPG) [file pntd.0008824.s001.jpg]

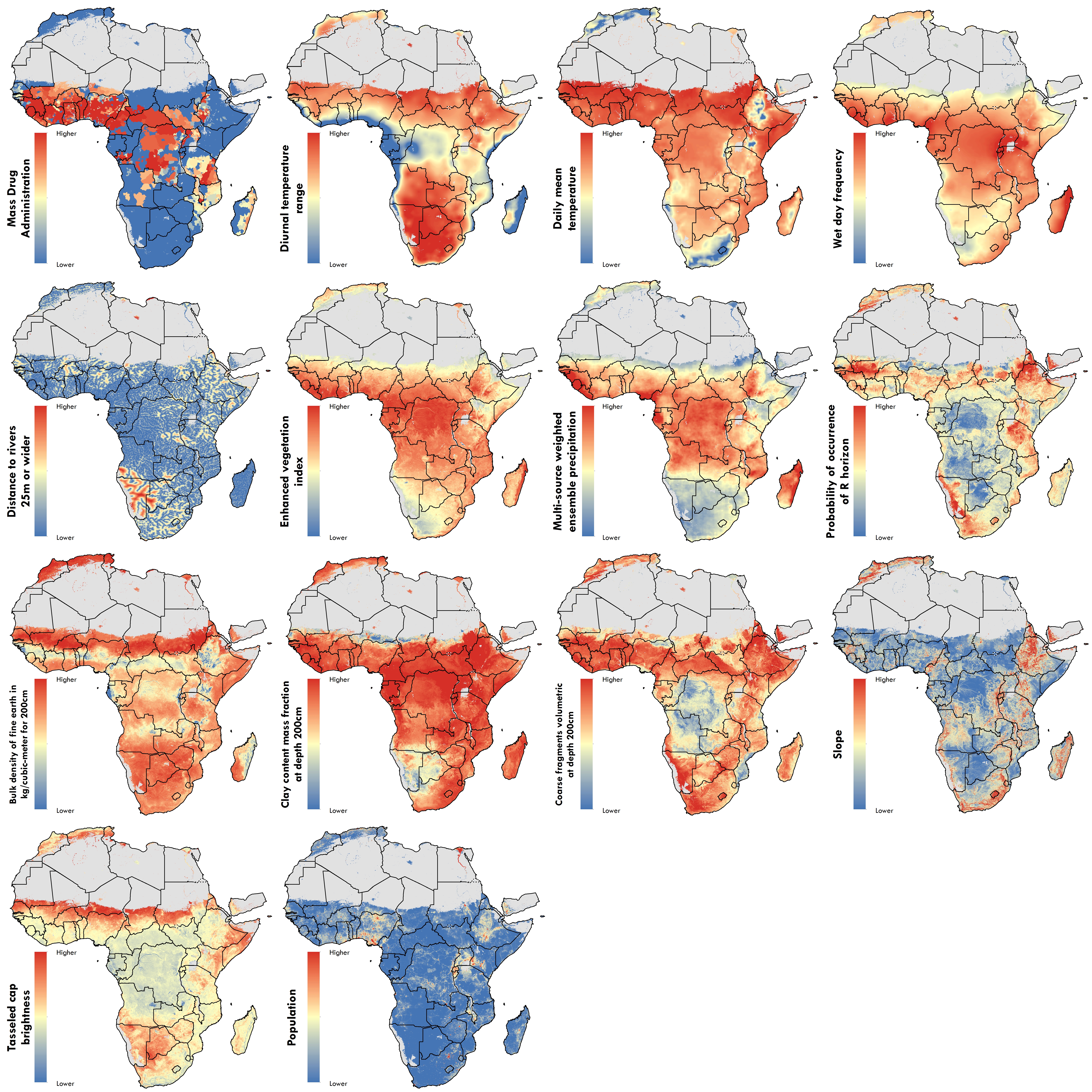

Supplement: S2 Fig — Maps were produced using ArcGIS Desktop 10.6. (JPG) [file pntd.0008824.s002.jpg]

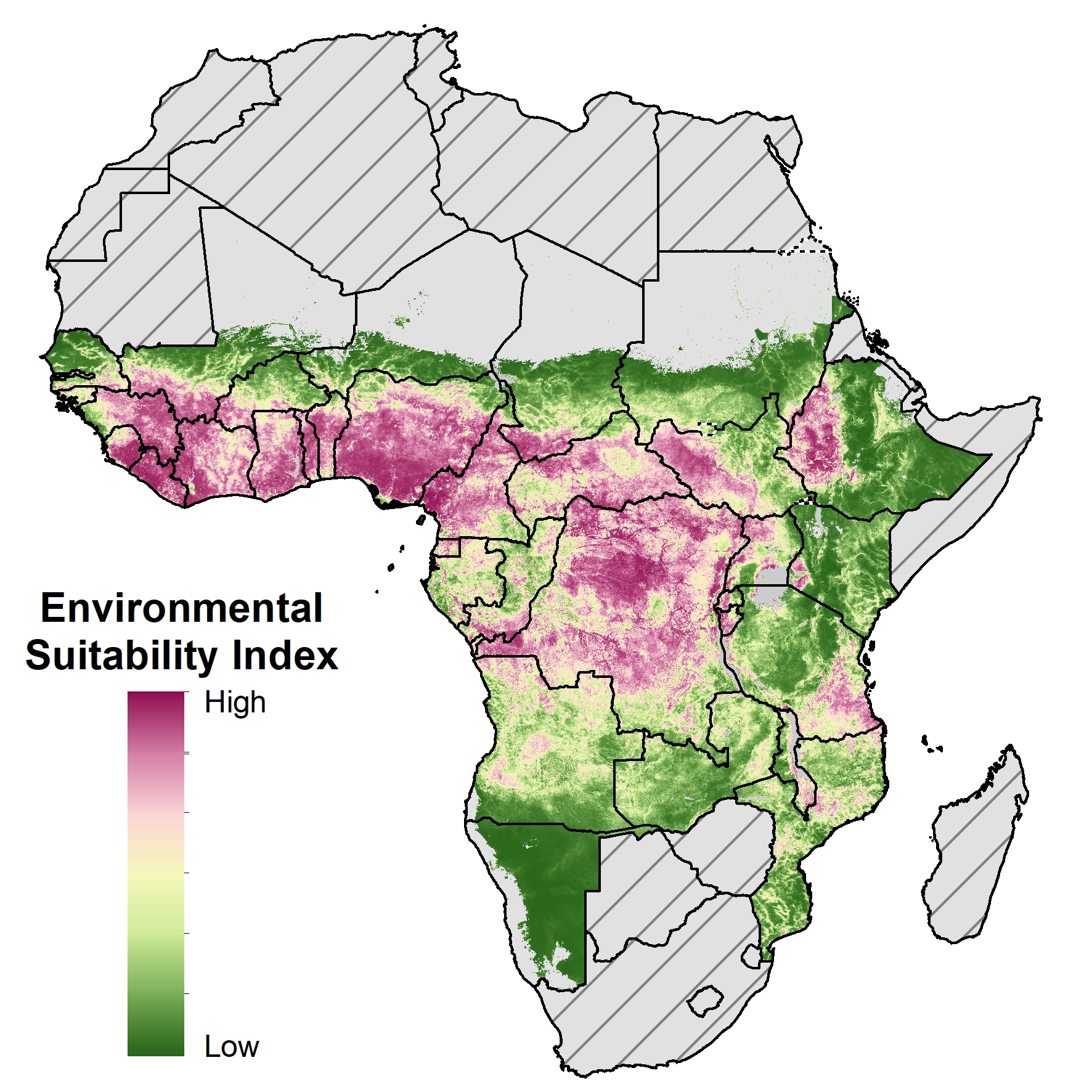

Supplement: S3 Fig — This plot shows suitability predictions from green (low = 0%) to pink (high = 100%), representing those areas where environmental conditions are most similar to prior pathogen detections. Countries in grey with hatch marks were excluded from the analysis based on a review of national endemicity status. Areas in grey only represent locations masked due to sparse population. Maps were produced using ArcGIS Desktop 10.6 and shapefiles to visualize administrative units are available at https://espen.afro.who.int/tools-resources/cartography-database. (JPG) [file pntd.0008824.s003.jpg]

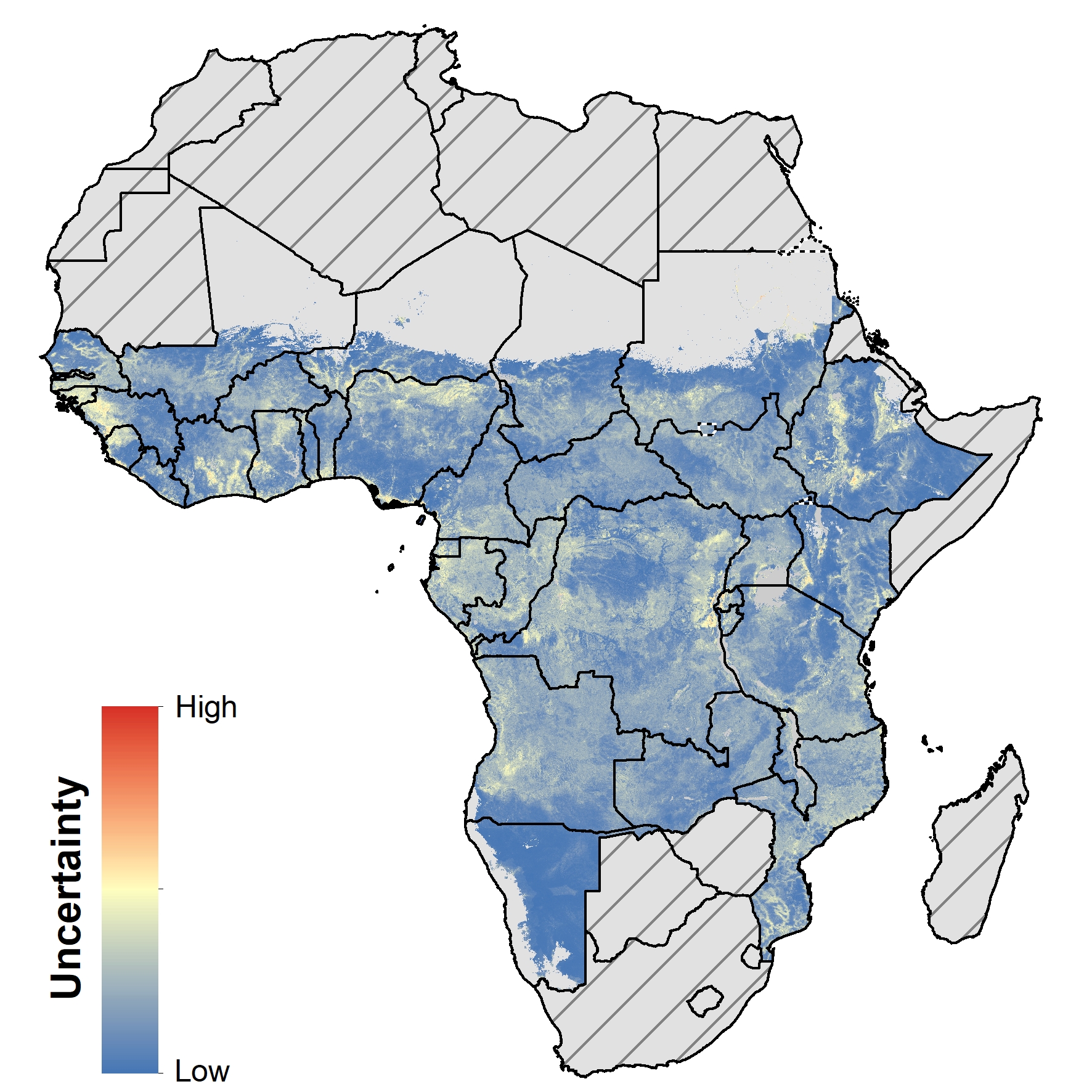

Supplement: S4 Fig — This plot shows uncertainty associated with environmental suitability predictions colored from blue to red (least to most uncertain). Countries in grey with hatch marks were excluded from the analysis based on a review of national endemicity status. Areas in grey only represent locations masked due to sparse population. Maps were produced using ArcGIS Desktop 10.6 and shapefiles to visualize administrative units are available at https://espen.afro.who.int/tools-resources/cartography-database. (JPG) [file pntd.0008824.s004.jpg]

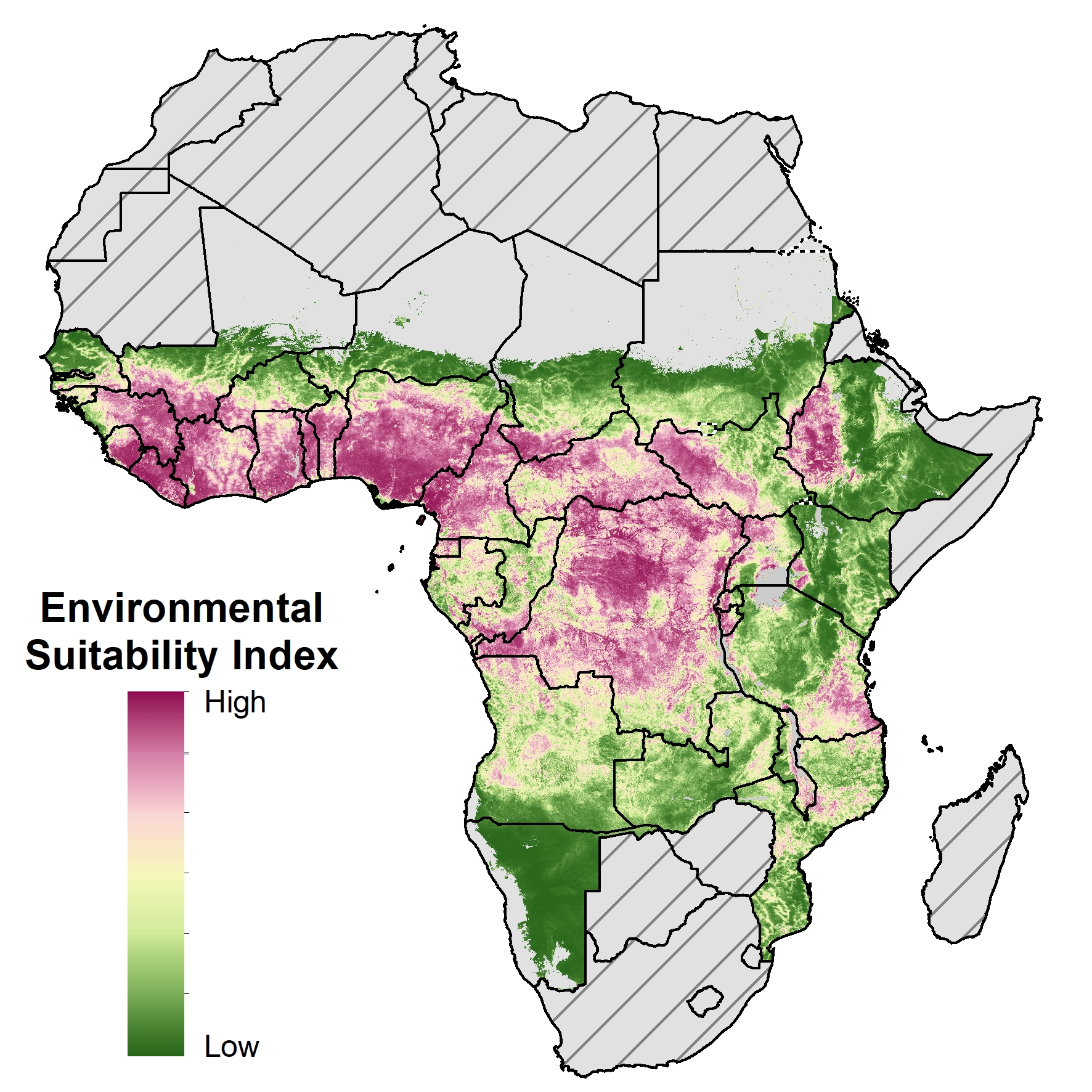

Supplement: S5 Fig — This plot shows suitability predictions from green (low = 0%) to pink (high = 100%), representing those areas where environmental conditions are most similar to prior pathogen detections. Countries in grey with hatch marks were excluded from the analysis based on a review of national endemicity status. Areas in grey only represent locations masked due to sparse population. Maps were produced using ArcGIS Desktop 10.6 and shapefiles to visualize administrative units are available at https://espen.afro.who.int/tools-resources/cartography-database. (JPG) [file pntd.0008824.s005.jpg]

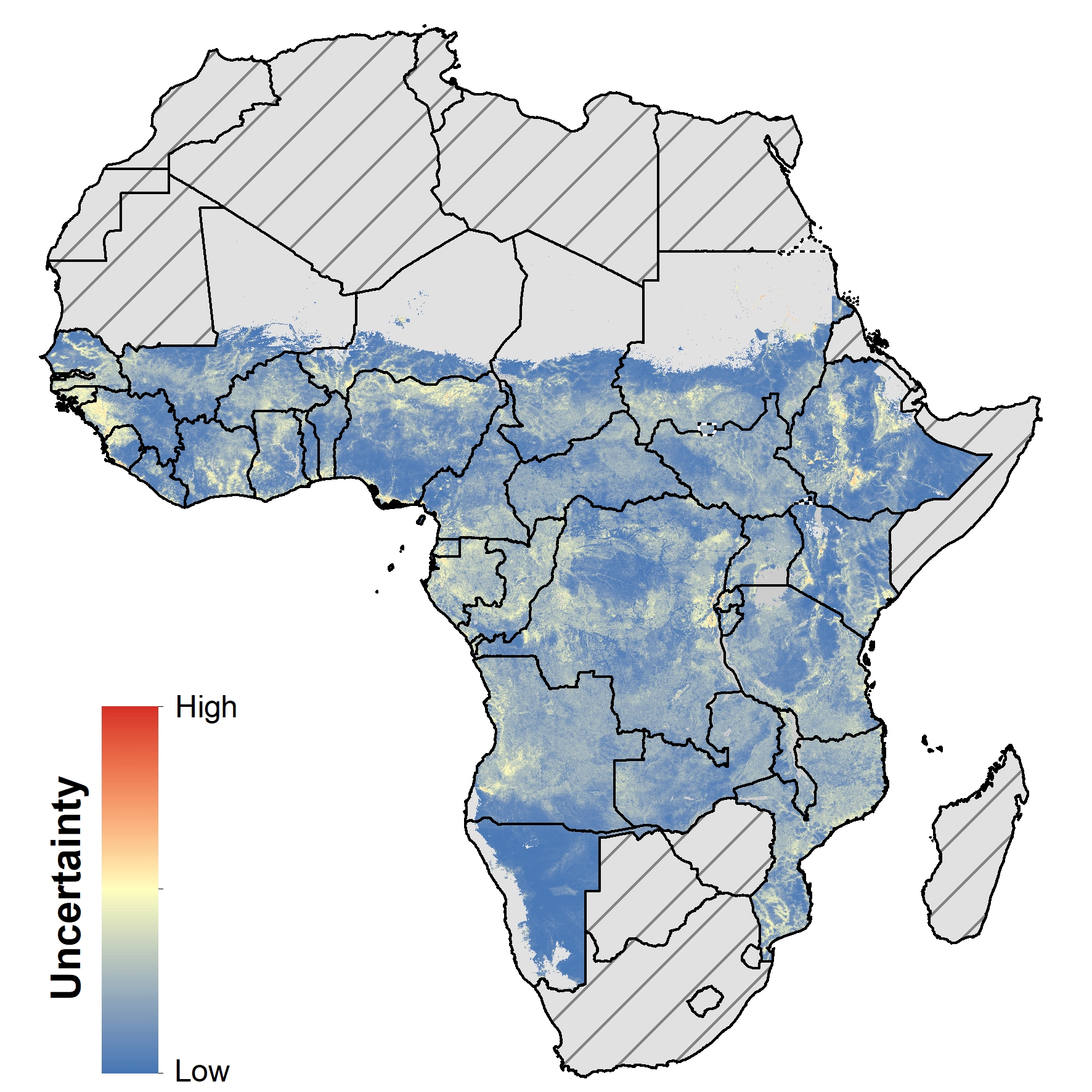

Supplement: S6 Fig — This plot shows uncertainty associated with environmental suitability predictions colored from blue to red (least to most uncertain). Countries in grey with hatch marks were excluded from the analysis based on a review of national endemicity status. Areas in grey only represent locations masked due to sparse population. (JPG) [file pntd.0008824.s006.jpg]

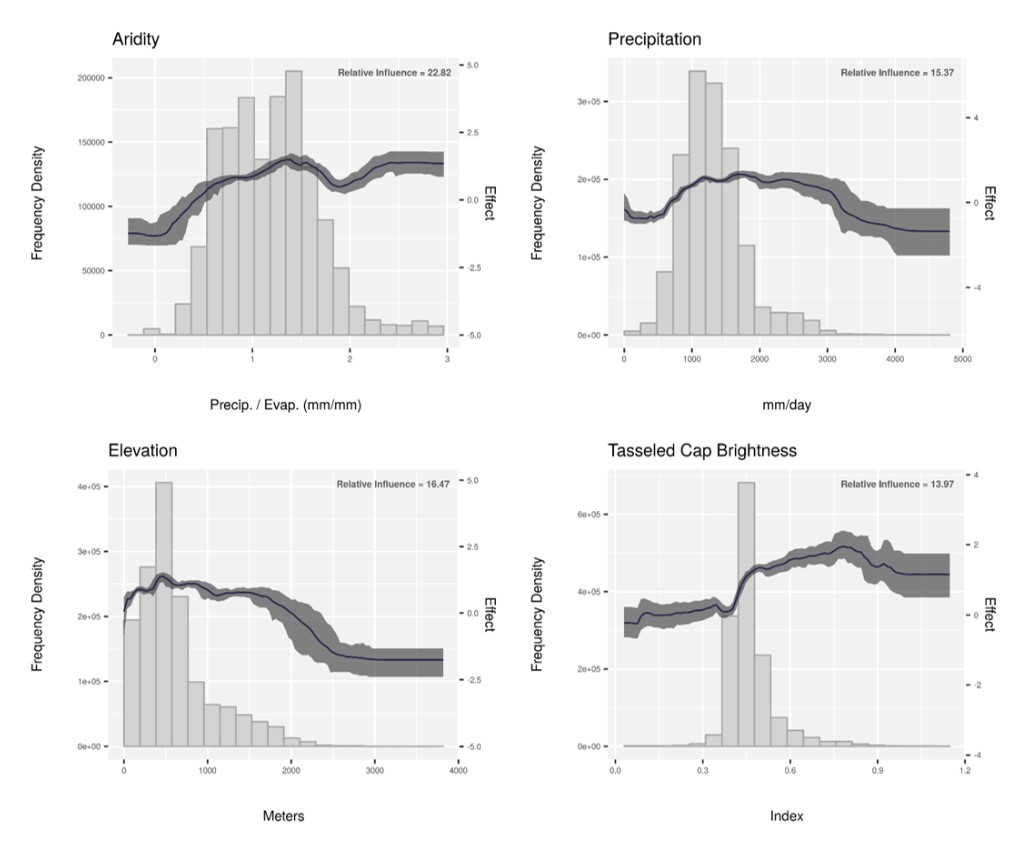

Supplement: S7 Fig — On the right set of axes we show the frequency density of the occurrences taking covariate values over 20 bins of the horizontal axis. The left set of axes shows the effect of each on the model, where the mean effect is plotted on the black line and its uncertainty is represented by the upper and lower confidence interval bounds plotted in dark grey. The figures show the fit per covariate relative to the data that correspond to specific values of the covariate. (JPG) [file pntd.0008824.s007.jpg]

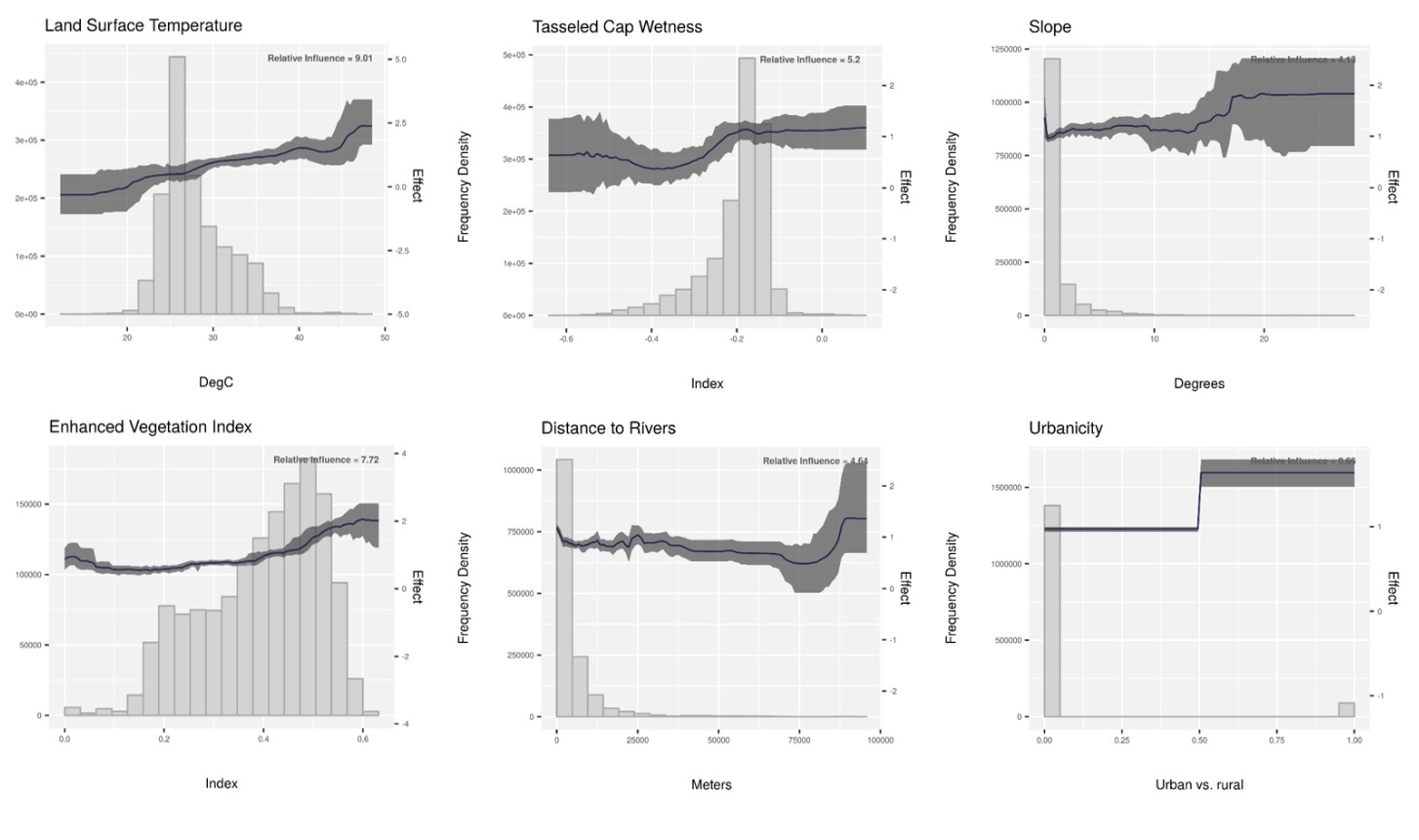

Supplement: S8 Fig — On the right set of axes we show the frequency density of the occurrences taking covariate values over 20 bins of the horizontal axis. The left set of axes shows the effect of each on the model, where the mean effect is plotted on the black line and its uncertainty is represented by the upper and lower confidence interval bounds plotted in dark grey. (JPG) [file pntd.0008824.s008.jpg]

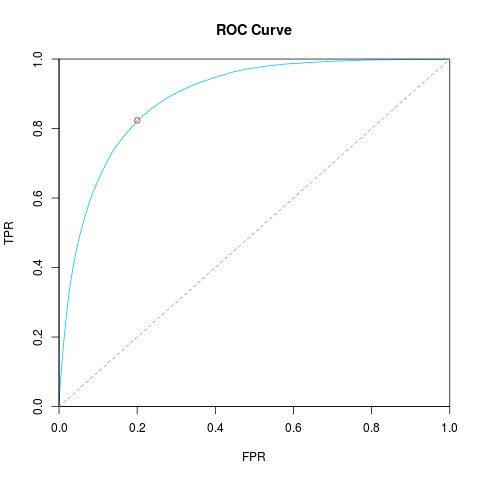

Supplement: S9 Fig — Results of the area under the receiver operating characteristic (ROC) curve analysis are presented below, with false positive rate (FPR) on the x-axis and true positive rate (TPR) on the y-axis. The red dot on the curve represents the location on the curve that corresponds to a threshold that most closely agreed with the input data. For each of the 100 BRT models, we estimated the optimal threshold that maximised agreement between occurrence inputs (considered true positives) and the mean model predictions as 0·71. (JPG) [file pntd.0008824.s009.jpg]
