# Supplementary material for: Predicting the environmental suitability for onchocerciasis in Africa as an aid to elimination planning
Source: PLoS Negl Trop Dis. 2021 Jul 28;15(7):e0008824. doi: 10.1371/journal.pntd.0008824 (PMC8318275; doi:10.1371/journal.pntd.0008824)
Supplement: S1 Table — (DOCX) [file pntd.0008824.s010.docx]

**S1 Table: Guidelines for Accurate and Transparent Health Estimates Reporting (GATHER) checklist.**

| **Item #** | **Checklist item** | **Reported in section** |
| --- | --- | --- |
| **Objectives and funding** | | |
| **1** | Define the indicator(s), populations (including age, sex, and geographic entities), and time period(s) for which estimates were made. | Main Text: Introduction, Methods (Data inputs); Methods (Covariates) |
| **2** | List the funding sources for the work. | Main Text: Acknowledgements |
| **Data inputs** | | |
| *For all data inputs from multiple sources that are synthesised as part of the study:* | | |
| **3** | Describe how the data were identified and how the data were accessed. | Main Text: Methods (Data inputs); Supplementary Information: 2.1 Protocol for literature extraction |
| **4** | Specify the inclusion and exclusion criteria. Identify all ad-hoc exclusions. | Main Text: Methods (Data inputs); Supplementary Information: 2.1 Protocol for literature extraction; 2.3 Case definition and construction of analytical dataset   \|  \| \| --- \| |
| **5** | Provide information on all included data sources and their main characteristics. For each data source used, report reference information or contact name/institution, population represented, data collection method, year(s) of data collection, sex and age range, diagnostic criteria or measurement method, and sample size, as relevant. | Main Text: Methods (Data inputs) Supplementary Information: 2.2 [Summary of input data sources](bookmark://_Toc26791605); [Supplemental Figure 1: Data sources by country and year of data collection](bookmark://_Toc26791585) |
| **6** | Identify and describe any categories of input data that have potentially important biases (eg, based on characteristics listed in item 5). | Main Text: Methods (Limitations) |
| *For data inputs that contribute to the analysis but were not synthesised as part of the study:* | | |
| **7** | Describe and give sources for any other data inputs. | N/A |
| *For all data inputs:* | | |
| **8** | Provide all data inputs in a file format from which data can be efficiently extracted (eg, a spreadsheet rather than a PDF), including all relevant meta-data listed in item 5. For any data inputs that cannot be shared because of ethical or legal reasons, such as third-party ownership, provide a contact name or the name of the institution that retains the right to the data. | GHDx link will be provided for publication |
| **9** | Provide a conceptual overview of the data analysis method. A diagram may be helpful. | Main Text: Methods; Supplementary Information 5.0 Boosted regression tree Methodology |
| **10** | Provide a detailed description of all steps of the analysis, including mathematical formulae. This description should cover, as relevant, data cleaning, data pre-processing, data adjustments and weighting of data sources, and mathematical or statistical model(s). | Main Text: Methods (Statistical Analysis); Supplementary Information 2.0 Occurrence Database |
| **11** | Describe how candidate models were evaluated and how the final model(s) were selected. | Main Text: Methods (Statistical Analysis) |
| **12** | Provide the results of an evaluation of model performance, if done, as well as the results of any relevant sensitivity analysis. | Main Text: Results; Supplemental Information: 6.0 Supplemental results |
| **13** | Describe methods for calculating uncertainty of the estimates. State which sources of uncertainty were, and were not, accounted for in the uncertainty analysis. | Main Text: Methods (Statistical analysis); Supplementary Information: [5.4 Classifying IUs based on 5 × 5-km grid-cell-level model results](bookmark://_Toc26791615) |
| **14** | State how analytic or statistical source code used to generate estimates can be accessed. | GHDx link will be provided for publication |
| **Results and discussion** | | |
| **15** | Provide published estimates in a file format from which data can be efficiently extracted. | GHDx link will be provided for publication |
| **16** | Report a quantitative measure of the uncertainty of the estimates (eg, uncertainty intervals). | Main Text: Research In Context, |
| **17** | Interpret results in light of existing evidence. If updating a previous set of estimates, describe the reasons for changes in estimates. | N/A |
| **18** | Discuss limitations of the estimates. Include a discussion of any modelling assumptions or data limitations that affect interpretation of the estimates. | Main Text: Methods (Limitations) |
