# Supplementary material for: Predicting the environmental suitability for onchocerciasis in Africa as an aid to elimination planning
Source: PLoS Negl Trop Dis. 2021 Jul 28;15(7):e0008824. doi: 10.1371/journal.pntd.0008824 (PMC8318275; doi:10.1371/journal.pntd.0008824)
Supplement: S2 Table — We present the total number of occurrence points extracted from the input data sources by diagnostic type. ‘Other diagnostics’ include: DEC Patch test; Knott’s Method (Mazotti Test); 2 types of LAMP; blood smears; and urine tests. (DOCX) [file pntd.0008824.s011.docx]

| **Diagnostic type** | **Number of occurrence points** | **Number of occurrence polygons** |
| --- | --- | --- |
| Nodule palpation | 15 455 | 11 |
| Skin snip | 2002 | 30 |
| Ov16 (ELISA or RDT) | 87 | 17 |
| Eye disease | 22 | 6 |
| Skin disease | 55 | 10 |
| Other diagnostic | 2 | 63 |
