# Supplementary material for: Predicting the environmental suitability for onchocerciasis in Africa as an aid to elimination planning
Source: PLoS Negl Trop Dis. 2021 Jul 28;15(7):e0008824. doi: 10.1371/journal.pntd.0008824 (PMC8318275; doi:10.1371/journal.pntd.0008824)
Supplement: S3 Table — (DOCX) [file pntd.0008824.s012.docx]

| **Country** | **Occurrence Points (n)** | **Occurrence Polygons (n)** |
| --- | --- | --- |
| Angola | 853 | 0 |
| Benin | 60 | 4 |
| Burkina Faso | 131 | 1 |
| Burundi | 260 | 64 |
| Cameroon | 1 089 | 11 |
| Central African Republic | 1 108 | 0 |
| Chad | 746 | 0 |
| Republic of the Congo | 413 | 2 |
| Cote d'Ivoire | 142 | 0 |
| Democratic Republic of the Congo | 4 586 | 0 |
| Equatorial Guinea | 368 | 1 |
| Ethiopia | 1 084 | 1 |
| Gabon | 155 | 0 |
| Ghana | 95 | 1 |
| Guinea | 170 | 0 |
| Kenya | 94 | 0 |
| Liberia | 113 | 1 |
| Malawi | 342 | 1 |
| Mali | 165 | 3 |
| Mozambique | 195 | 0 |
| Niger | 16 | 0 |
| Nigeria | 3 328 | 17 |
| Rwanda | 89 | 0 |
| Senegal | 45 | 3 |
| Sierra Leone | 86 | 4 |
| South Sudan | 467 | 1 |
| Sudan | 436 | 6 |
| Tanzania | 354 | 1 |
| Togo | 54 | 2 |
| Uganda | 579 | 13 |
