# Supplementary material for: Predicting the environmental suitability for onchocerciasis in Africa as an aid to elimination planning
Source: PLoS Negl Trop Dis. 2021 Jul 28;15(7):e0008824. doi: 10.1371/journal.pntd.0008824 (PMC8318275; doi:10.1371/journal.pntd.0008824)
Supplement: S4 Table — (DOCX) [file pntd.0008824.s013.docx]

**S4 Table. Covariate information.**

Presented here is a complete list of geospatial covariates with their associated temporal resolutions, sources, and references.

| **Covariate [ref]** | **Temporal resolution** | **Source** | **Original resolution** |
| --- | --- | --- | --- |
| Aridity [1,2] | Annual | Climatic Research Unit Time-Series (CRUTS) | .5 degree |
| Daytime land surface temperature [3] | Annual | MODIS | 5 km |
| Precipitation [4] | Annual | Multi-Source Weighted-Ensemble Precipitation (MSWEP) V2.1 | .1 degree |
| Distance to rivers >25m wide [5] | Static | Natural Earth Data (derived) | N/A  (vector of rivers) |
| Elevation [6] | Static | NOAA/NCEI | 1 km |
| Enhanced vegetation index (EVI) [7-10] | Annual | MODIS | 1 km |
| Urbanicity [11] | Annual | European Commission/GHS | 1 km |
| Tasseled cap brightness (TCB) [9,10,12,13] | Annual | MODIS | 1 km |
| Tasseled cap wetness (TCW) [9,10,12,13] | Annual | MODIS | 1 km |
| Slope for land surfaces | Static | NOAA/NCEI | 1 km |

References

1. Harris, I., Jones, P. d., Osborn, T. j. & Lister, D. h. Updated high-resolution grids of monthly climatic observations – the CRU TS3.10 dataset. Int. J. Climatol. 34, 623–642 (2014)
2. University of East Anglia. Climatic Research Unit TS v. 3.24 dataset. Available at: https://crudata.uea.ac.uk/cru/data/hrg/cru_ts_3.24.0 1/. (Accessed: 24th July 2017).
3. Wan, Z., Hook, S., Hulley, G. (2015). MOD11A2 MODIS/Terra Land Surface Temperature/Emissivity 8-Day L3 Global 1km SIN Grid V006 [Data set]. NASA EOSDIS Land Processes DAAC. Accessed 2019-08-06 from <https://doi.org/10.5067/MODIS/MOD11A2.006>
4. Beck, H.E., A.I.J.M. van Dijk, V. Levizzani, J. Schellekens, D.G. Miralles, B. Martens, A. de Roo: MSWEP: 3-hourly 0.25 global gridded precipitation (1979-2015) by merging gauge, satellite, and reanalysis data, Hydrology and Earth System Sciences, 21(1), 589-615, 2017. available at:

https://data.princetonclimate.com/opendap (Accessed: 21 Sept 2018)

1. Andreadis KM, Schumann GJ-P, Pavelsky T. A simple global river bankfull width and depth database. Water Resources Research. 2013;49(10):7164–8.
2. Young, A. H., K. R. Knapp, A. Inamdar, W. B. Rossow, and W. Hankins, 2017: The International Satellite Cloud Climatology Project, H-Series Climate Data Record Product, Earth System Science Data, in preparation
3. Huete, A., Justice, C. & van Leeuwen, W. MODIS vegetation index (MOD 13) algorithm theoretical basis document. (1999).
4. USGS & NASA. Vegetation indices 16-Day L3 global 500m MOD13A1 dataset. Available at: https://lpdaac.usgs.gov/dataset_discovery/modis/m odis_products_table/mod13a1. (Accessed: 25th July 2017)
5. Weiss, D. J. et al. An effective approach for gapfilling continental scale remotely sensed timeseries. Isprs J. Photogramm. Remote Sens. 98, 106–118 (2014).
6. C. Schaaf, Z. Wang. (2015). MCD43A1 MODIS/Terra+Aqua BRDF/Albedo Model Parameters Daily L3 Global - 500m V006. NASA EOSDIS Land Processes DAAC.  [http://doi.org/10.5067/MODIS/MCD43A1.006](https://mail02.ndc.nasa.gov/owa/redir.aspx?C=d8iOg4QbPKWhKvzStkXuvGFlmSiBWYwZ2UyoRRtvonwsmuff8aTTCA..&URL=http%3a%2f%2fdoi.org%2f10.5067%2fMODIS%2fMCD43A1.006)
7. Pesaresi, M. et al. Operating procedure for the production of the Global Human Settlement Layer from Landsat data of the epochs 1975, 1990, 2000, and 2014. (Publications Office of the European Union, 2016).
8. USGS & NASA. Nadir BRDF- Adjusted Reflectance Reflectance 16-Day L3 Global 1km dataset. Available at: https://lpdaac.usgs.gov/dataset_discovery/modis/m odis_products_table/mcd43b4. (Accessed: 25th July 2017)
9. Strahler, A. H. & Muller, J.-P. MODIS BRDF/Albedo product: algorithm theoretical basis document version 5.0. (1999).
10. Young, A. H., K. R. Knapp, A. Inamdar, W. B. Rossow, and W. Hankins, 2017: The International Satellite Cloud Climatology Project, H-Series Climate Data Record Product, Earth System Science Data, in preparation.
