# Supplementary material for: Predicting the environmental suitability for onchocerciasis in Africa as an aid to elimination planning
Source: PLoS Negl Trop Dis. 2021 Jul 28;15(7):e0008824. doi: 10.1371/journal.pntd.0008824 (PMC8318275; doi:10.1371/journal.pntd.0008824)
Supplement: S1 Text — (DOCX) [file pntd.0008824.s014.docx]

# **S1 Text. Occurrence database details**

## **2·1 Protocol for literature extraction**

The following methods were taken from Hill et al*.* (2019) [1] *A database of geopositioned onchocerciasis prevalence data* published in Scientific Data.

### **2·1·1 Data collection**

Published reports of onchocerciasis were identified via PubMed, Web of Science, and Scopus with the following search terms: “Oncho”, “river blindness”, “*O. Volvulus*”, “robles disease”, “blinding filariasis”, “coast erysipelas”, and “sowda”. The search was for all articles published about onchocerciasis prior to July 7, 2017. We identified 2 502 unique records. We then collated these results and conducted title-abstract screenings manually to remove any publications that did not report onchocerciasis prevalence among humans, case study articles, or publications solely reporting diagnostic test research and development. A total of 579 articles underwent full text review provided they included onchocerciasis prevalence in human subjects reported from the year 1975 or later, were original sources, and did not include case-control studies; we excluded a further 320 sources that did not meet these inclusion criteria. Citations were reviewed to ensure relevant articles were retroactively added to the database if not already included; this identified 18 additional articles. Geographical data, as well as relevant epidemiological data, were extracted from 259 peer-reviewed sources.

### **2·1·2 Geo-positioning of data**

Geographical data were extracted manually at the most detailed location possible using either Google Maps or ArcGIS (https://www.esri.com/en-us/home). If prevalence data were reported from a location within a 5 × 5-km area, the geography was defined as a point, and represented by a specific latitude and longitude. This definition of a ‘point’ referencing an area smaller than 5 × 5-km was chosen for compatibility with satellite imagery, typically resolved at the 5 × 5-km spatial level for large-scale analyses.

If the location of prevalence data represented an area exceeding 5 × 5-km (eg, a group of villages), or if the location represented a general area (eg, a health district), a polygon was assigned to cover the region of the reported occurrence, as described in the original article. Full details of this process are reported within Hill et al. (2019) [1].

## **2·3 Case definition and construction of analytical dataset**

We constructed analytical datasets using three different case definitions for an ‘occurrence’:

(1) Any occurrence reported using prevalence of onchocerciasis measured by nodule palpation, serology (Ov16), skin snip positivity (mf) or other diagnostics, as well as presence of onchocerciasis-related disability (eye or skin disease). The results of analysis using this case definition are presented in the main text of the paper.

(2) Any occurrence reported using prevalence of onchocerciasis measured by nodule palpation, serology (Ov16), skin snip positivity (mf) or other diagnostics, including locations for which onchocerciasis prevalence was zero in surveys conducted after MDA implementation and no pre-MDA data were available at that location. The results of this analysis are presented in Supplemental Appendix, Section 6.

(3) Any occurrence reported using prevalence of onchocerciasis measured by nodule palpation, serology (Ov16), skin snip positivity (mf) or other diagnostics, excluding data on onchocerciasis-related morbidity. The results of this analysis are presented in Supplemental Appendix, Section 6.

Classification of implementation units (IUs) endemicity was obtained from the shapefile maintained by ESPEN and available for download at: <https://espen.afro.who.int/>. Accession date: June 19, 2018.

### **2·3·1 De-duplication of input data**

Data sources were compared to remove duplicate information. For some locations, multiple prevalence estimates were reported using different diagnostic tests. If two or more diagnostic tests reported conflicting results (eg, one test reported zero prevalence and a second test reported non-zero prevalence) for the same location, those locations were classified as occurrences.

The study dataset is currently hosted at: http://ghdx.healthdata.org/record/ihme-data/africa-onchocerciasis-environmental-suitability-geospatial-estimates

R code to create analytical datasest:

source('FILEPATH/setup.R')

package_list <- c('seegSDM', 'dismo', 'maptools', 'rgdal', 'rgeos')

load_R_packages(package_list)

setwd('FILEPATH/oncho')

covs <- brick('FILEPATH/covs.grd')

bias_grid_final <- raster('FILEPATH/bias_grid_final_no_espen.tif')

pts_buffer <- shapefile('FILEPATH/pts_buffer.shp')

dat.orig <- read.csv('ExclusionMapAllData.csv')

dat.pts <- dat.orig[dat.orig$presence == 1,]

dat.pts <- dat.pts[,c("long", "lat")]

dat.pts <- na.omit(dat.pts)

names(dat.pts) <- c("longitude", "latitude")

dat.pts <- cbind(PA = rep(1, nrow(dat.pts)), dat.pts)

bg <- data.frame(bgSample(n = nrow(dat.pts),

raster = mask(bias_grid_final, pts_buffer),

prob=T))

names(bg) <- c('longitude', 'latitude')

dat <- rbind(dat.pts,

cbind(PA = rep(0, nrow(bg)), bg))

dat_covs <- extract(covs, dat[,2:3])

dat_all <- cbind(dat, dat_covs)

dat_all <- na.omit(dat_all)

write.csv(dat_all, file = "FILEPATH/dat_all_pts.csv", row.names=F)

**References:**

1. Hill E, Hall J, Letourneau ID, et al. A database of geopositioned onchocerciasis prevalence data. Sci Data 2019; 6: 1–6.
