# Supplementary material for: Predicting the environmental suitability for onchocerciasis in Africa as an aid to elimination planning
Source: PLoS Negl Trop Dis. 2021 Jul 28;15(7):e0008824. doi: 10.1371/journal.pntd.0008824 (PMC8318275; doi:10.1371/journal.pntd.0008824)
Supplement: S2 Text — (DOCX) [file pntd.0008824.s015.docx]

## **S2 Text. Covariate rationale**

The covariates used in the analysis were chosen to account for environmental factors known to influence transmission of onchocerciasis. All covariate datasets were resampled to the 5x5km resolution prior to analysis.

- Aridity, temperature, and precipitation were chosen to account for climatic factors that would be associated with vector presence [1,2].
- We included elevation, slope (eg, the grade of elevation across the 5 × 5-km), and distance to rivers (>25m wide) to account for the fact that *Similium* species breed near fast-flowing rivers. Data are available to generate a covariate on rivers as small as 10m in width; we chose to use a larger threshold of 25m to (1) exclude the possibility of water sources that would likely not be representative of breeding sites and (2) avoid saturating the covariate when the raw river data was aggregated to the 5 × 5-km resolution [3-5].
- Urbanicity was included to account for the reduced vectorial capacity among urban areas.
- Enhanced vegetation index (EVI), tasseled cap brightness (TCB), and tasseled cap wetness (TCW) together are the axes of a coordinate system for land surface analysis [7,8]. As such, they allow the model to account for geographical variation due to differences in relevant factors of fly-human interaction such as vegetation, soil, and moisture. These covariates also serve as proxies for density of vegetation, which serves as a proxy for forest density [7,8].

Covariate values are visualised in S3 Fig.

We extracted covariate data at each occurrence and background location in the dataset. For point-level data, covariate values were extracted at the coordinates; for polygonal data, covariates were assigned values by making n-many random draws within the polygonal region for each bootstrap, where n is the number of sampling sites (if reported). Where the number of sampling sites was not available, we imputed the value of n for that polygon by estimating the frequency density of n across all polygons and drawing from this distribution in each bootstrap to arrive at the number of random draws made within said polygon to represent instances of uncertainty in the environmental factors associated with transmission.

**References:**

1 Cheke RA. The thermal constant of the onchocerciasis vector *Simulium damnosum* s.l. in West Africa. *Med Vet Entomol* 2012; **26**: 236–8.

2 Nwoke B, Onwuliri C, Ufomadu G. Onchocerciasis in Plateau State; Nigeria: ecological background, local disease perception & treatment; and vector/parasite dynamics. *J Hyg Epidemiol Microbiol Immunol* 1992; **36**: 153–60.

3 Winthrop KL, Furtado JM, Silva JC, Resnikoff S, Lansingh VC. River Blindness: An Old Disease on the Brink of Elimination and Control. *J Glob Infect Dis* 2011; **3**: 151–5.

4 Duke BO. The differential dispersal of nulliparous and parous *Simulium damnosum*. *Tropenmed Parasitol* 1975; **26**: 88–97.

5 Crosskey RW. The Natural History of Blackflies. Chichester: John Wiley and Sons, 1990.

6 Thompson BH. Studies on the flight range and dispersal of *Simulium damnosum* (Diptera: Simuliidae) in the rain-forest of Cameroon. *Ann Trop Med Parasitol* 1976; **70**: 343–54.

7 Xiaoyang Zhang, Schaaf C, Friedl M, Strahler A, Feng Gao, Hodges. MODIS tasseled cap transformation and its utility. 2002: 1063–5.

8 Crist EP, Cicone RC. A Physically-Based Transformation of Thematic Mapper Data-The TM Tasseled Cap. *IEEE Trans Geosci Remote Sens* 1984; **GE-22**: 256–63.
