# Supplementary material for: Predicting the environmental suitability for onchocerciasis in Africa as an aid to elimination planning
Source: PLoS Negl Trop Dis. 2021 Jul 28;15(7):e0008824. doi: 10.1371/journal.pntd.0008824 (PMC8318275; doi:10.1371/journal.pntd.0008824)
Supplement: S3 Text — (DOCX) [file pntd.0008824.s016.docx]

# **S3 Text. Boosted regression tree methodology additional details**

Boosted regression trees (BRTs) have been shown to have superior performance [1] relative to other species distribution models. The target of inference for this approach is the presence of a given species as a function of chosen covariates. The approach is spatially explicit; covariate values for each occurrence input are matched based on geographical location. In brief, boosted regression trees contrast reports of the pathogen of interest with a set of either location for which absences are known. Alternatively, in the case of pathogens for which definitive absences are unavailable, a set of background data is generated to represent areas of potential absence, similar to the purpose of controls in a case-control study design, whereby the controls represent the source population that gave rise to the cases.

The BRT methodology combines classification trees with boosting algorithms, where previous trees inform subsequent models to iteratively improve model fit. Using a train-test split in which 20% of the data is held out in each iteration, the trees that best categorise the known occurrences and defined background data are then selected [2]. Performing this categorisation allows the model to account for the interactions between environmental variables [3].

After the model is fit, predictions can be generated onto covariate values across a defined extent. The prediction is on a 0 to 1 scale, where 1 indicates that the covariate values in the location of interest exactly match the modelled environment profile. As a consequence, we can evaluate the environmental similarity of any location, regardless of disease reporting status, and by assuming that environmental signals are indicative of pathogen occurrence.

## **5·1 Areal Polygon Data Triage**

Polygon data presents a unique challenge for these kinds of models since they do not align with the covariate values at a single location. As such, it is not possible to extract a particular set of covariate values as performed with point data (geo-referenced within the 5 × 5-km grid cell). In order to accommodate these data sources in the analysis, we draw a random, uniform sample of longitude-latitude pairs within the polygon’s boundary. We then average the covariate values across these samples to account for the spatial variability in covariate values.

## **5·2 Bayesian hyperparameter optimisation**

Boosted regression trees require three hyperparameter values to be identified: the number of leaves of each learned tree (tree complexity); the weighting assigned to previously learned models (learning rate); and the number of trees [2,3]. Since it is not possible to set *a priori* the value of these hyperparameters to ensure optimum model fit, we test the regression’s fit for a given set of hyperparameters on a chosen objective function. Bayesian optimisation allows us to select suitable values for each hyperparameter from an arbitrary, finite hyperparameter space. We may choose an optimal set of these hyperparameters by sequentially updating the posterior distribution over the hyperparameter space via an acquisition function. We chose this optimisation method to balance the computational efficacy of a random search over the hyperparameter space and the reliability of the deterministic grid search. We use the Scikit-Optimize Python package in order to perform the optimization [4]. The Bayesian hyperparameter optimisation is repeated 150 times over a random training subset of 80% of the entire study dataset, and selects a set of hyperparametersas a result of a 10-fold cross-validation procedure of the resulting 150 choices. Choosing 150 iterations provides us with enough data to perform 10-fold cross-validation. Then, the selected hyperparameters are validated by comparing the model fit on the training set to the remaining 20% of the data.

## **5·3 Defining domain for background data**

We chose to exclude reported absences of onchocerciasis infection as methods to diagnose the prevalence of onchocerciasis do not have high sensitivity, particularly in low-prevalence settings. This is primarily a concern due to the volume of input data for which onchocerciasis prevalence was measured according to presence of palpable nodules. To create a background sample, we first restricted the geographical extent to only include locations within known endemic countries. We then further restricted background sampling from locations within implementation units considered for onchocerciasis elimination mapping (as reported by ESPEN). Using a domain for background sampling outside of the countries for which onchocerciasis is known or considered endemic could lead to bias and regression trees that prioritise the extreme range of covariate values, such as extremes in temperatures or elevation, leading to homogenous model outputs for areas in Africa (the geographical extent of inference) [5].

Background points were sampled independently across 100 bootstraps and uniformly from within 100 km of the polygon boundaries and point locations such that the number of samples from each region matched the number of occurrence records associated with it. Choosing to sample from within 100 km of occurrence data locations allows us to only consider environmental conditions roughly similar to those where transmission is known to take place; resulting in a good set of controls to our case data. We sampled once for regions associated with point data, but n times for polygons, where n equals the number of communities sampled within the polygon (if reported). Since the 100-km regions would overlap with the IUs for which endemicity status was unknown (and ultimately the target of inference), we restricted the sampling so that no locations were selected from IUs known as endemic, and avoided sampling within polygonal locations in the presence dataset. We used a shapefile provided by ESPEN to identify IUs (Supplemental Appendix Figure 1) for inclusion in the background sample.

## **5·4 Classifying IUs based on 5 × 5-km grid-cell-level model results**

A more easily interpretable metric for environmental suitability indices is a binary classification of regions of ‘risk’. Since the model predicts environmental suitability as a value between 0 and 1, we tested thresholds in 0·01 increments (from 0 to 1) to determine a prediction threshold that would minimise the classification error associated with the model (ie, a value that maximises the ratio of occurrence classified as ‘environmentally suitable’ and background records appropriately classified as not). We identify this threshold as the value corresponding with the point on the receiver operating characteristic (ROC) curve that minimised the distance between the true positive rate (sensitivity) and the false positive rate (1–specificity). As the objective of the analysis was to identify environmentally suitable IUs, we compared the values of the largest mean prediction within each IU against the threshold identified by the ROC analysis.

## **5·5 Model fit statistics**

The two fit statistics used to evaluate model fit are RMSE and AUC. The BRT model computes relationships between the input data (occurrences and background sample) and environmental covariates, which are then used to generate predictions. We calculate fit statistics using the individual bootstraps’ associated covariate values (RMSE_boot_ and AUC_boot_). We evaluate the success of the regression in accurately representing known occurrences as highly suitable, which corresponds with a low value of RMSE_boot_ and high value of AUC_boot._

**References**

1 Elith* J, Graham* CH, Anderson RP, *et al.* Novel methods improve prediction of species’ distributions from occurrence data. *Ecography* 2006; **29**: 129–51.

2 Friedman JH. Stochastic gradient boosting. *Comput Stat Data Anal* 2002; **38**: 367–78.

3 Elith J, Leathwick JR, Hastie T. A working guide to boosted regression trees. *J Anim Ecol* 2008; **77**: 802–13.

4 Head T, MechCoder, Louppe G, *et al.* scikit-optimize/scikit-optimize: v0.5.2. Zenodo, 2018 DOI:10.5281/zenodo.1207017.

5 Barbet‐Massin M, Jiguet F, Albert CH, Thuiller W. Selecting pseudo-absences for species distribution models: how, where and how many? *Methods Ecol Evol* 2012; **3**: 327–38.
