# Supplementary material for: Predicting the environmental suitability for onchocerciasis in Africa as an aid to elimination planning
Source: PLoS Negl Trop Dis. 2021 Jul 28;15(7):e0008824. doi: 10.1371/journal.pntd.0008824 (PMC8318275; doi:10.1371/journal.pntd.0008824)

Angola

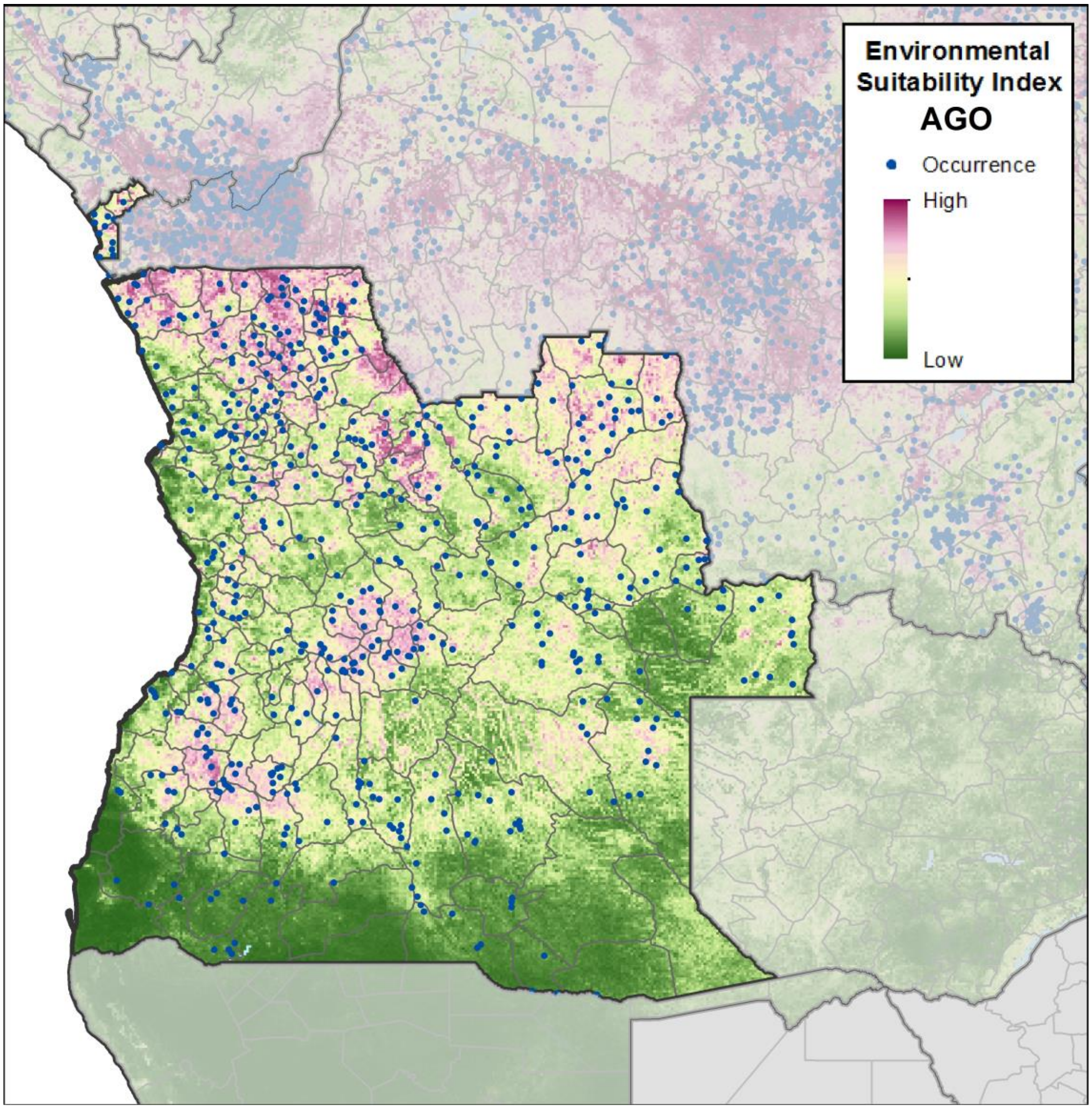

Benin

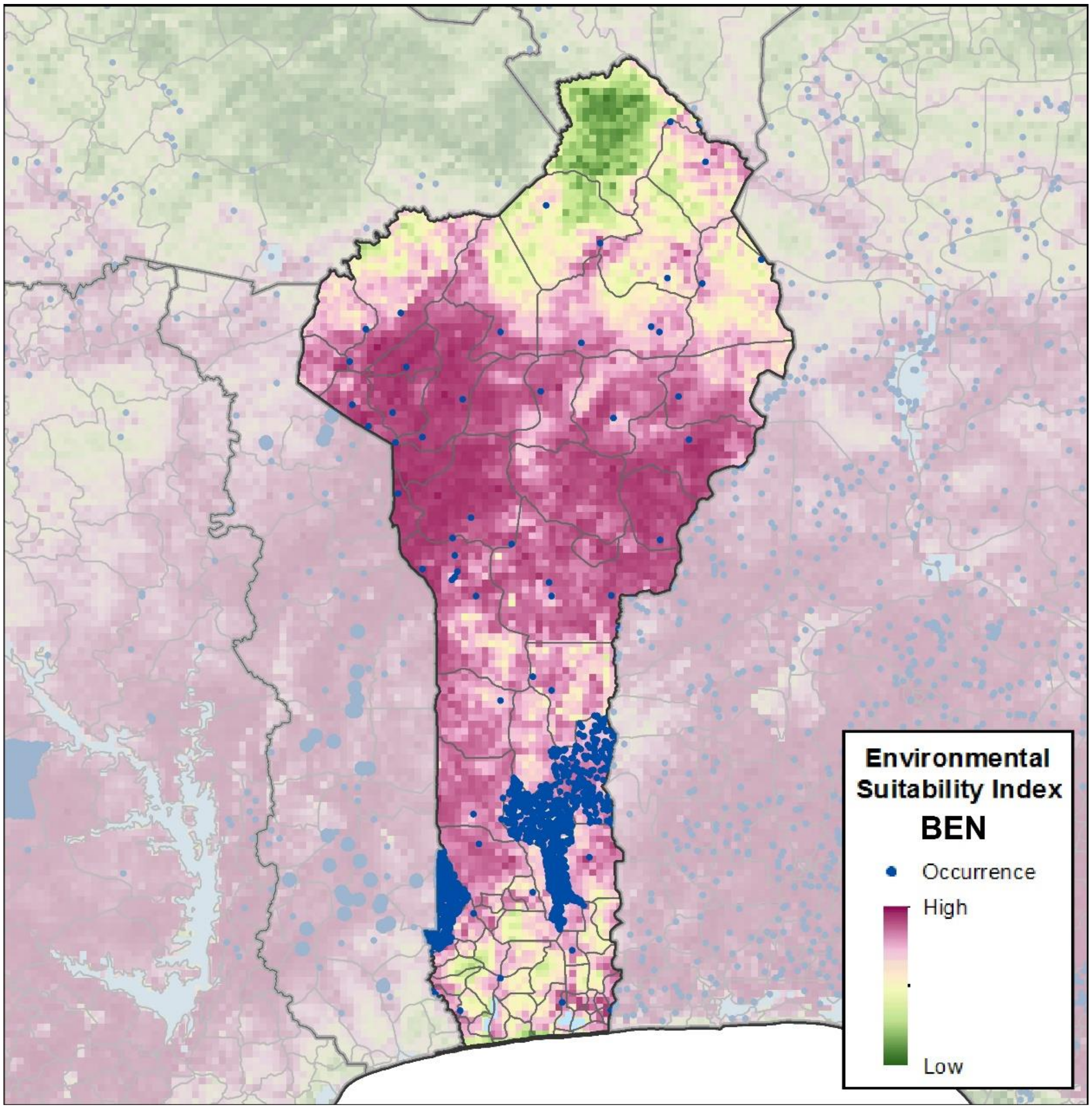

Burkina Faso

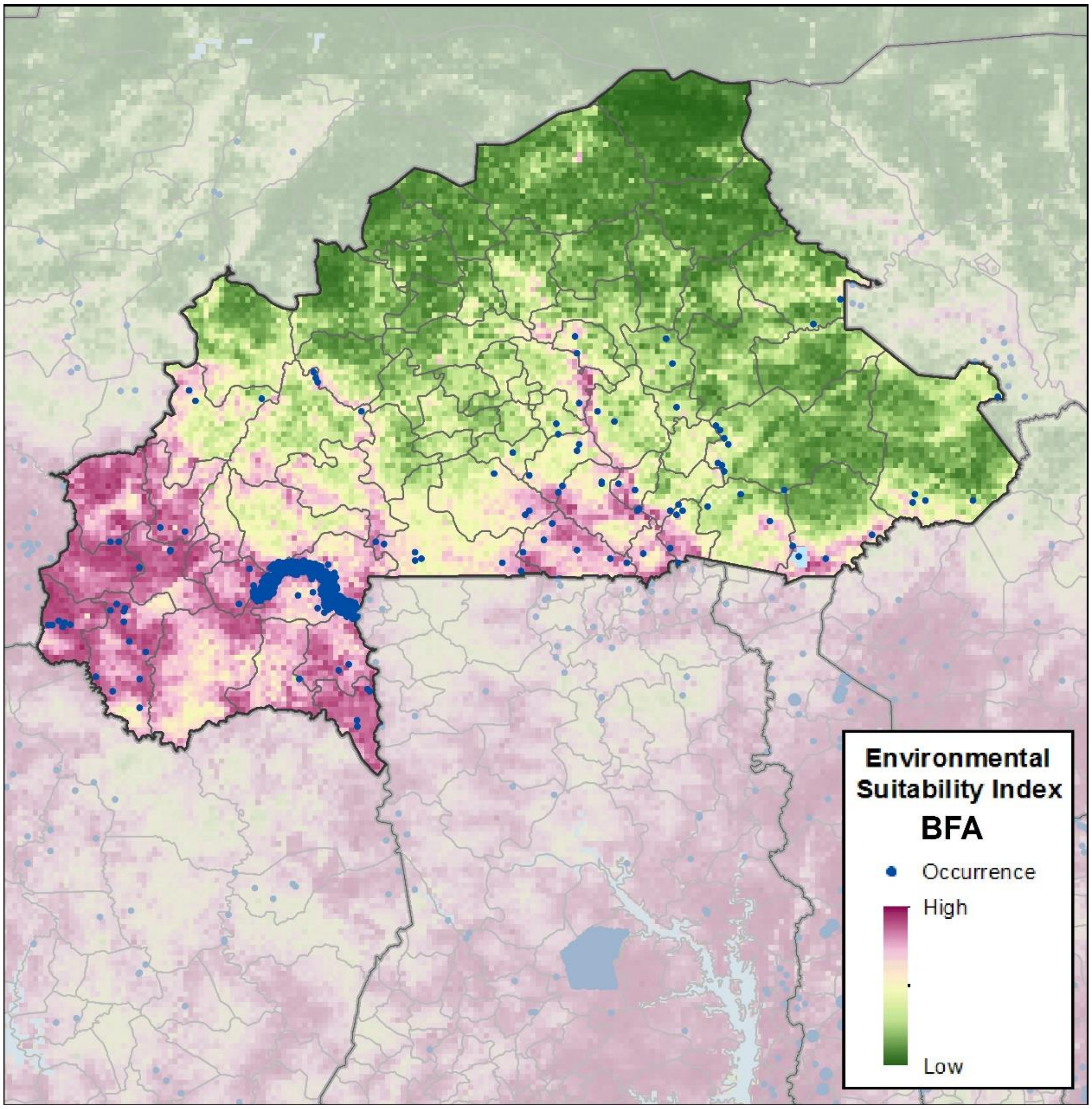

Burundi

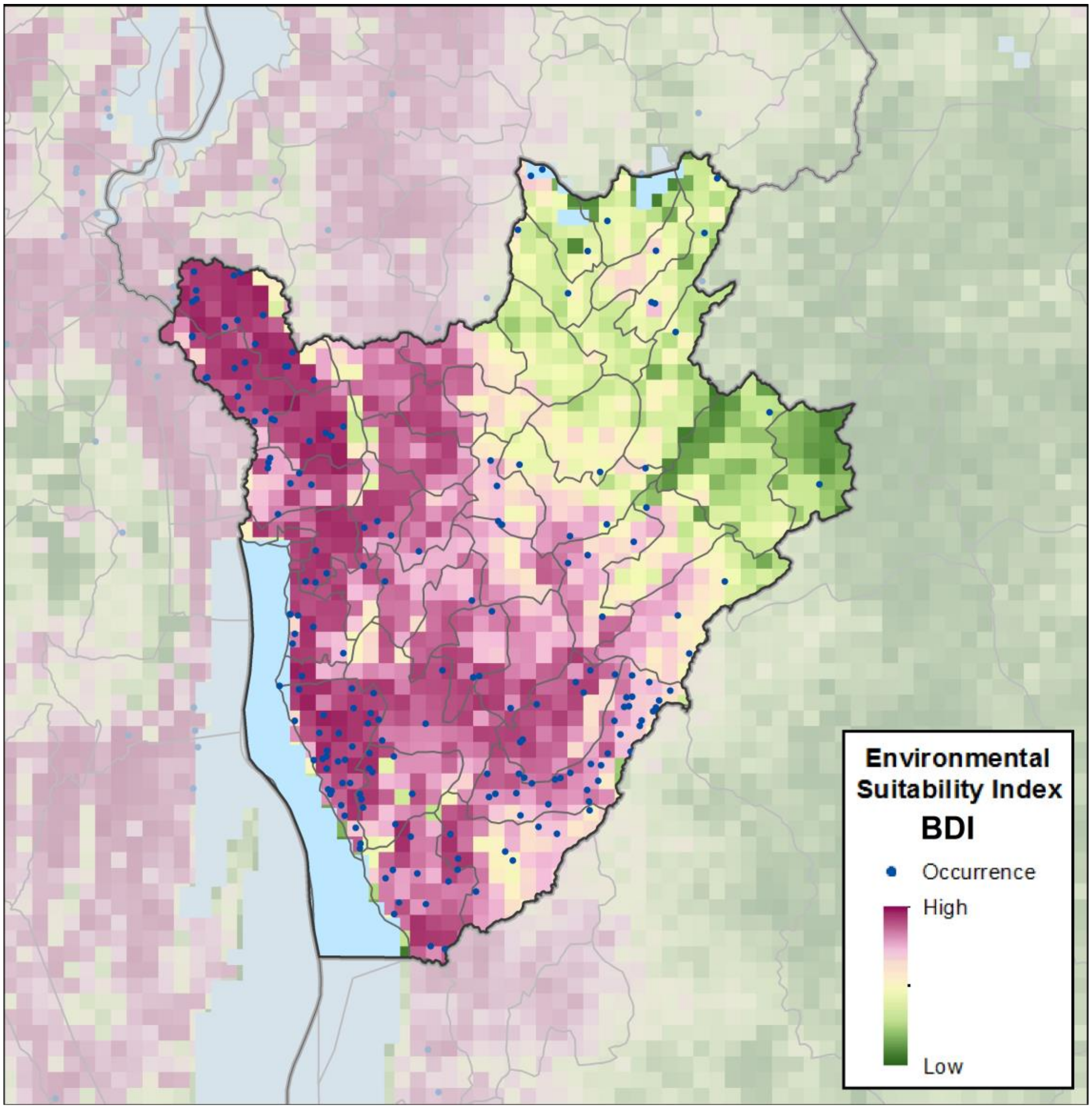

Cameroon

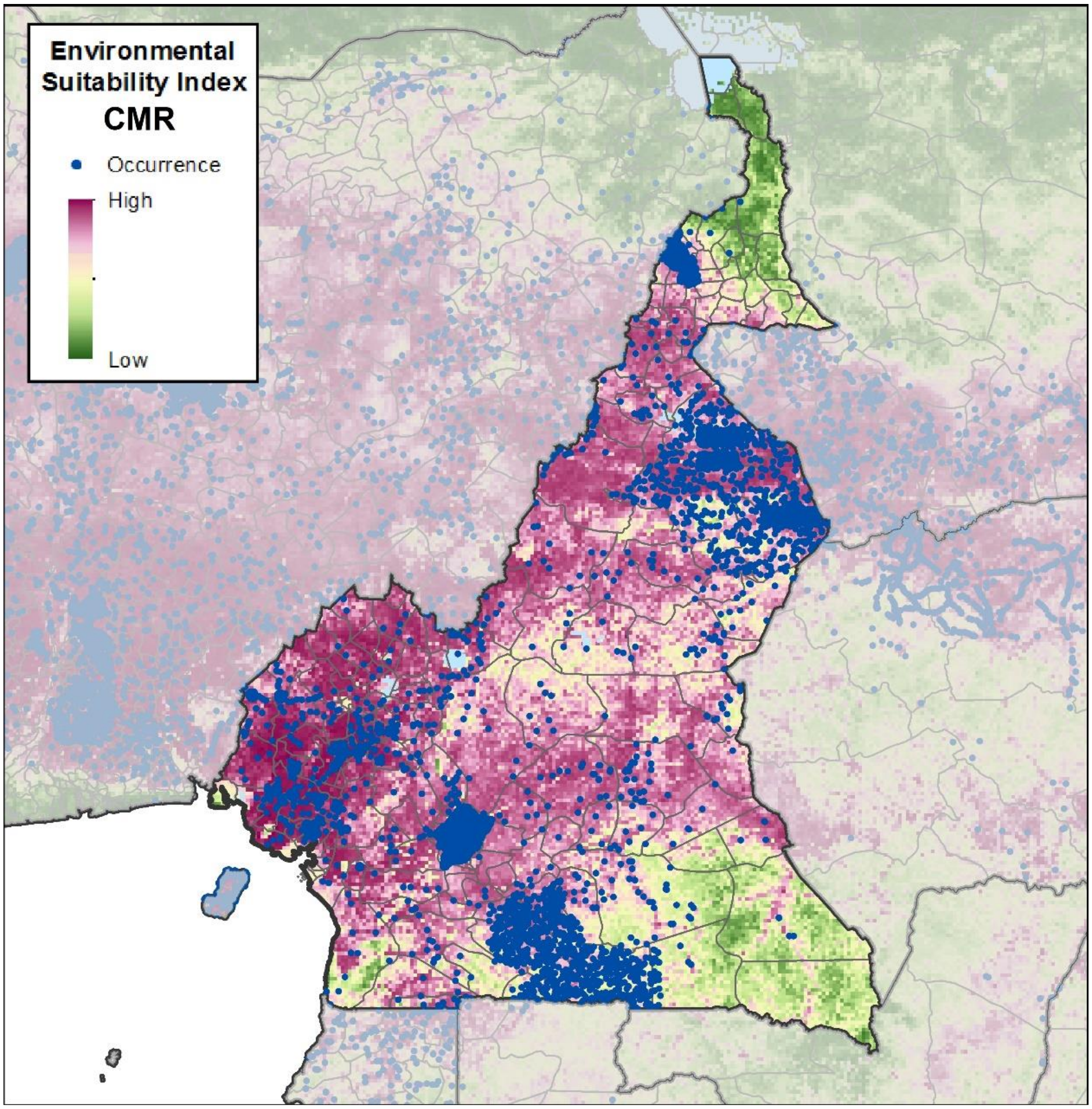

Central African Republic

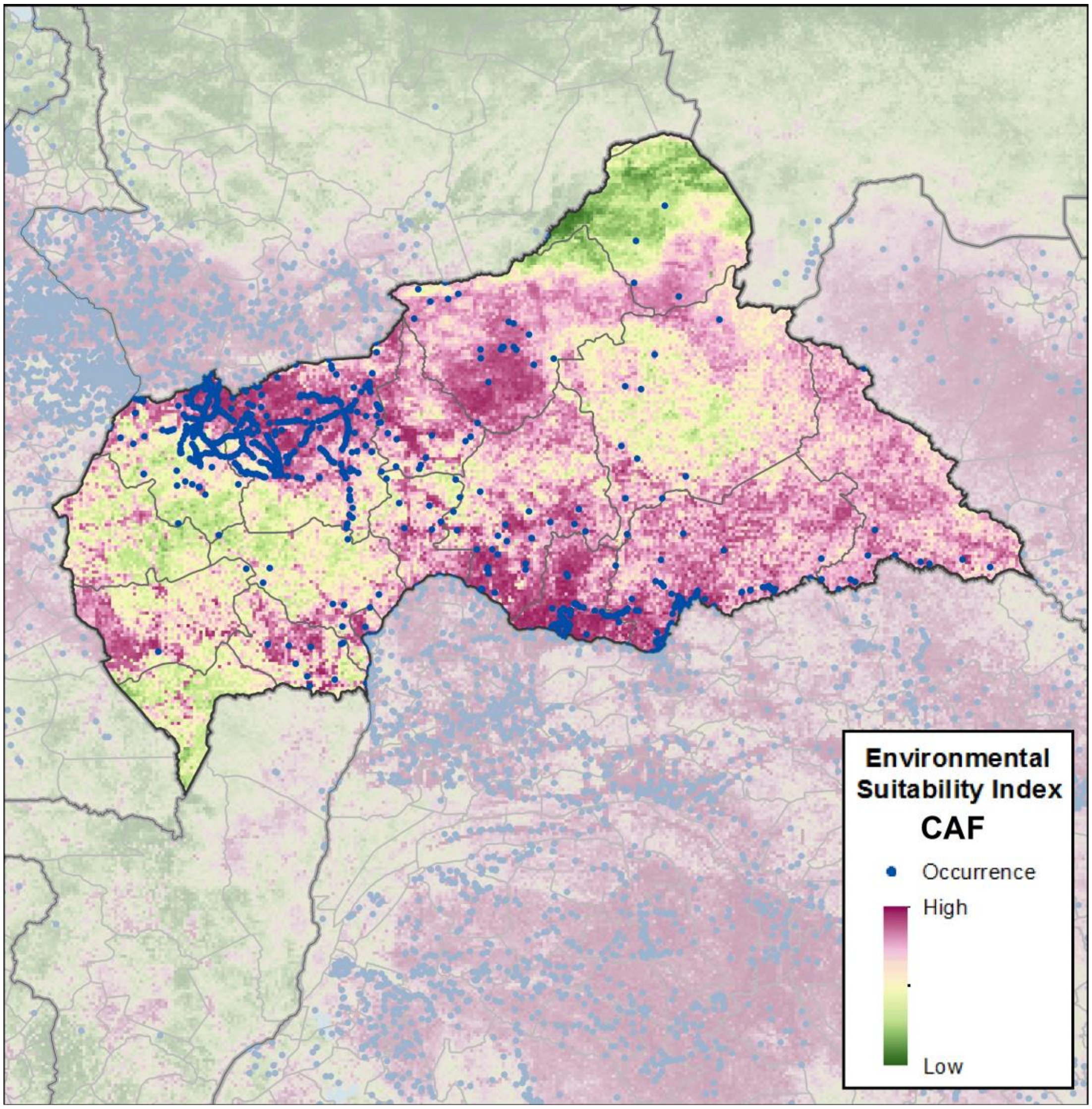

Chad

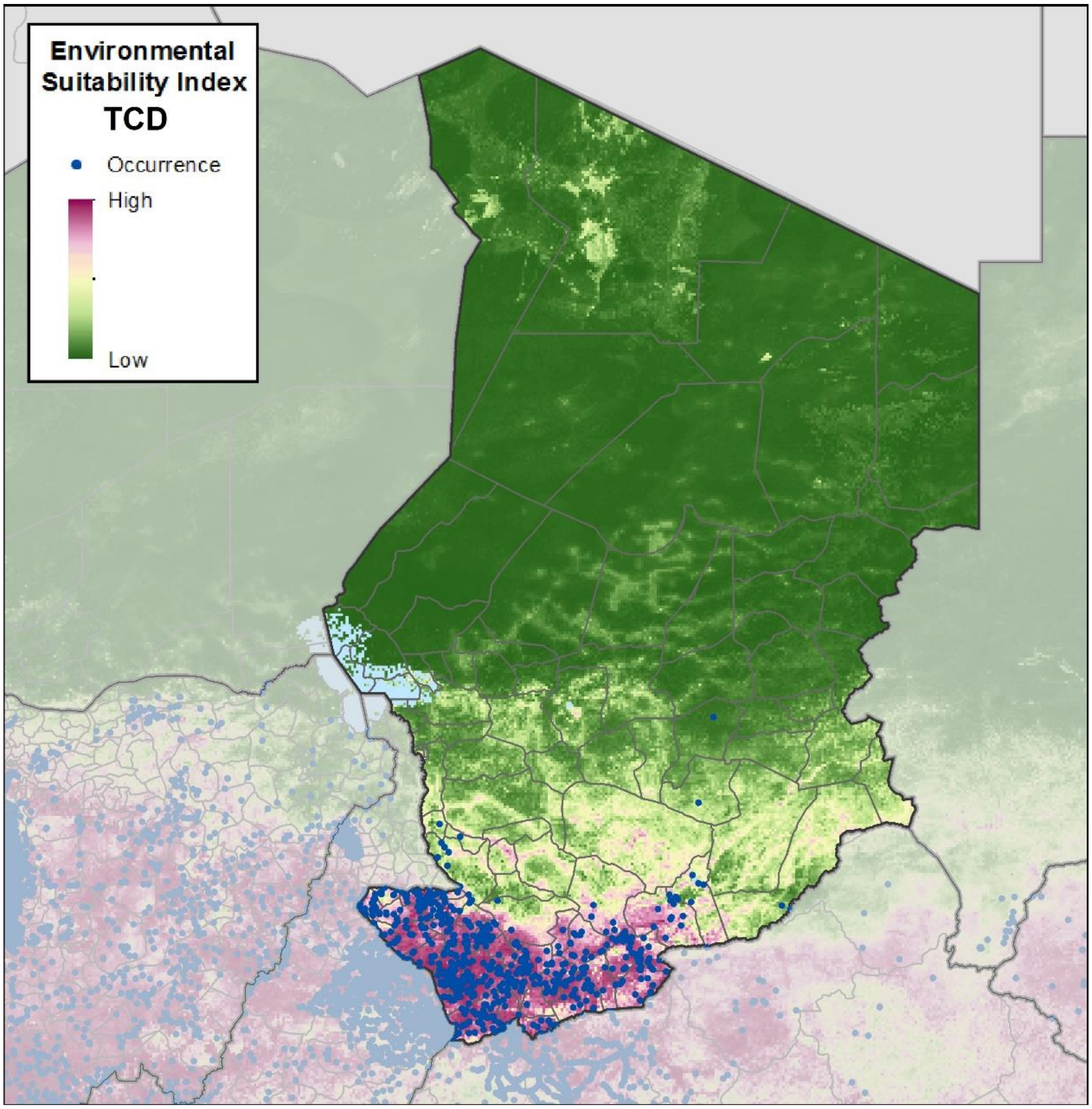

Congo

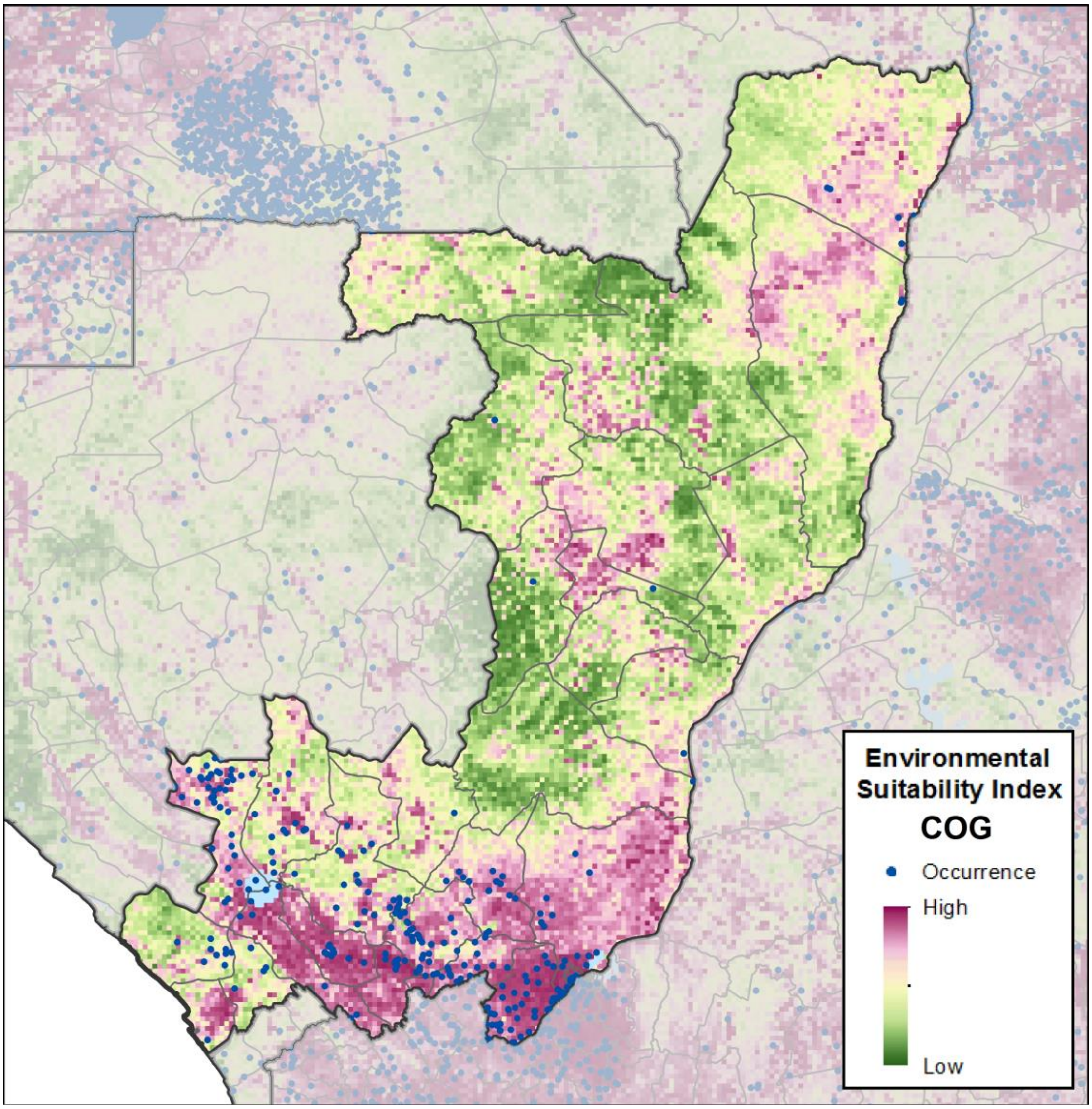

Cote d'Ivoire

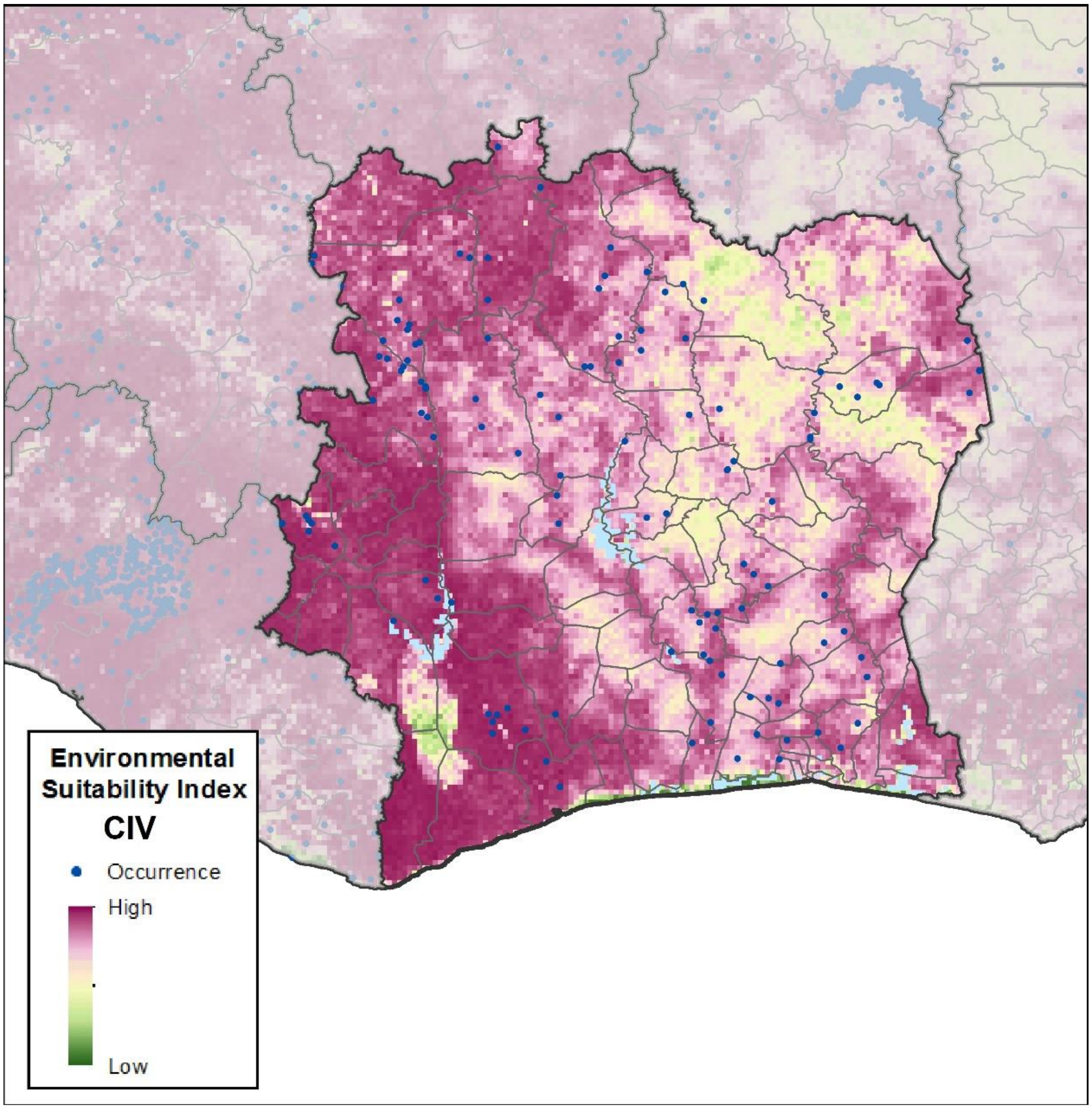

Democratic Republic of the Congo

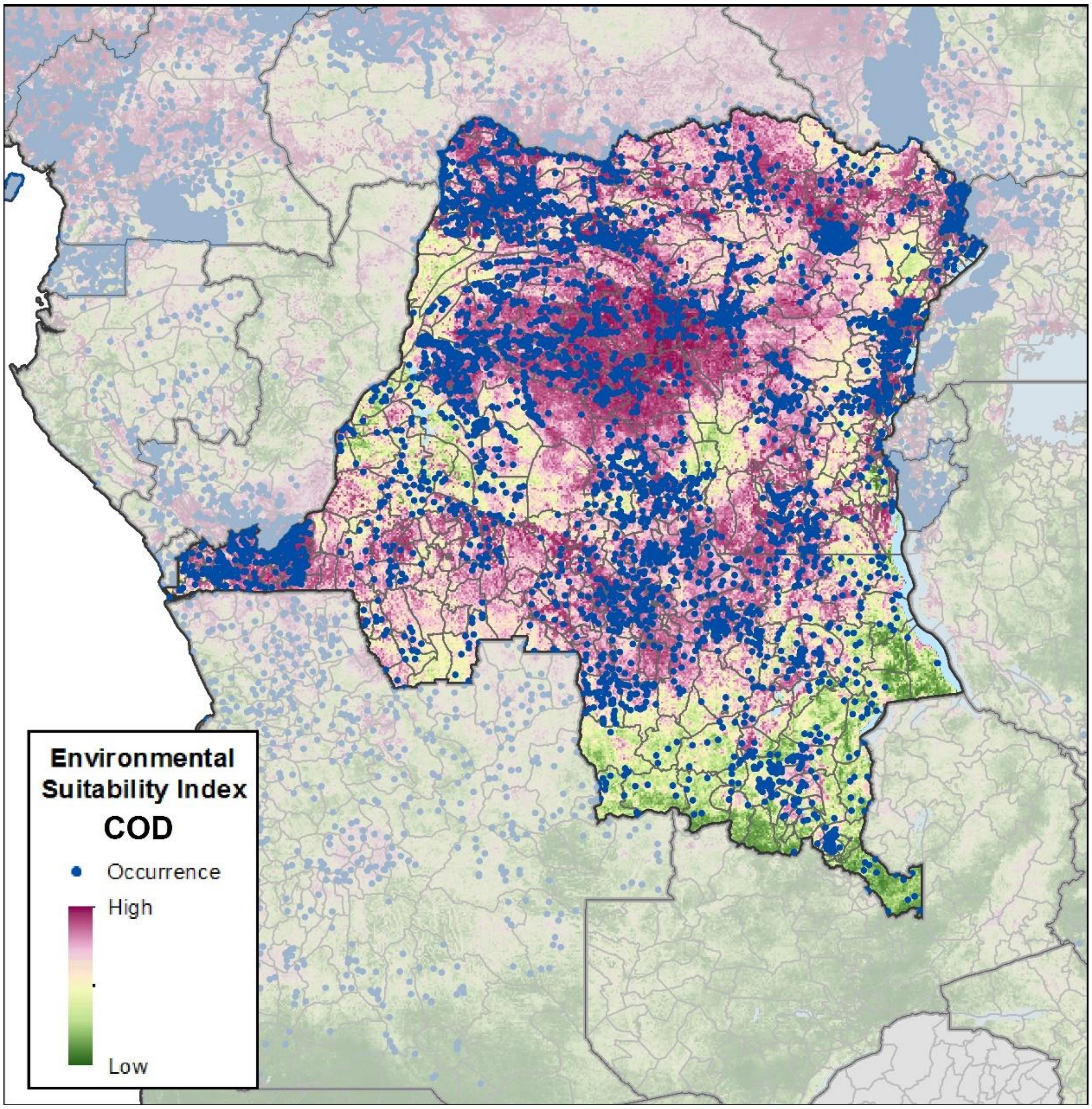

Ethiopia

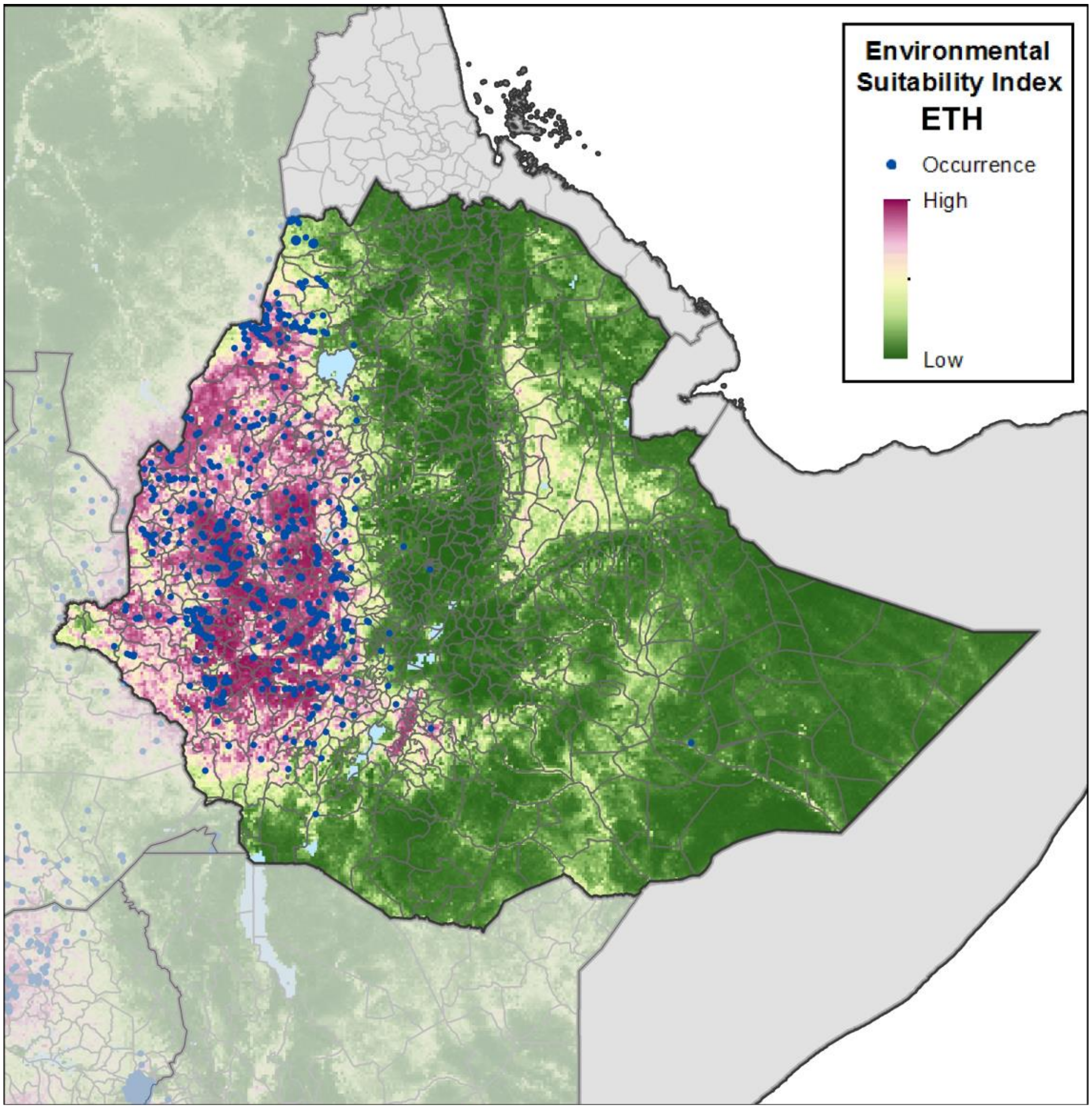

Equatorial Guinea

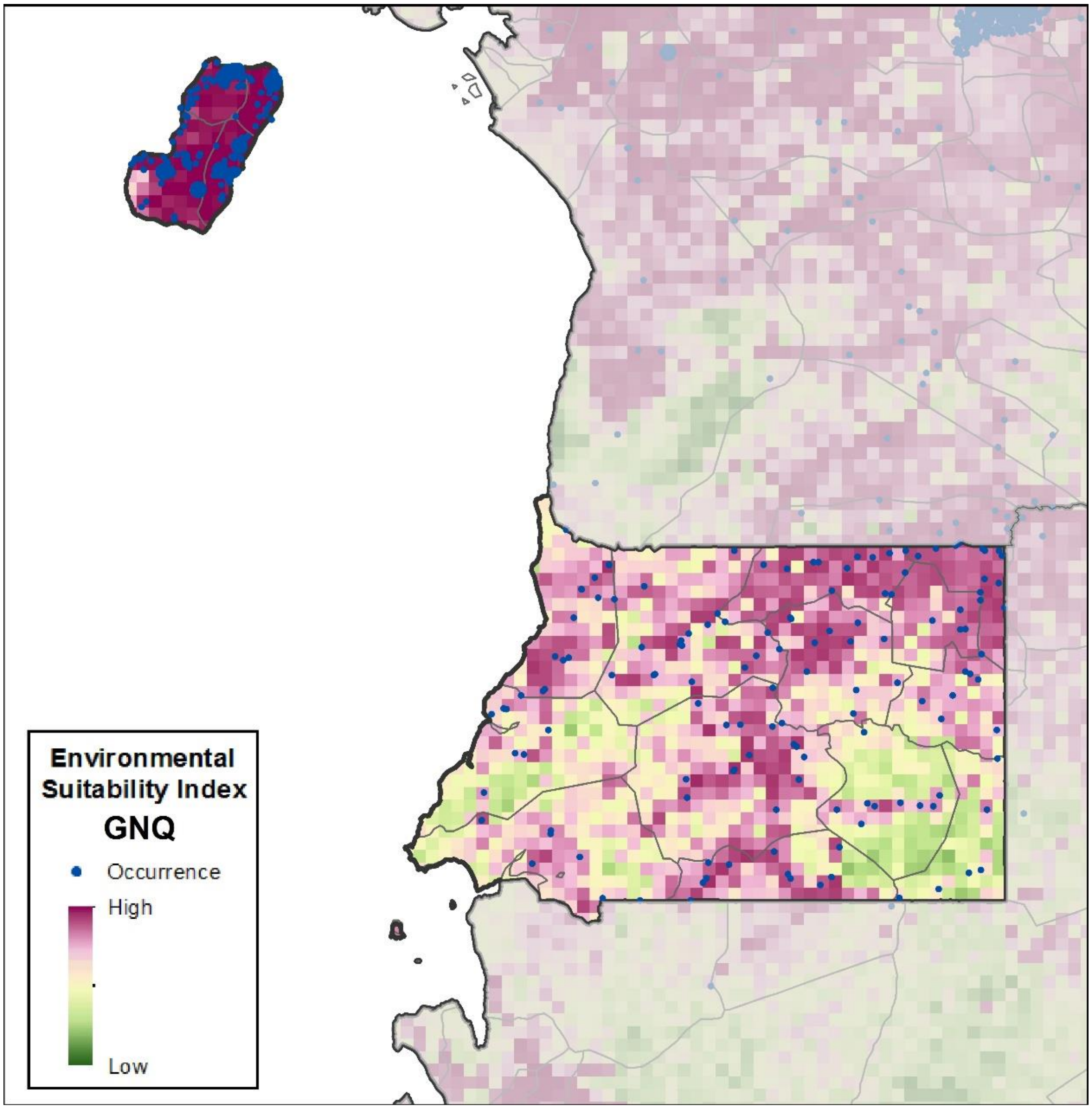

Gabon

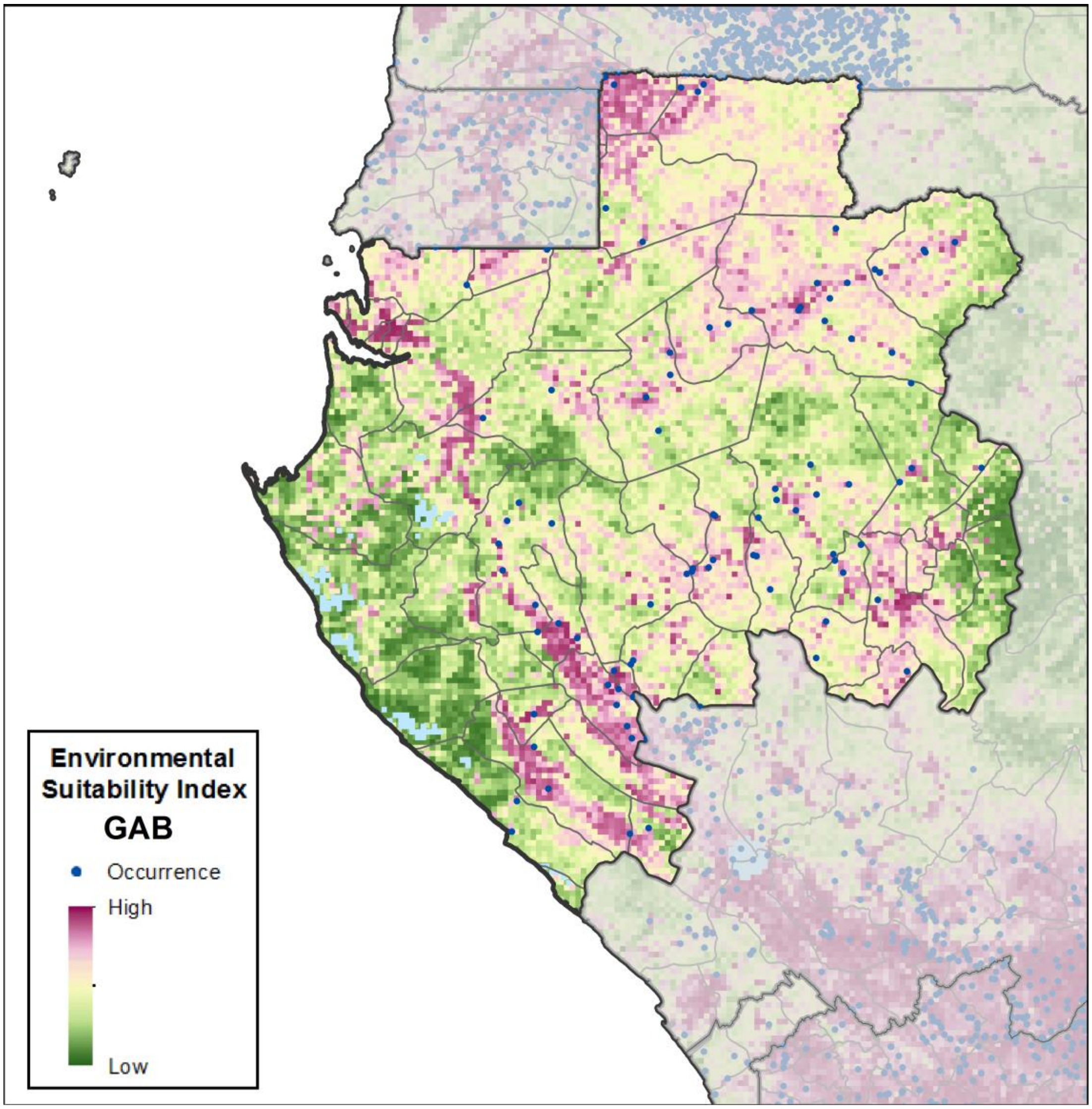

Ghana

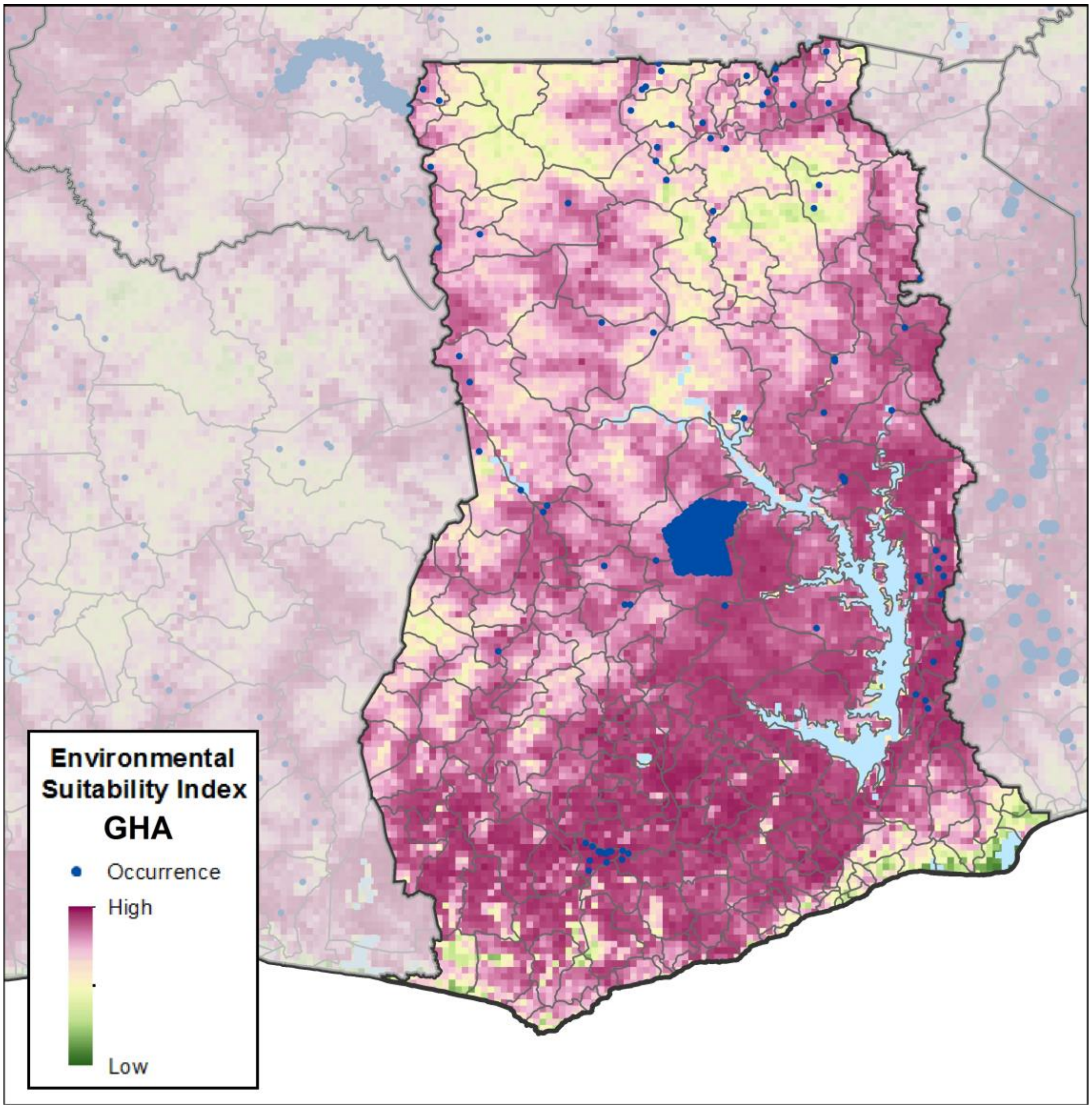

Guinea

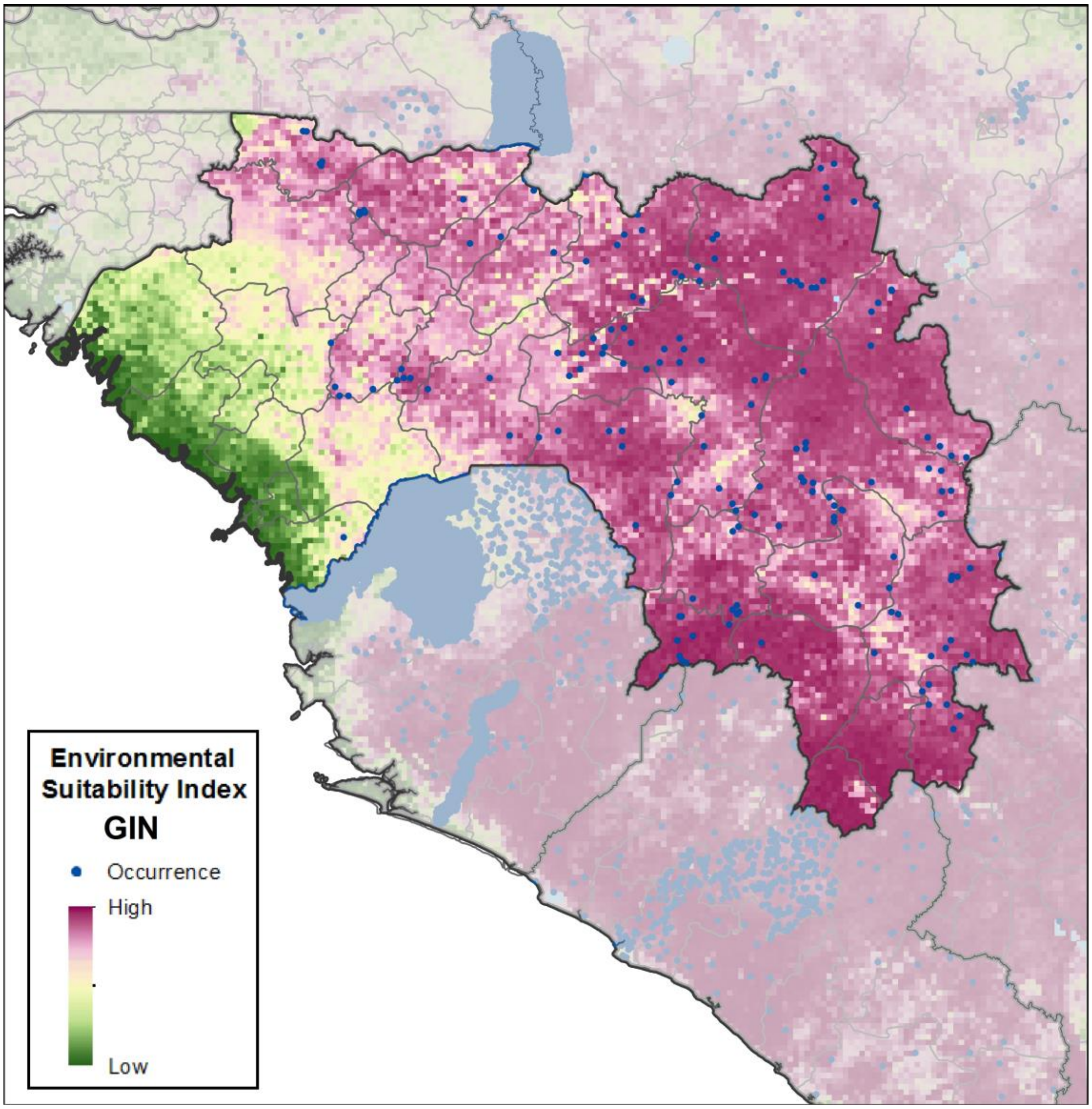

Guinea-Bisseau

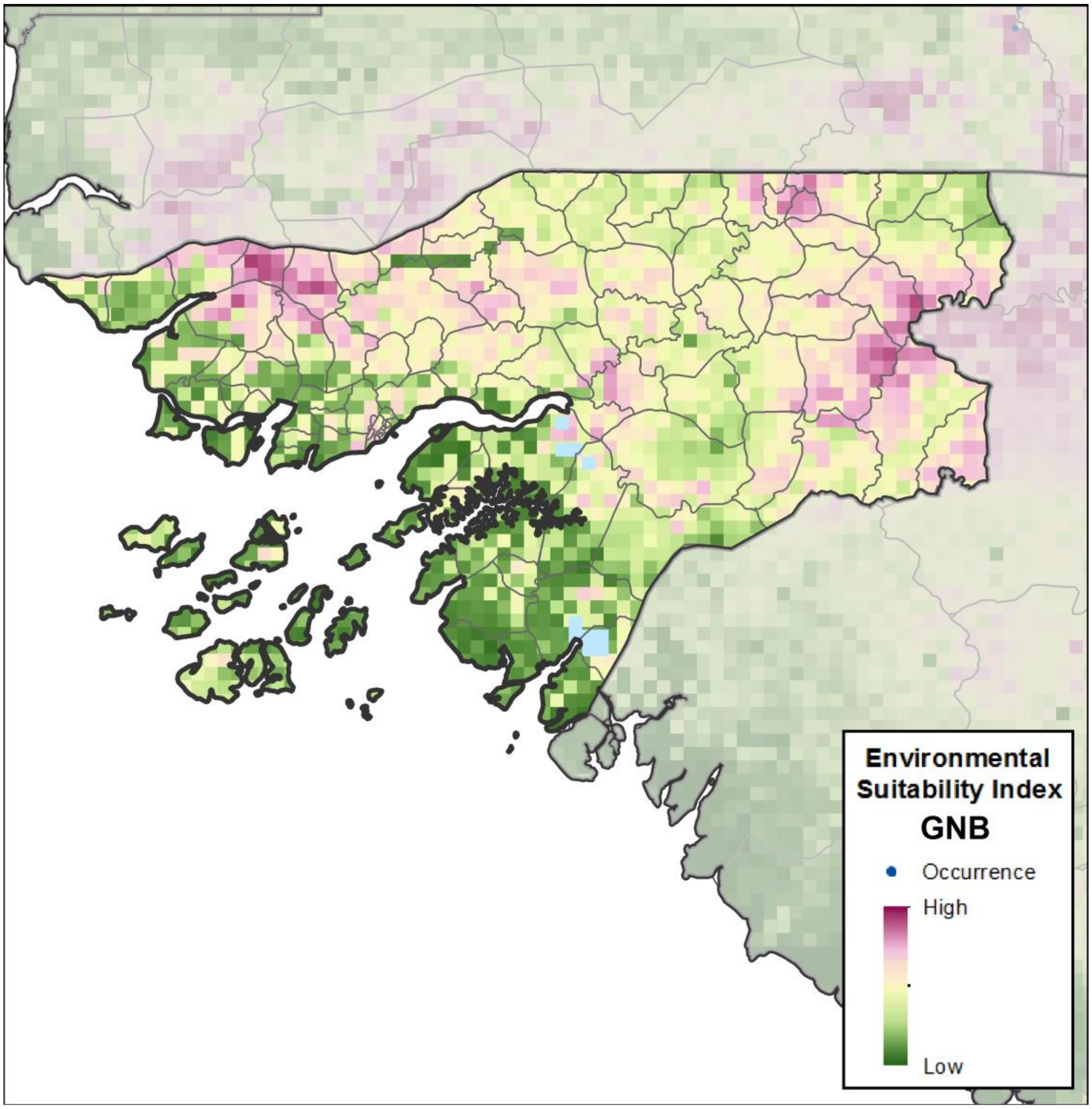

Kenya

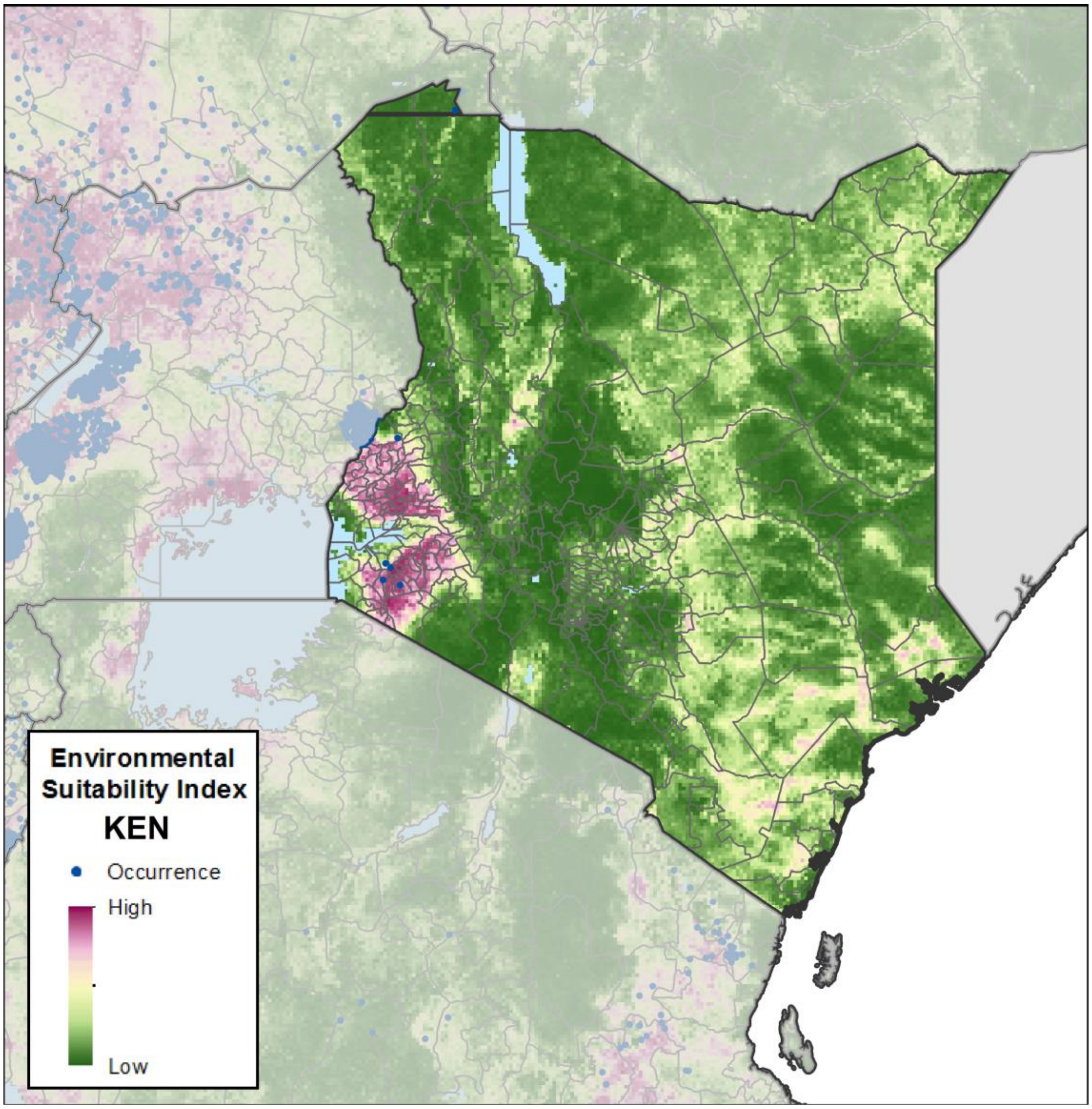

Liberia

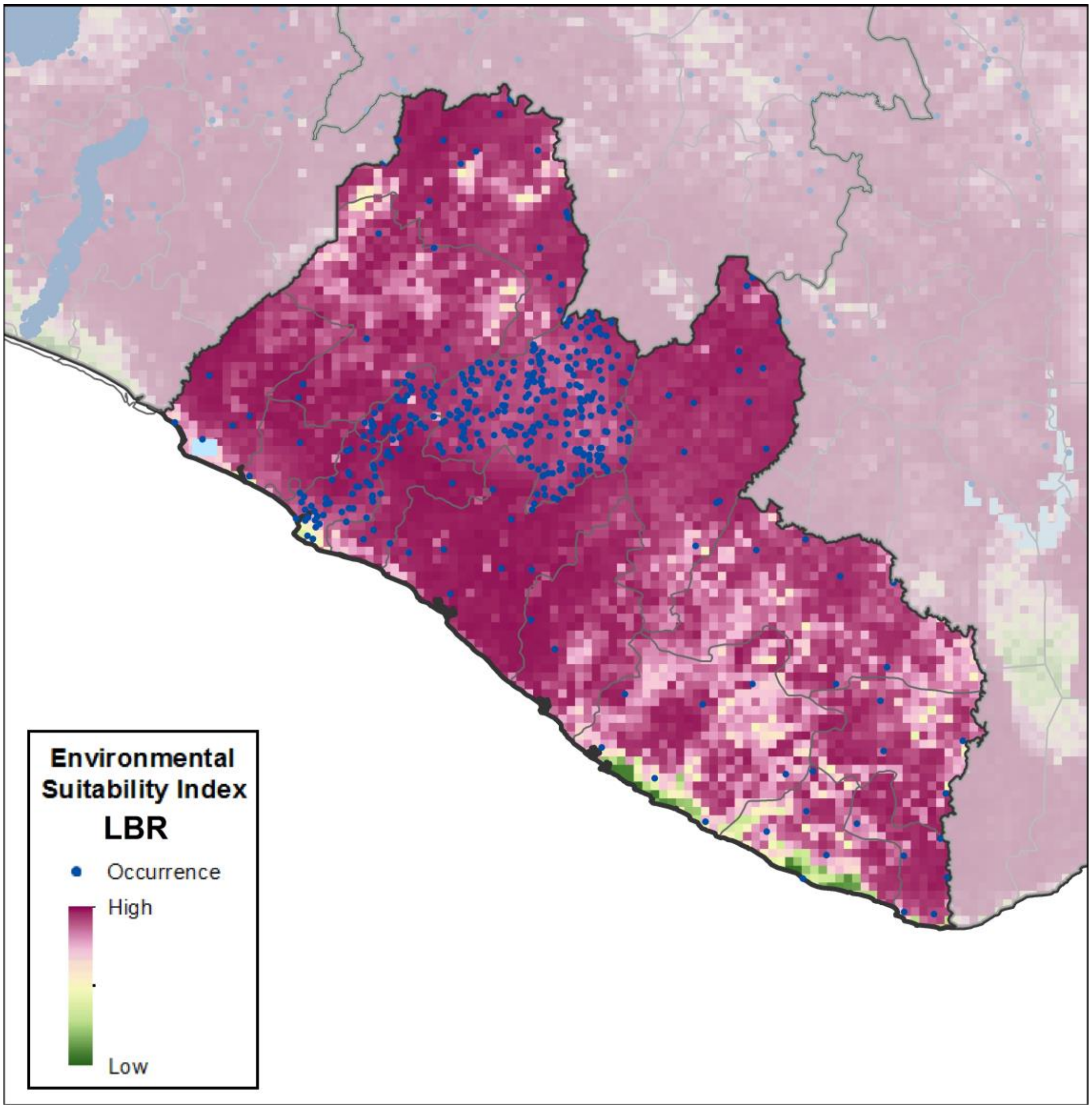

Malawi

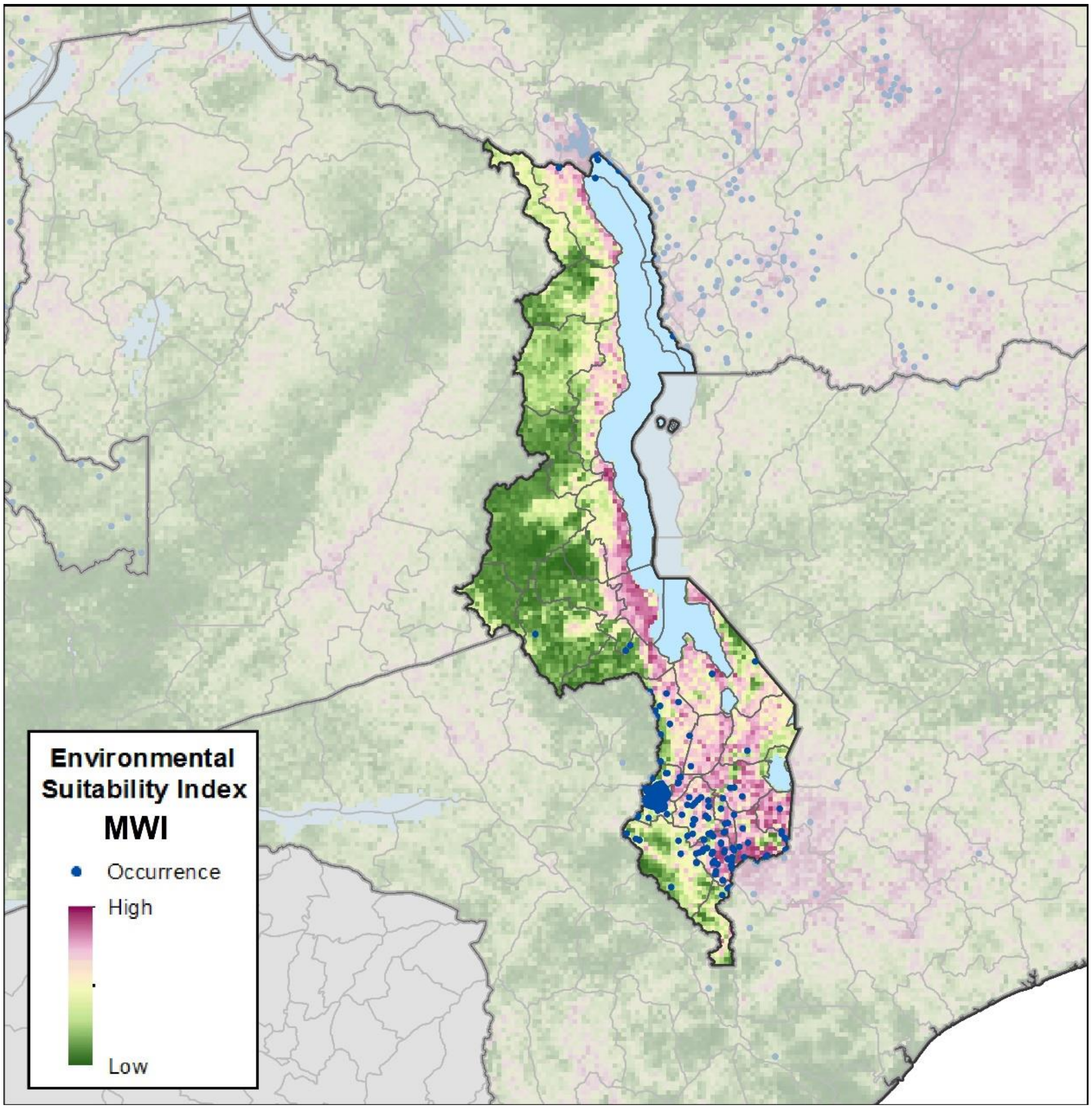

Mali

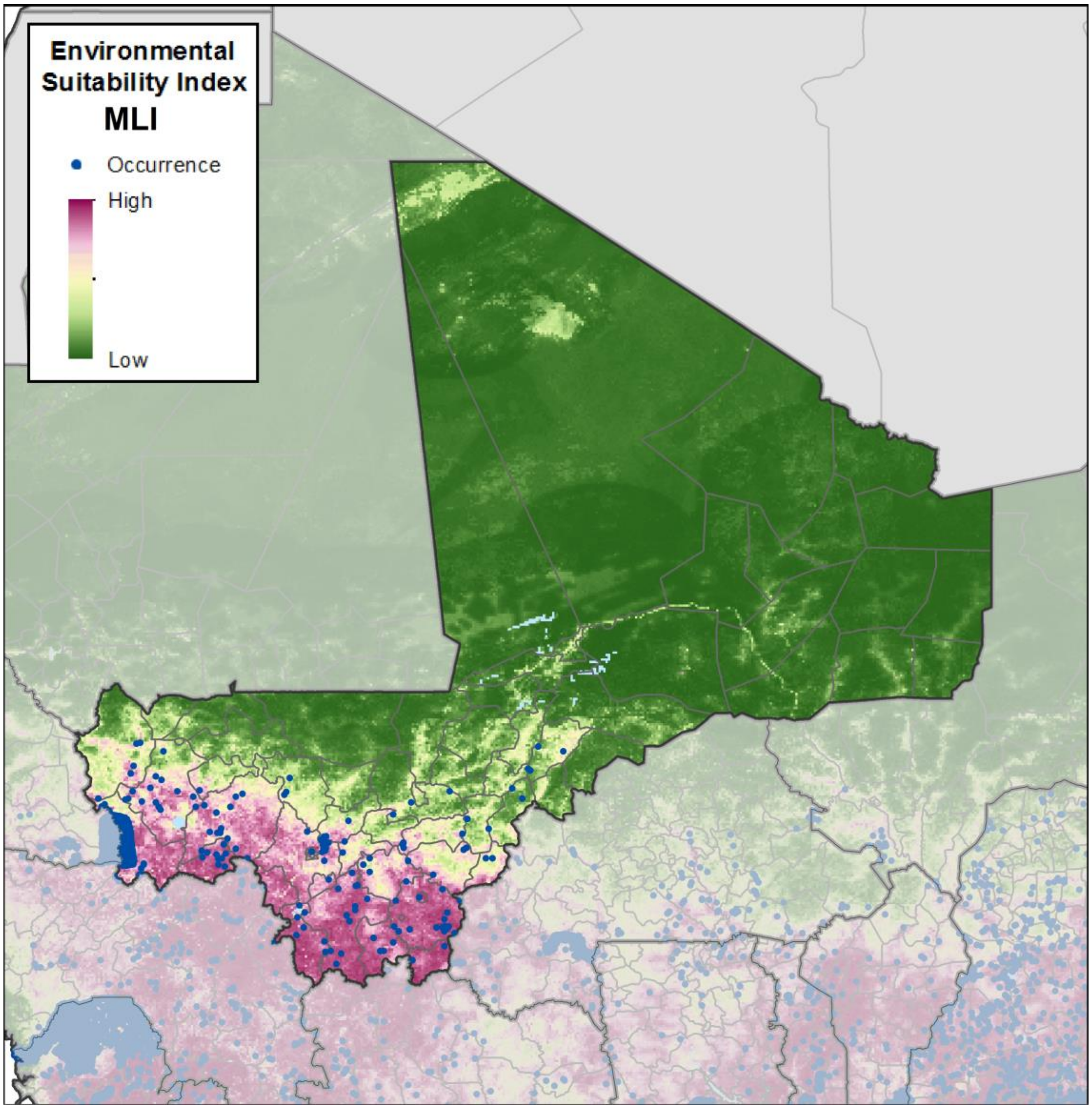

Mauritania

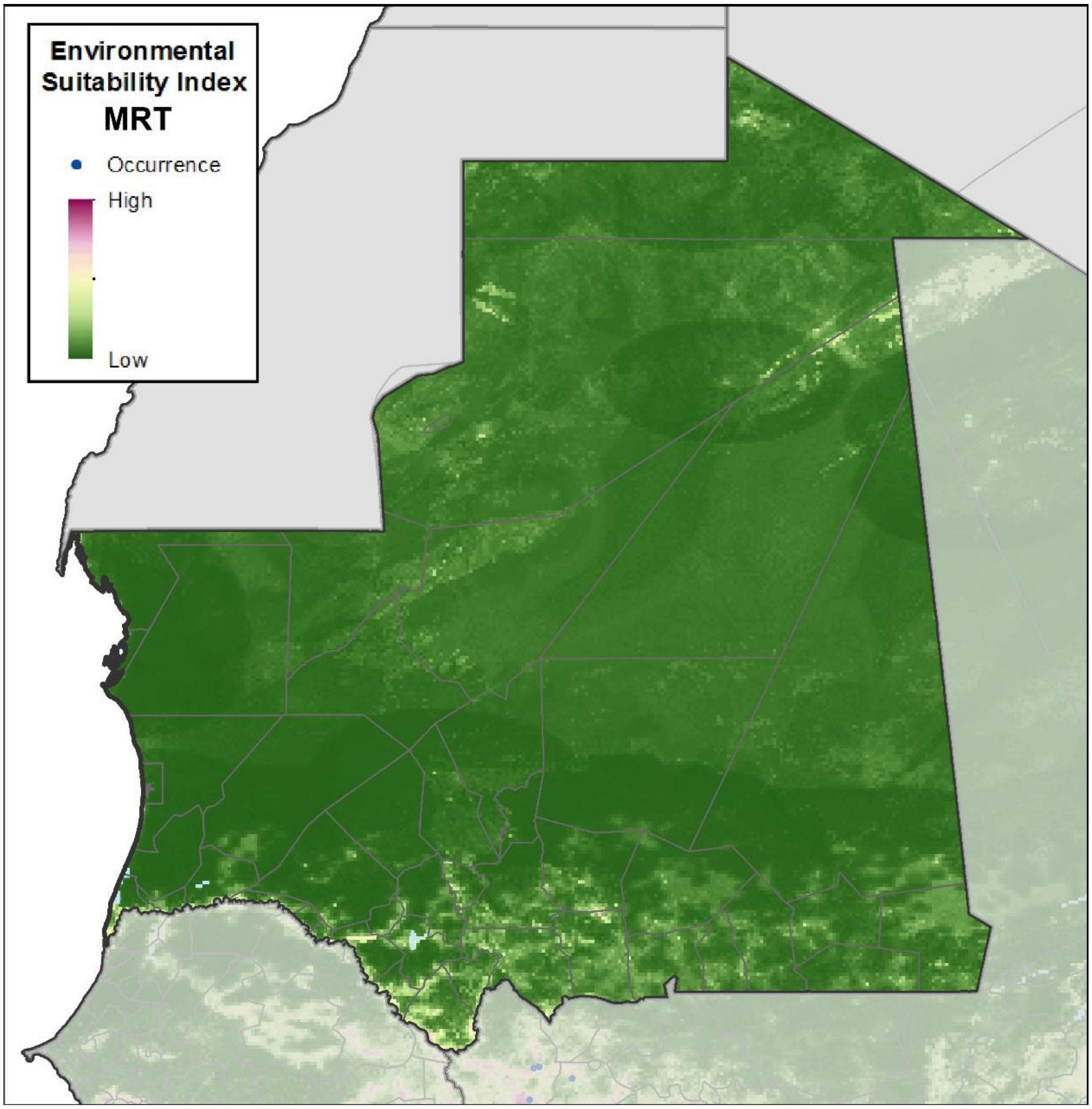

Mozambique

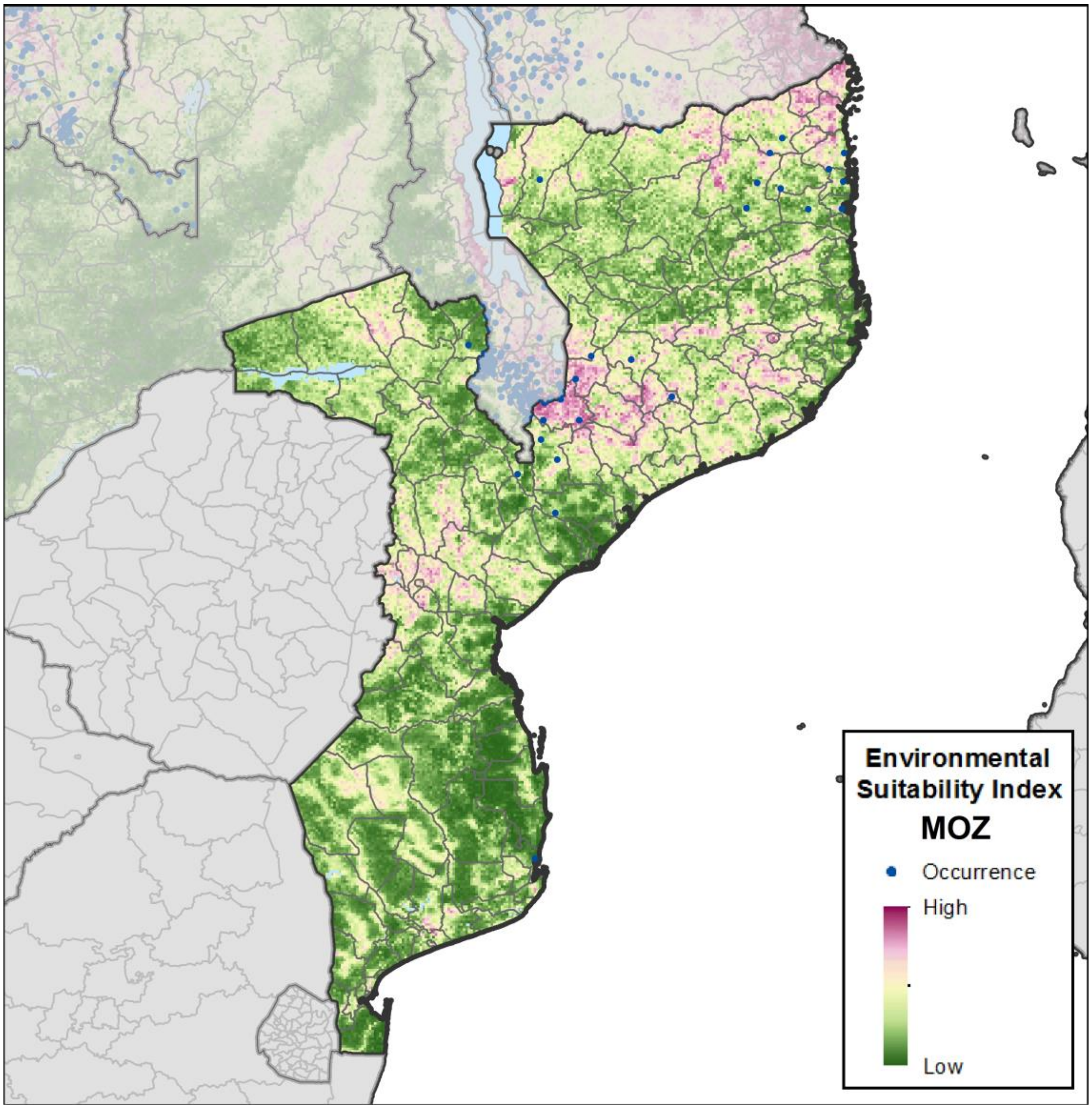

Niger

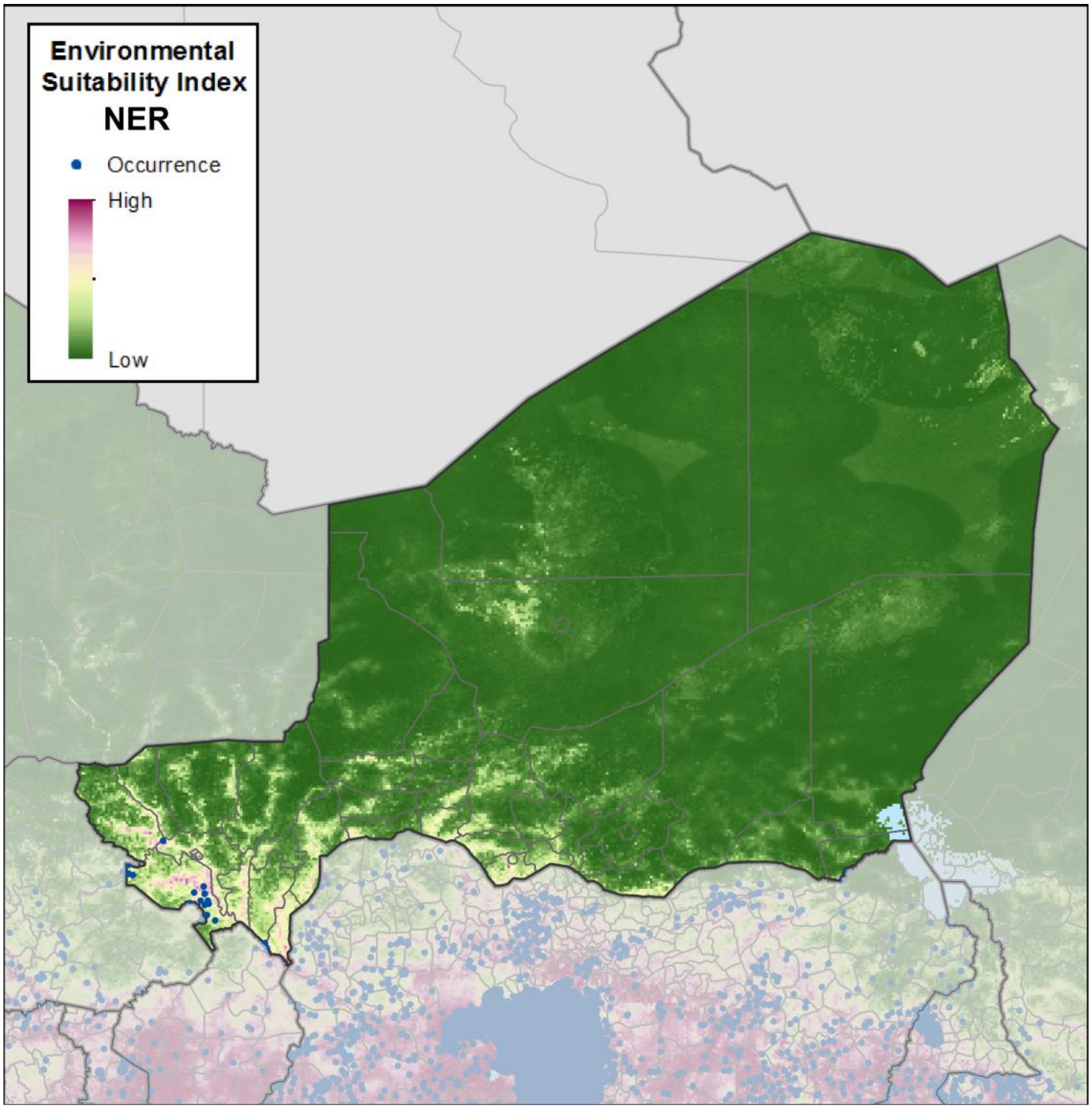

Nigeria

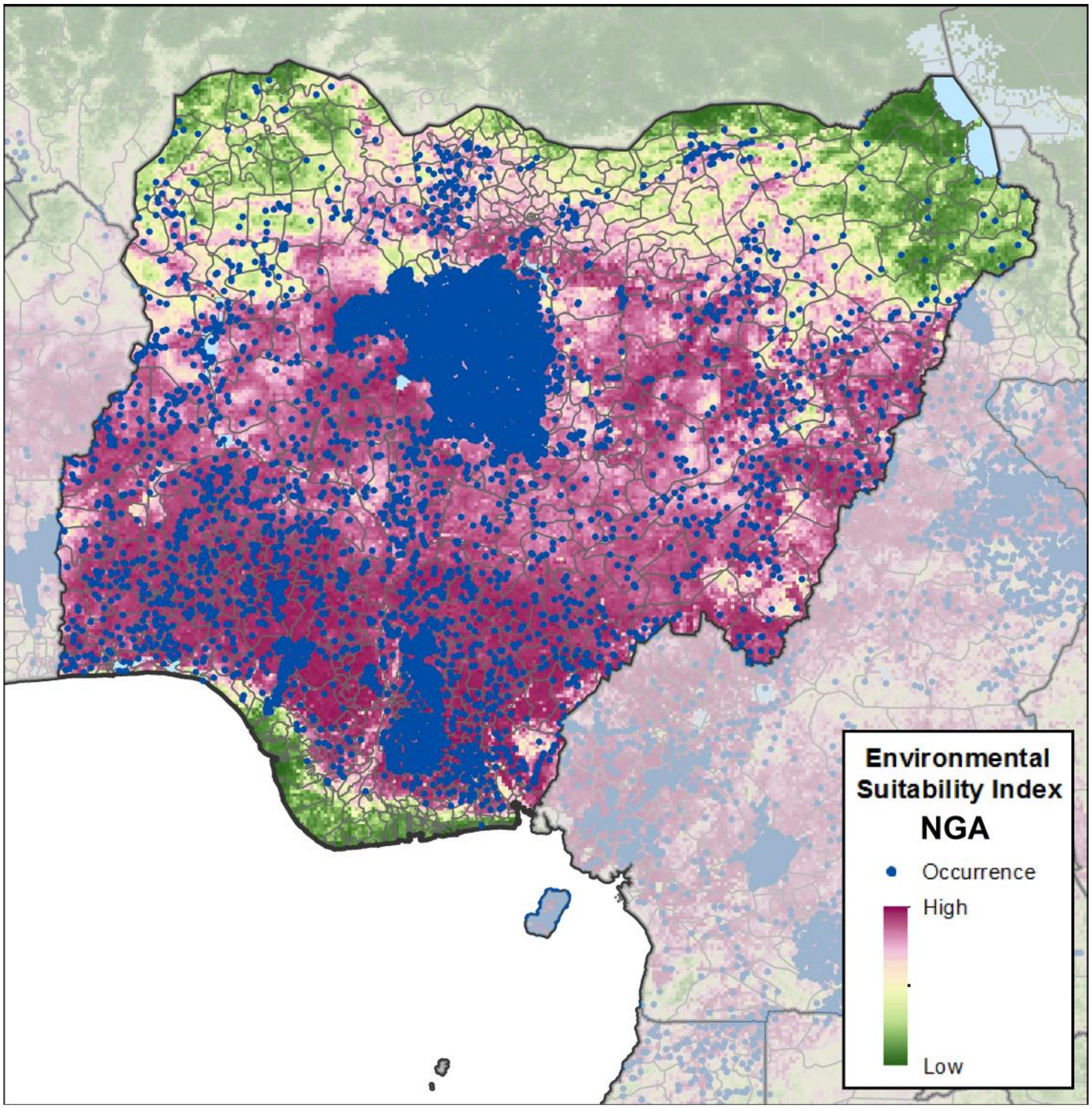

Rwanda

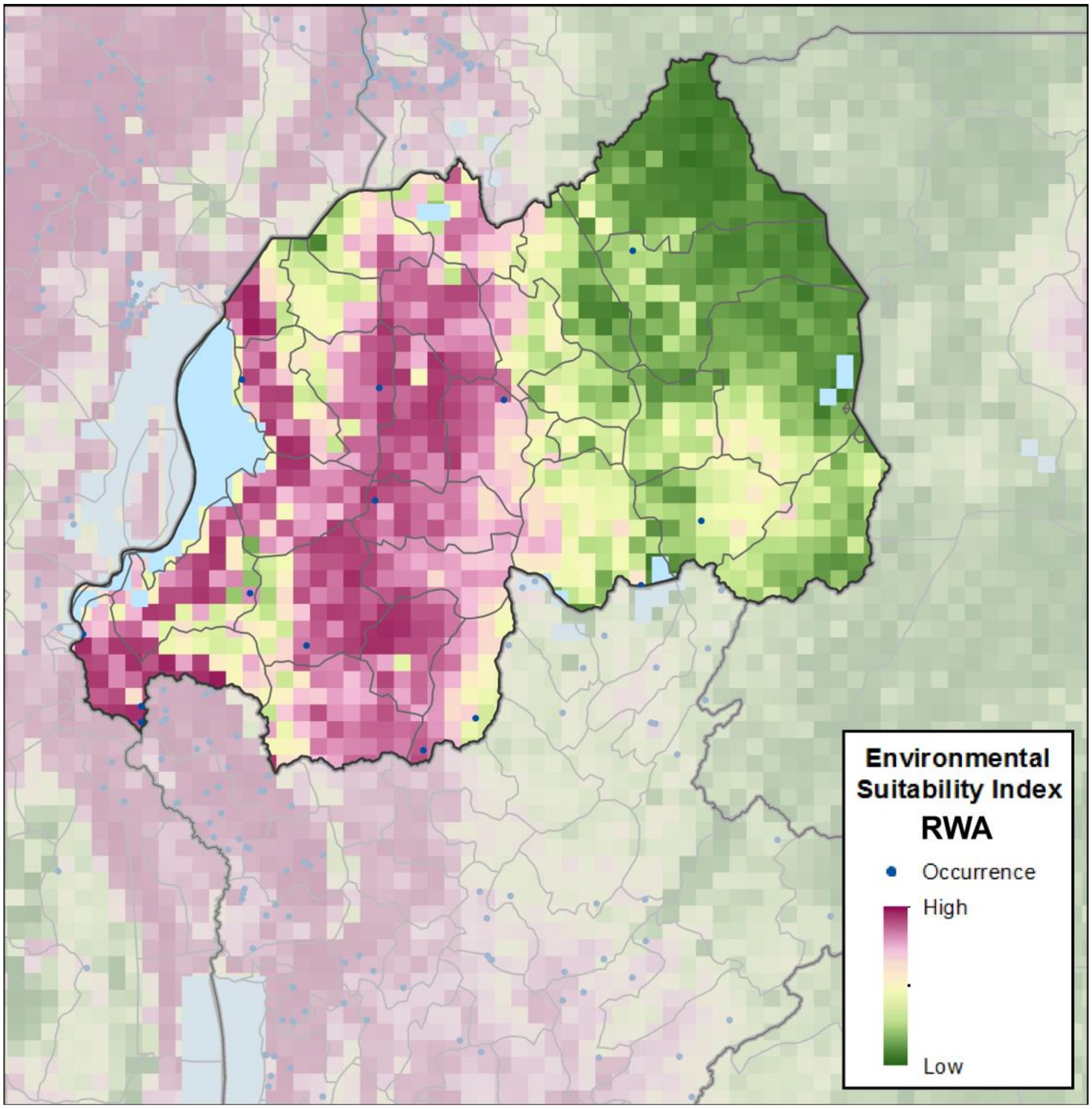

Senegal

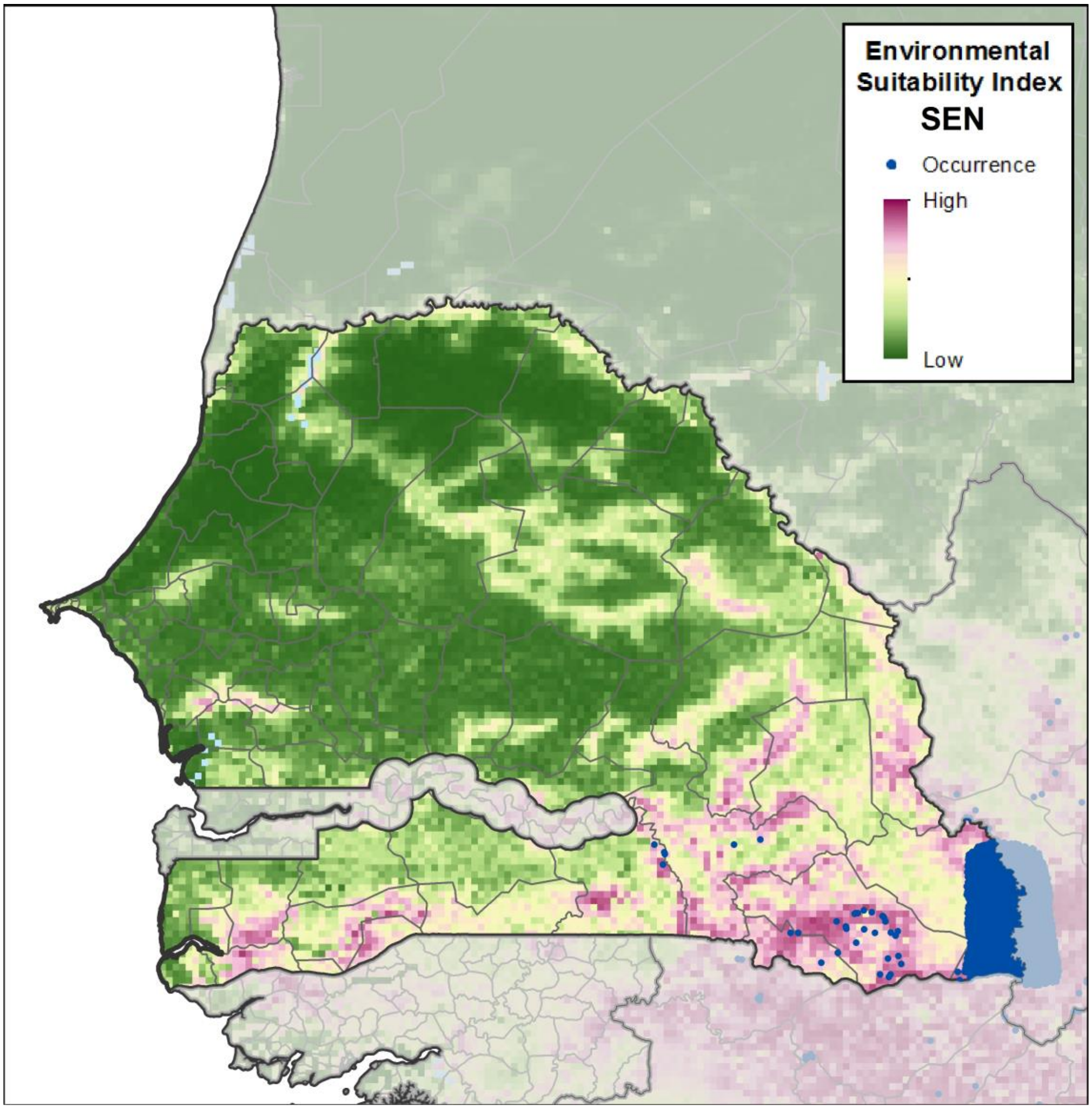

Sierra Leone

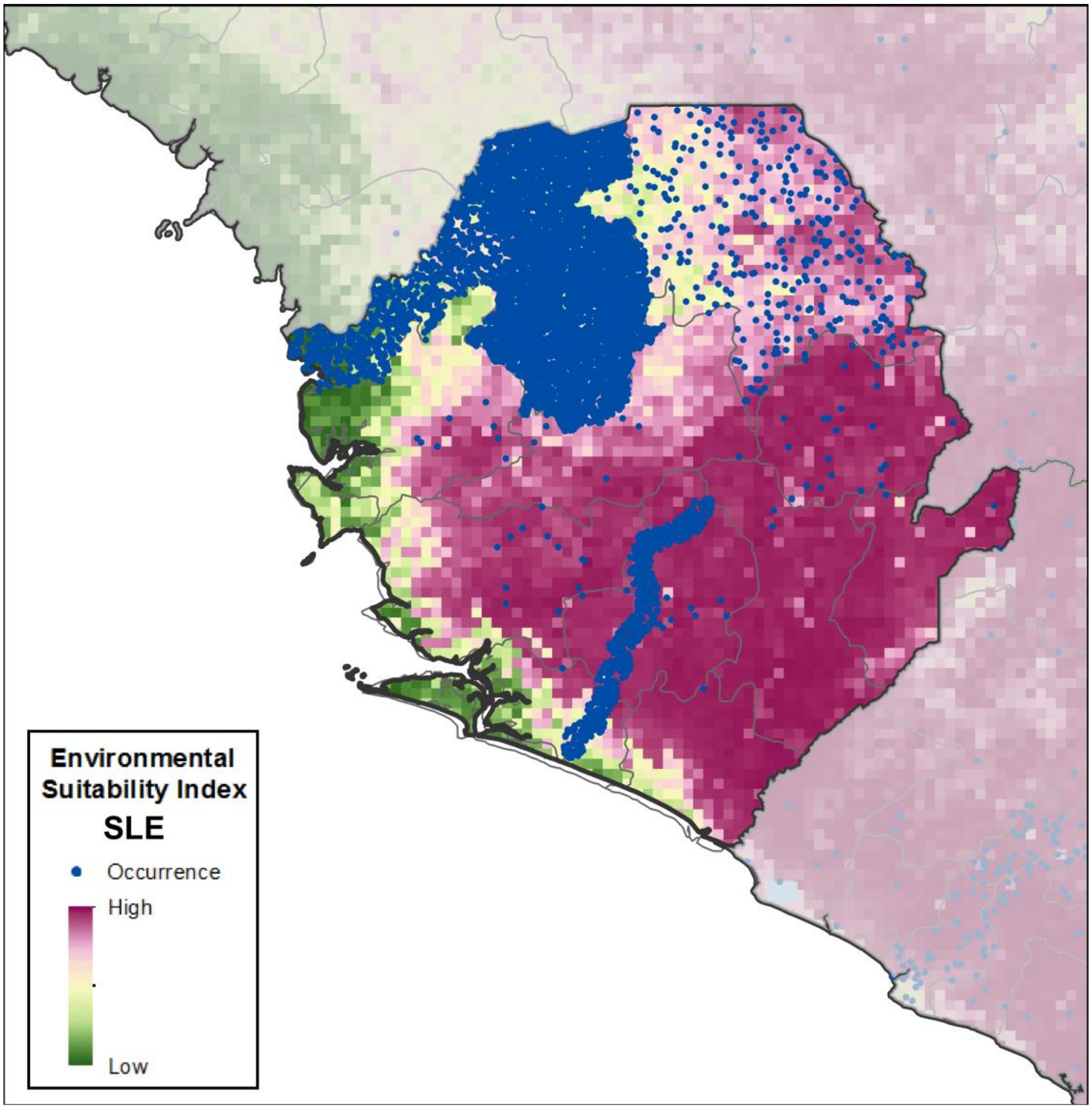

South Sudan

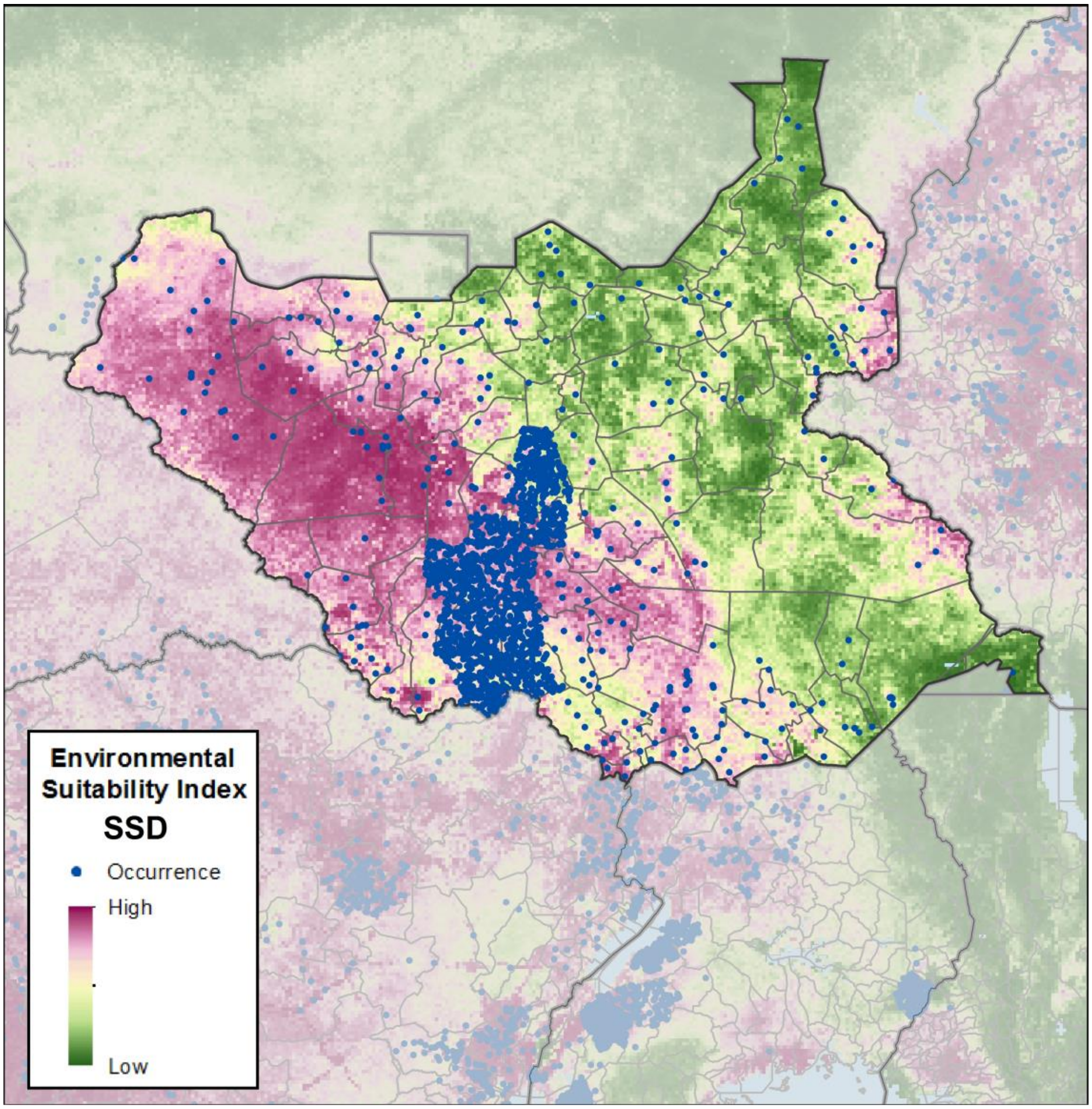

Sudan

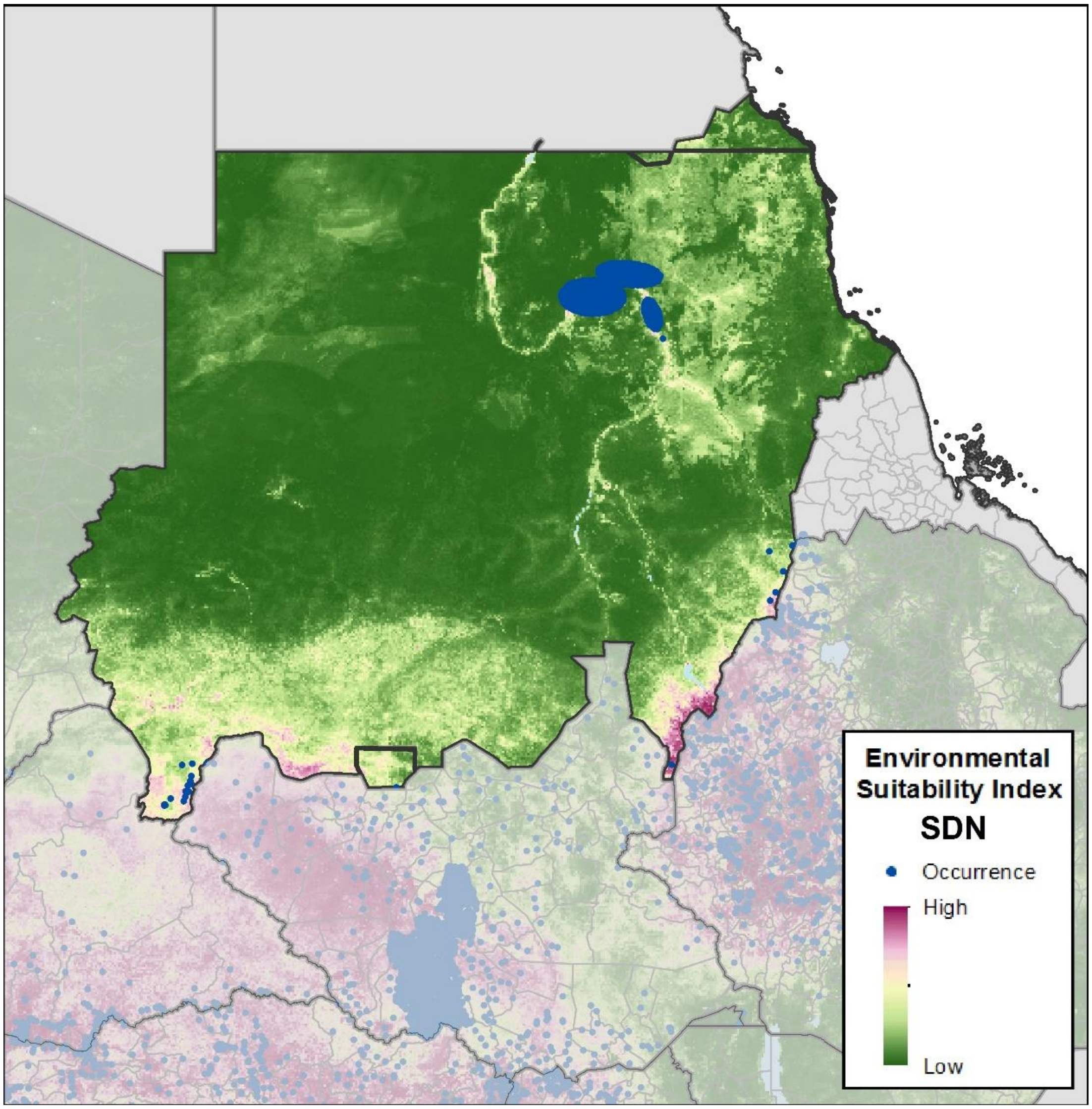

Tanzania

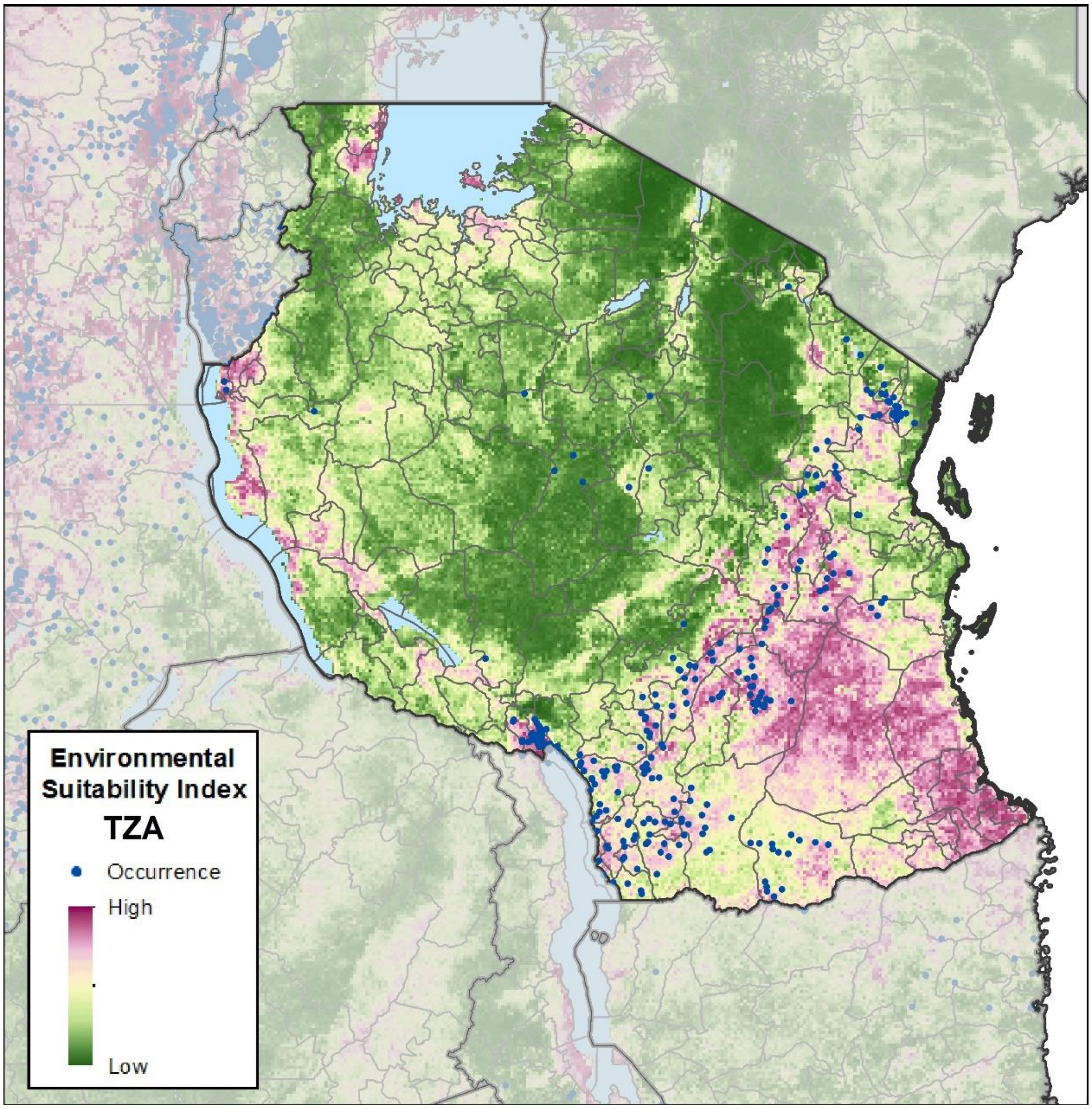

Togo

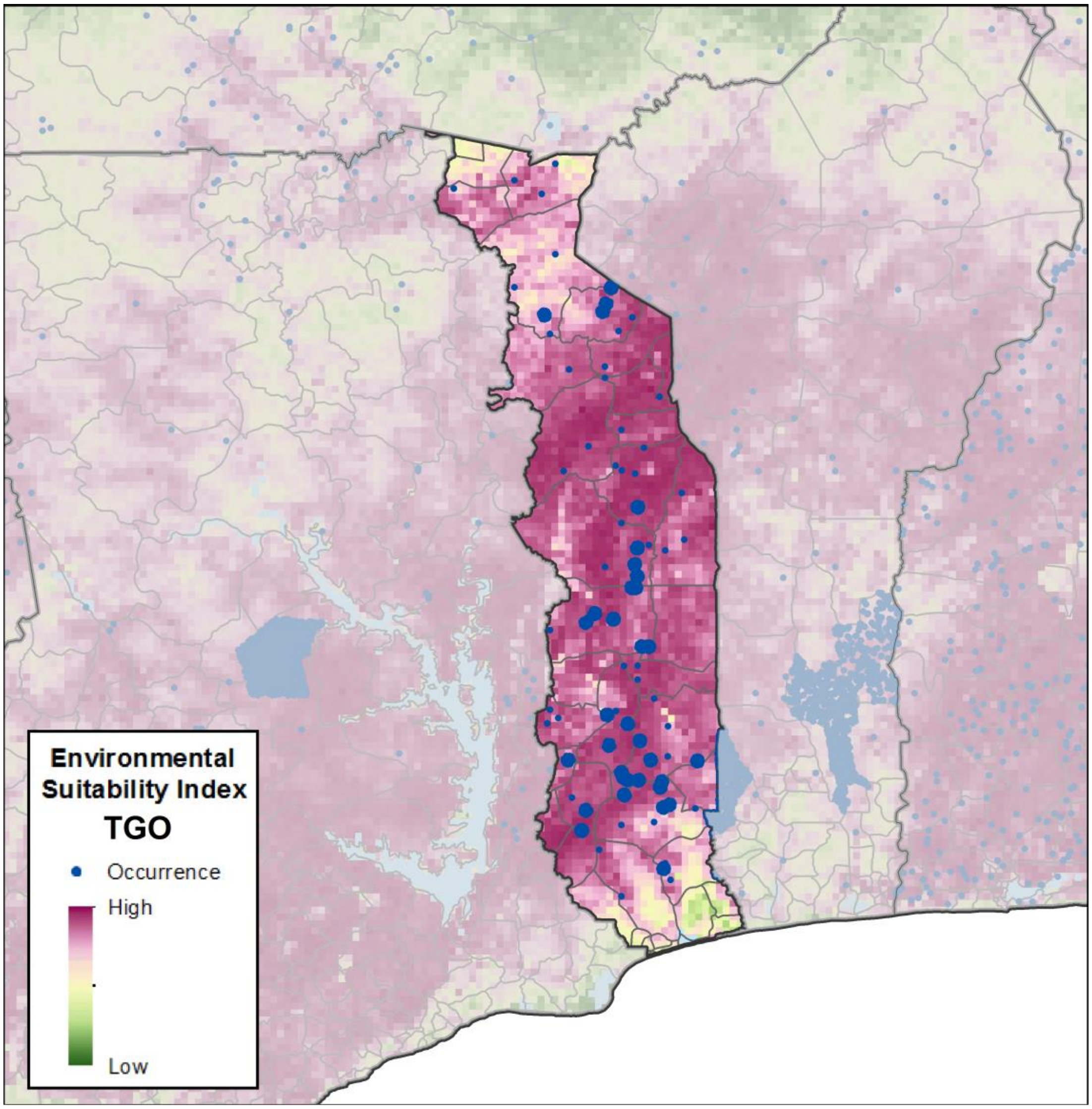

Uganda

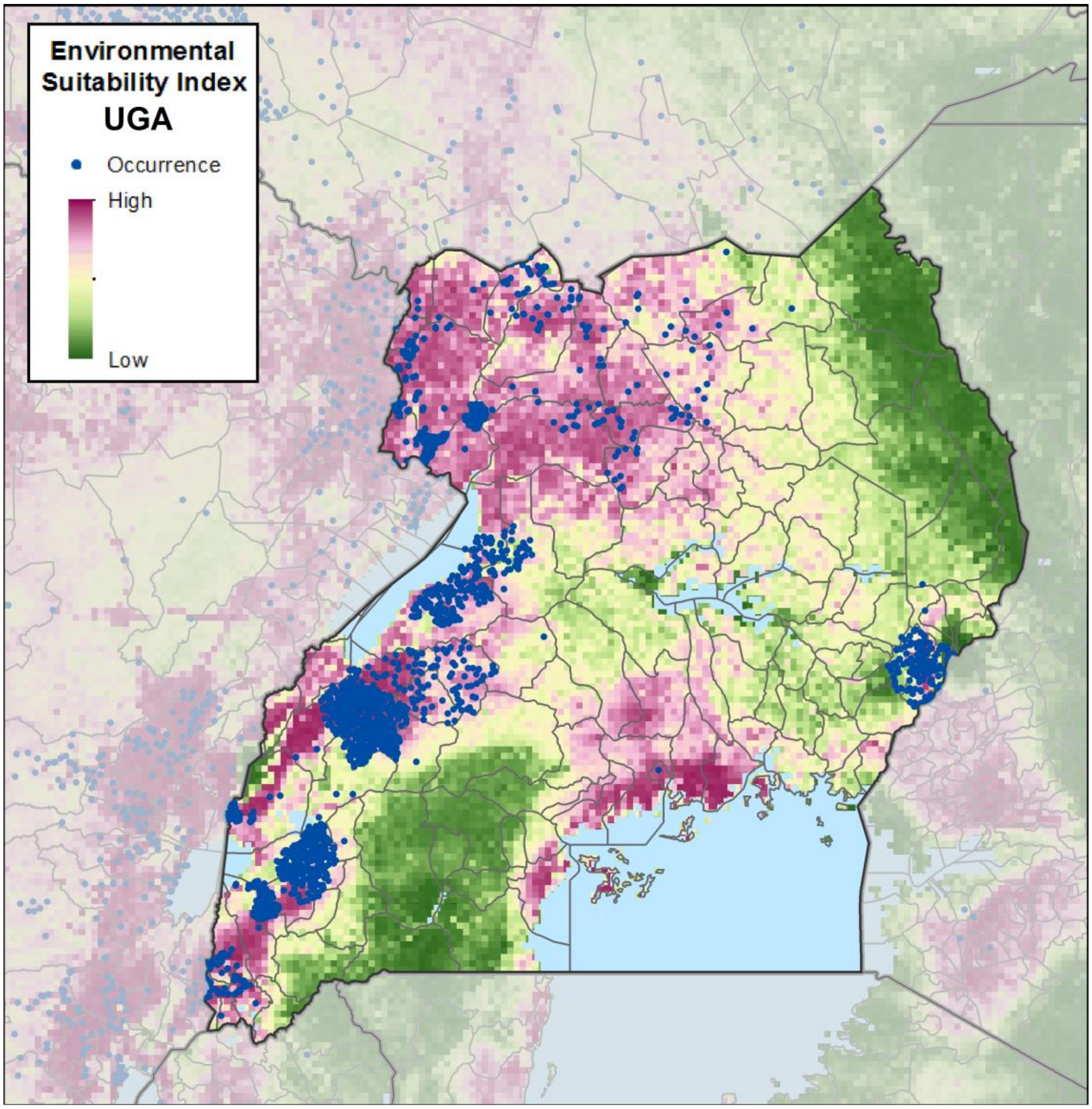

Zambia

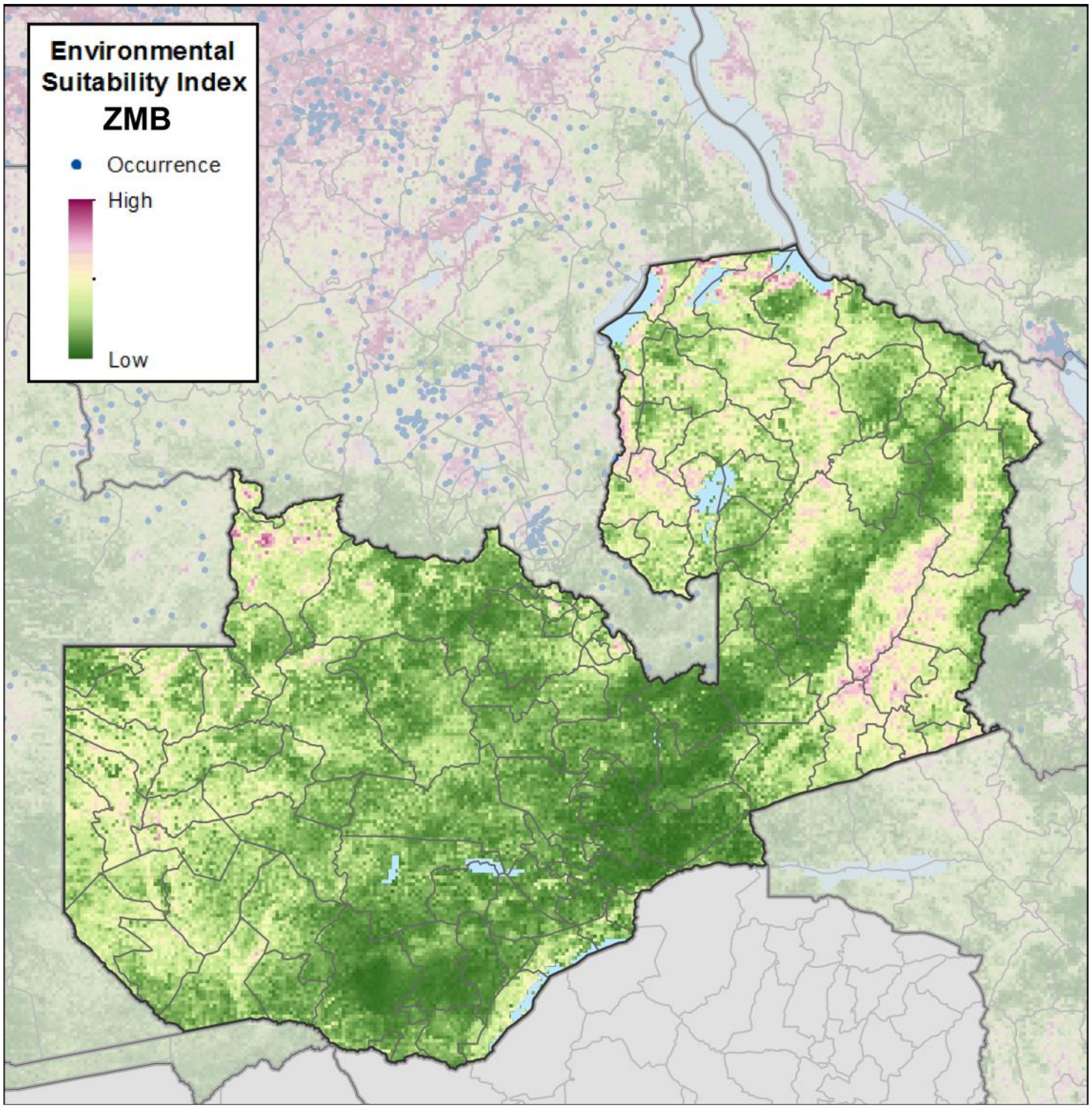

Supplement: S1 Appendix — Maps were produced using ArcGIS Desktop 10.6 and shapefiles to visualize administrative units are available at https://espen.afro.who.int/tools-resources/cartography-database. (PDF) [file pntd.0008824.s017.pdf]
